# Identification of transcription factor co-binding patterns with non-negative matrix factorization

**DOI:** 10.1093/nar/gkae743

**Published:** 2024-09-01

**Authors:** Ieva Rauluseviciute, Timothée Launay, Guido Barzaghi, Sarvesh Nikumbh, Boris Lenhard, Arnaud Regis Krebs, Jaime A Castro-Mondragon, Anthony Mathelier

**Affiliations:** Centre for Molecular Medicine Norway (NCMM), Nordic EMBL Partnership, University of Oslo, 0318 Oslo, Norway; Centre for Molecular Medicine Norway (NCMM), Nordic EMBL Partnership, University of Oslo, 0318 Oslo, Norway; European Molecular Biology Laboratory (EMBL), Genome Biology Unit, Meyerhofstraße 1, 69117 Heidelberg, Germany; Collaboration for Joint Ph.D. degree between EMBL and Heidelberg University, Heidelberg, Germany; MRC London Institute of Medical Sciences, Du Cane Road, London W12 0NN, UK; Institute of Clinical Sciences, Faculty of Medicine, Imperial College London, Hammersmith Hospital Campus, Du Cane Road, London W12 0NN, UK; MRC London Institute of Medical Sciences, Du Cane Road, London W12 0NN, UK; Institute of Clinical Sciences, Faculty of Medicine, Imperial College London, Hammersmith Hospital Campus, Du Cane Road, London W12 0NN, UK; European Molecular Biology Laboratory (EMBL), Genome Biology Unit, Meyerhofstraße 1, 69117 Heidelberg, Germany; Centre for Molecular Medicine Norway (NCMM), Nordic EMBL Partnership, University of Oslo, 0318 Oslo, Norway; Centre for Molecular Medicine Norway (NCMM), Nordic EMBL Partnership, University of Oslo, 0318 Oslo, Norway; Department of Medical Genetics, Institute of Clinical Medicine, University of Oslo and Oslo University Hospital, Oslo, Norway; Center for Bioinformatics, Department of Informatics, Faculty of Mathematics and Natural Sciences, University of Oslo, Oslo, Norway

## Abstract

Transcription factor (TF) binding to DNA is critical to transcription regulation. Although the binding properties of numerous individual TFs are well-documented, a more detailed comprehension of how TFs interact cooperatively with DNA is required. We present COBIND, a novel method based on non-negative matrix factorization (NMF) to identify TF co-binding patterns automatically. COBIND applies NMF to one-hot encoded regions flanking known TF binding sites (TFBSs) to pinpoint enriched DNA patterns at fixed distances. We applied COBIND to 5699 TFBS datasets from UniBind for 401 TFs in seven species. The method uncovered already established co-binding patterns and new co-binding configurations not yet reported in the literature and inferred through motif similarity and protein-protein interaction knowledge. Our extensive analyses across species revealed that 67% of the TFs shared a co-binding motif with other TFs from the same structural family. The co-binding patterns captured by COBIND are likely functionally relevant as they harbor higher evolutionarily conservation than isolated TFBSs. Open chromatin data from matching human cell lines further supported the co-binding predictions. Finally, we used single-molecule footprinting data from mouse embryonic stem cells to confirm that the COBIND-predicted co-binding events associated with some TFs likely occurred on the same DNA molecules.

## Graphical Abstract

**Graphical Abstract:**
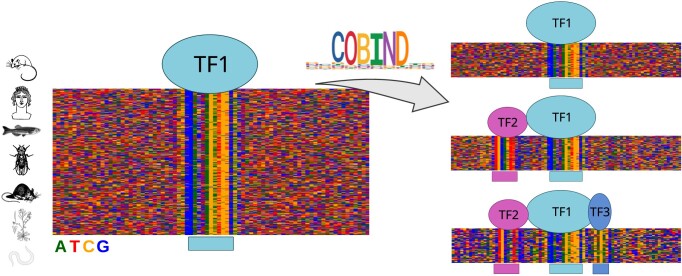

## Introduction

The interactions between DNA and transcription factors (TFs) are crucial to transcription regulation as they intrinsically determine cell growth, development, and response to stimuli. TFs bind DNA at TF binding sites (TFBSs) in a sequence-specific manner through direct contact between DNA nucleotides and amino acids of the TFs’ DNA-binding domain (DBD) (1). Structural similarities between DBDs classify TFs into structural classes and families, and TFs from the same class or family usually recognize similar DNA patterns (or motifs).

The binding properties of individual TFs have been vastly studied (2–4), and several databases store DNA binding profiles for TFs across multiple taxonomic groups (e.g. JASPAR, CIS-BP and HOCOMOCO (5–7)). While these databases primarily provide TF binding motifs for individual TFs, there is a need to increase our understanding of how TFs cooperatively bind DNA to regulate transcription (8). The cooperative binding of TFs generates many possible binding combinations, thus increasing the complexity of gene regulatory networks (8,9). Recent studies argue for a flexible grammar of motifs at *cis-*regulatory regions, where the spacing and orientation between TFBSs would not be a key determinant for transcription regulation (10–13). Nevertheless, some TFs physically cooperate, providing a strict motif syntax important for transcription regulation. For instance, exhaustively testing the combinatorics of liver-associated TFBSs with massively parallel reporter assays revealed that TFBS orientation and order are major drivers of gene regulatory activity (14). A well-characterized example of physical interaction between TFs is POU5F1 (OCT4) cooperation with either SOX2 or SOX17 in pluripotent cells. The spacing between the binding sites will determine POU5F1’s partner and the corresponding regulatory effect (15,16). Hence, the specific spacing and orientation between the canonical motifs recognized by two TFs can characterize their co-binding at given genomic regions (17,18). However, the cooperative binding of two TFs can also slightly modify their individual DNA sequence preference (19). Consequently, systematically identifying cooperative events genome-wide with strict binding grammar is still challenging.

The CAP-SELEX experimental technique captures co-binding events between predefined sets of TFs *in vitro* (19). However, whether the same co-binding properties will occur *in vivo* remains to be determined. Therefore, computational methods, such as TF-COMB, TACO, SpaMo and MCOT (20–23), leverage *in vivo* data, such as ChIP-seq (24), to predict co-binding events. These tools rely on identifying the co-occurrence of pre-defined genomic regions bound by the individual TFs or already known individual DNA binding motifs in predefined genomic regions. While these strategies reduce the search space, they restrict discoveries for the pairs of TFs where either bound regions or TF binding motifs exist for both TFs. When relying on already-known TF binding motifs, the quality of the available motif collections is a limiting factor, and this approach cannot discover new DNA sequence patterns. Another approach implemented in the RSAT *dyad-analysis* tool does not rely on pre-defined motifs and predicts spaced pairs of motifs *de novo* starting from spaced 3-mer patterns (25,26). Finally, deep learning approaches can infer regulatory patterns from experimental data, with the capacity to pinpoint TF co-binding events (12,27). Even though the underlying algorithms are advanced and powerful, their complexity and interpretability make it challenging to characterize specific co-binding events without extensive *a posteriori* analyses.

In this study, we aimed to discover TF co-binding patterns in the vicinity of high-quality TFBSs (referred as anchors). The discovery of fixed co-binding patterns can be considered a matrix decomposition (or factorization) problem where an input matrix encodes nucleotides surrounding TFBSs. The ultimate goal is to group nucleotide patterns from this matrix. The non-negative matrix factorization (NMF) technique decomposes a given non-negative matrix into two low-rank, non-negative matrices to reveal underlying patterns and structures within the data (28,29). This technique has been useful in computational biology to reveal molecular patterns from high-throughput data (28,30). seqArchR is the first novel application of NMF for the simultaneous identification of sequence features and corresponding clusters (31). The seqArchR tool identifies critical DNA elements in promoter regions by applying NMF to the corresponding one-hot encoded sequences (31). This approach inspired us to use NMF to address the discovery of TF co-binding patterns.

We present COBIND, a Snakemake-based (32) pipeline for the *de novo* discovery of TF co-binding patterns from input sets of TFBSs (https://bitbucket.org/CBGR/cobind_tool). We applied COBIND to 5699 sets of high-quality TFBSs from seven species (*Arabidopsis thaliana*, *Caenorhabditis elegans*, *Danio rerio*, *Drosophila melanogaster*, *Homo sapiens*, *Mus musculus* and *Rattus norvegicus*) for 401 unique TFs stored in the UniBind database (33). COBIND recovered established and unreported co-binding events between TFs. Among TFs from the same structural family, the majority shared co-binding patterns. In human and mouse genomes, genomic regions harboring the predicted co-binding events are evolutionarily more conserved than genomic regions without co-binding. Moreover, increased chromatin accessibility in matching human cell lines supported the predicted co-binding events from bulk experimental data. Finally, using single-molecule footprinting data from mouse embryonic stem cells, we confirmed that the anchor and co-binding patterns associated with some TFs are significantly enriched for TF co-occupancy at the single-molecule level. Overall, COBIND can *de novo* discover regions in the genomes that harbor patterns of co-binding events between TFs.

## Materials and methods

### Predicting co-binding patterns with COBIND

**Figure 1.**
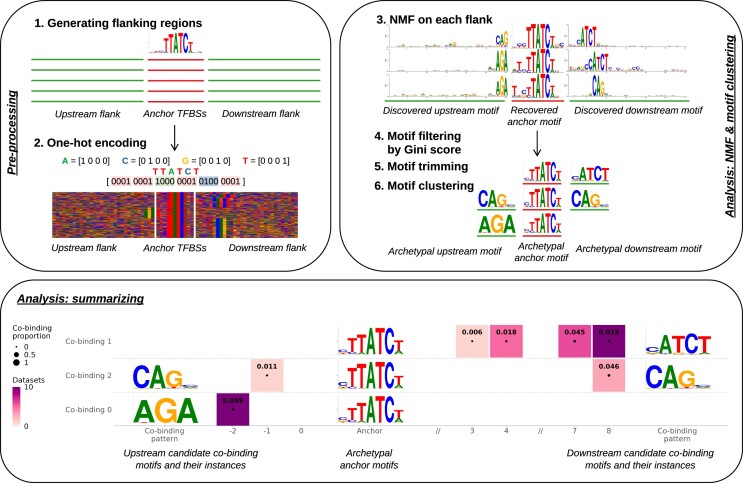
Schematic workflow of COBIND. 1) COBIND takes one or multiple input file(s) that provide regions of interest, such as TFBSs. The tool extracts upstream and downstream DNA sequences (default: 30 nt each). 2) The sequences are one-hot encoded to construct encoded matrices of upstream and downstream sequences. 3) COBIND applies NMF on each matrix to extract DNA patterns. 4) The tool computes the Gini coefficient of each DNA pattern and discards the uninformative patterns. 5) COBIND trims the identified flanking patterns to reveal possible TF co-binding motifs. 6) RSAT matrix-clustering groups redundant motifs, and COBIND constructs archetypal motifs summarizing each cluster of similar motifs. 7) The predicted co-binding patterns are visualized through a heatmap representing the predicted spacing between the anchor (at the center) and the co-binding patterns with the corresponding orientation. The predicted co-binding patterns are provided on the left and/or right of the plots, depending on whether they were found upstream and/or downstream of the anchor sites. The position of the heatmap tiles provides, for each pattern, the number of nucleotides between the co-binding and anchor sites. The color of a heatmap tile represents the number of input datasets in which the co-binding pattern was predicted. The dot size and number within the heatmap tileblocks correspond to the proportion of input sequences in which a co-binding instance with a specific spacing was predicted.

COBIND takes a BED (or FASTA) formatted file providing the genomic coordinates of anchor regions (or DNA sequences centered at the anchor sites), such as TFBS locations, as input. The tool uses NMF to reveal recurring DNA motifs with space constraints in the regions surrounding the input anchors, which are not factorized. The underlying computational pipeline consists of the following steps (Figure 1):

**Step 1: Extraction of flanking regions**. COBIND extracts the DNA sequences surrounding the anchor TFBSs (*n* bp upstream and downstream; *n* = 30 by default) using bedtools (v2.29.2) (34).
**Step 2: One-hot encoding**. The flanking sequences are one-hot encoded as vectors of 4 bits per DNA nucleotide (A: 1000, C: 0100, G: 0010, T: 0001; ambiguous nucleotides $ \notin [ {A,\ C,\ G,\ T} ]$ are encoded as 1111 (Figure 1 Step 2)). COBIND constructs two matrices representing the upstream and downstream sequences by combining the vectors of one-hot encoded sequences. Each row corresponds to a sequence flanking an anchor TFBS from the input set.
**Step 3: Non-negative matrix factorization**. COBIND applies NMF to each matrix using the NMF function of scikit-learn (v0.23.2) (35). The NMF decomposes an input matrix into two matrices: one representing *k* components (or factors) of nucleotide patterns and one informing the presence of the identified patterns in each row of the input matrix (28,30). Importantly, COBIND applies NMF with multiple values of *k* to increase its capacity to capture co-binding patterns with different resolutions (see section ‘Parameter settings’ below) (Figure 1 Step 3). Next, COBIND assigns input sequences to each component following the methodology described by Kim and Park (36). Finally, COBIND builds motifs as positional frequency matrices (PFM) for each component by computing the occurrence frequencies of each nucleotide at each position (Additional file 1: Supplementary Figure S1).
**Step 4: Motifs filtering**. COBIND filters out PFMs with information content (IC) < 2 to focus on informative patterns. Next, it aims to identify motifs corresponding to positions with local enrichment of high IC to distinguish them from motifs with scattered high IC positions or equal distribution of IC along the flanking region (Additional file 1: Supplementary Figure S1). Specifically, COBIND computes the Gini coefficient *g* of each PFM to measure the inequality of IC values (37). We discard PFMs with a low Gini coefficient (*g* < 0.5 by default).
**Step 5: Motif trimming**. COBIND extracts the positions corresponding to the local enrichment of high IC values, revealing co-binding motifs. Specifically, it first computes the smallest window of size *n* that contains at least half of the IC of the complete flank. Finally, COBIND doubles the size of the window (adding *n*/2 nucleotides up and downstream) to define the co-binding motif.
**Step 6: Motif clustering**. COBIND can identify similar motifs multiple times since (i) NMF runs with different values for *k*, (ii) similar motifs can have different spacings with the core motif, and (iii) multiple TFBS sets can be processed in parallel, resulting in the identification of similar motifs (Figure 1 Step 3). COBIND provides non-redundant motifs by clustering the identified motifs (Figure 1 Step 6). Specifically, we integrate the RSAT *matrix-clustering* tool (38) to cluster similar motifs (we provide the used parameters in Additional file 1: Supplementary Table S1). For each motif cluster, COBIND constructs an archetypal motif following the methodology from (39), which summarizes motifs by computing the means of the counts from the aligned matrices in each cluster. We discarded successive flanking positions with IC < 0.1 from the archetypal motif.
Similarly, we cluster and summarize the anchor TFBS sequences associated with each co-binding motif (Additional file 1: Supplementary Table S1).

**Step 7: Summarize the discovered co-binding events**. COBIND computes the relative spacing and orientation to the anchor motif from each co-binding archetypal motif discovered. Finally, it illustrates the corresponding co-binding events as heatmaps (Figure 1 Step 7). The spacing summary plot describes the relationship between the anchor (provided at the center of the plot) and the discovered co-binding patterns (upstream or downstream from the anchor). On the x-axis, one visualizes the number of nucleotides between the anchor and the discovered co-binding pattern motifs, illustrated as sequence logos. The color intensity in the heatmap illustrates the proportion of the input datasets with predicted co-binding motif instances. The inserted number indicates the proportion of unique sequences in the datasets with the corresponding co-binding pattern, orientation, and spacing, that are predicted to harbor the corresponding co-binding configurations.

### Parameter settings

The hyperparameters of COBIND are the length of the flanking regions, the number of components for the different runs of the NMF, and the Gini coefficient threshold. Below, we describe the values used in this study for each parameter and why we selected these values.

**Length of the flanking sequences**. We considered 30 bp upstream and downstream of the input TFBSs to report co-binding events likely associated with physical interactions between the TFs. The longest TFBS motif used in UniBind is about 20 bp-long (33), and cooperative TFs bind TFBSs 9–10 bp away from each other on average (19). Furthermore, larger flanking regions resulted in noisier co-binding motifs and could miss co-binding motif configurations (Additional file 1: Supplementary Figure S2).

**Number of components**. COBIND runs NMF several times with different numbers of components (*k*). We used synthetic data to estimate a suitable range of values for *k*. Specifically, we implanted different proportions of instances of predefined motifs in random sequences. We considered sets of 800, 1000, 4000, 8000, 10 000, 40 000 and 80 000 synthetic sequences. For each set of synthetic sequences, we injected an instance of the considered motif in 0.5, 1, 3, 5 and 10% of the sequences. We ran COBIND with *k* ∈ [2, 17] for each synthetic dataset. For each *k* value, we evaluated specificity and sensitivity by computing the F1 score and Matthew's correlation coefficient (MCC) for each discovered motif. Moreover, we reported the proportion of correctly predicted motifs. We considered that COBIND predicted the correct motif if it was similar to the injected motif—as assessed by Tomtom (40) with a *P*-value <0.05 (Additional file 1: Supplementary Table S1). We used the results from the synthetic data to select *k* ∈ [3, 6] for further analyses (Additional file 1: Supplementary Figures S3–S15).

**Gini coefficient threshold**. To empirically determine the Gini coefficient threshold, we compared the distributions of Gini coefficients for the motifs predicted by COBIND on the TFBS datasets from UniBind (see below) with those of predictions from random sequences. For each UniBind set of TFBSs, we constructed a set of synthetic sequences by shuffling the DNA sequences flanking the TFBSs with the *kmer shuffling* module of the *BiasAway* tool with *k* = 1 (41). For each species considered, we selected the Gini coefficient threshold corresponding to a 1% false discovery rate (Additional file 1: Supplementary Figure S16). This strategy resulted in the following Gini coefficient thresholds: 0.50 for *Arabidopsis thaliana*, 0.46 for *Caenorhabditis elegans*, 0.48 for *Danio rerio*, 0.49 for *Drosophila melanogaster*, 0.50 for *Homo sapiens*, 0.54 for *Mus musculus* and 0.53 for *Rattus norvegicus*. Finally, we estimated the stability of the thresholds obtained with this strategy by performing the same analysis multiple times and subsampling the number of datasets from *H. sapiens* and *M. musculus*. The Gini coefficient thresholds were stable across the different runs (Additional file 1: Supplementary Figure S17).

### Transcription factor binding site datasets

We used the robust collection of TFBS sets from the UniBind database (2021 version) (https://unibind.uio.no/) derived from 7 species (*Arabidopsis thaliana*, *Caenorhabditis elegans*, *Danio rerio*, *Drosophila melanogaster*, *Homo sapiens*, *Mus musculus, Rattus norvegicus*) (33). UniBind TFBS datasets derive from pairs of ChIP-seq peak datasets and JASPAR TF binding profiles. When multiple TF binding profiles exist for a given TF, we selected, for each corresponding ChIP-seq dataset, the profile providing the best centrality *P*-value as assessed in UniBind. We did not consider JASPAR TF binding profiles associated with TF dimers. Datasets with <1000 TFBSs were filtered out (Additional file 1: Supplementary Table S2).

### Benchmarking

#### Synthetic datasets

We generated the synthetic datasets following the methodology mentioned above. We constructed synthetic datasets with 800, 1000, 4000, 8000, 10 000, 40 000 and 80 000 sequences with instances of a known motif inserted in 0.5, 1, 3, 5 and 10% of the sequences. We considered 13 motifs (3- to 19-bp long) from the JASPAR database (7), which were synthetically inserted into random sequences with varying spacing (6- to 22-bp) from the anchor sites. To run SpaMO, we inserted the CTCF (MA0139.1) motif at the center of the flanking sequences since SpaMO requires a motif to anchor its search for co-binding patterns.

#### Comparisons between COBIND and other tools

We used the synthetic data to compare COBIND with STREME (42), RSAT peak-motifs *dyad-analysis* (25), RSAT peak-motifs *oligo-analysis* (26,43,44), RSAT peak-motifs *position-analysis* (43,44) and SpaMo (21). We compared the motifs predicted by the different tools with the original inserted motifs using Tomtom (40) (Additional file 1: Supplementary Table S1). We considered a discovered motif as a match to the injected motif if Tomtom predicted them to be similar with a *P*-value <0.05. We provide the number of incorrectly predicted motifs. To run SpaMo, we used the JASPAR 2020 CORE collection of non-redundant profiles (45). We computed the F1 score and Matthew's correlation coefficient (MCC) to assess the capacity of the tools to retrieve the correct sequences where the motifs were injected. When the correct motif was predicted multiple times, we reported the one providing the highest *F*1 score.

STREME and RSAT algorithms were run to discover a maximum of 18 motifs to match the number of possible motifs to be predicted by COBIND. All tools were run with default parameters otherwise. When estimating the running time, we used the parallelizable version of the NMF for COBIND.

### Inference of the co-binding transcription factors from the discovered co-binding motifs

We assessed the similarities between a predicted co-binding motif and known motifs associated with 5272 TFs—collected from JASPAR 2022 non-redundant CORE, JASPAR 2022 non-redundant Unvalidated taxon-specific, and CIS-BP (5)—using Tomtom (Additional file 1: Supplementary Table S1) (40). We predicted TF_B_ to bind the predicted co-binding motif associated with anchor TFBSs of TF_A_ if Tomtom reported a similarity *P*-value <0.05 with the known canonical motif bound by TF_B_ (criteria I). To assess possible physical interactions between TF_A_ and TF_B_, we retrieved protein-protein interaction (PPI) data from STRING v11.0 (46). We considered TF_A_ and TF_B_ to interact physically when their STRING PPI score was above or equal to 500 (criteria II). We reported the co-binding pair TF_A_-TF_B_ to be already ‘known’ when they met criteria I and II. We reported TF_A_–TF_B_ as a novel co-binding pair when they met criteria I but not II.

### Shared co-binding motifs across transcription factor structural families

To assess the conservation of co-binding motifs across TFs, we ran COBIND (up to Step 5) on all UniBind datasets. We independently clustered all the predictions (COBIND Step 6) for each TF structural family; note that this step considered TFs from multiple species. TFs without known structural families were excluded from this analysis. When the co-binding motifs associated with two (or more) TFs from the same family were similar (i.e. belonged to the same cluster), we considered the TFs to share the same co-binding motif.

### Evolutionary conservation

We downloaded the following conservation tracks in bigwig format from the UCSC genome browser: phastCons100way (human; hg38), phastCons60way (mouse; mm10), phastCons135way (*C. elegans*; WBcel235), and phastCons27way (*D. melanogaster*; dm6) (47). We obtained the 63 flowering plants PhastCons conservation track from PlantRegMap (48) for analyzing datasets from *A. thaliana*. We computed the conservation scores of a genomic region using the *aggregate* and *extract* functions of bwtool (v1.0) (49).

We compared the distributions of conservation scores between two regions with one-sided Wilcoxon signed-rank tests. Specifically, we compared the conservation values at the anchor (or co-binding) sites between the genomic regions predicted to harbor a co-binding pattern or not. These comparisons result in two *P*-values per co-binding pattern - one comparing the anchor sites and one comparing the co-binding sites. Histograms were used to summarize the *P*-values across datasets, considering the co-binding pattern with the lowest *P*-value per dataset. We compared the *P*-values to random expectation. Specifically, we randomly assigned, for each dataset, each genomic region to harbor or not a co-binding pattern (keeping the same number of regions in each category as the number predicted by COBIND). We performed five randomizations per dataset and reported the combined results.

### DNase I hypersensitive footprinting analysis

We downloaded DNase I hypersensitivity (DHS) data generated by the ENCODE consortium for multiple human cell types in bigwig format at https://resources.altius.org/∼jvierstra/projects/footprinting.2020/ (50). We considered the 53 cell lines or tissues for which both DHS and COBIND predictions were available; we analyzed 66 DHS datasets associated with 70 co-binding configurations for 44 TF binding profiles as anchors. We followed the methodology described above to compare evolutionary conservation scores when comparing the DHS signal between two genomic regions (with or without a co-binding event predicted by COBIND).

### Single-molecule footprinting analysis

We downloaded 12 double enzyme single-molecule footprinting (SMF) datasets produced from triple DNA methyltransferases knockout lines of mouse embryonic stem cells (mESCs) from ArrayExpress with accession numbers E-MTAB-9033 and E-MTAB-9123 (51). Whenever applicable, we performed the analyses of SMF data with the SingleMoleculeFootprinting R package following the instructions previously described (52). To compare the SMF data in the genomic regions predicted by COBIND to contain or not a co-binding event in mESC datasets, we modified the SortReadsByTFCluster_MultiSiteWrapper functions from the development version of the SingleMoleculeFootprinting package (53) (https://bitbucket.org/CBGR/cobind_manuscript/src/master/bin/single_molecule/analyse_sm/sort_tf_reads_by_cobind_clusters.R). Using the methylation status provided by the SMF data, we determined each genomic region to be either (i) free of nucleosomes (‘Accessible’), (ii) occupied by nucleosomes (‘Nucleosome’), (iii) bound only by the anchor (‘Anchor’), (iv) bound only by the co-binding TF (‘Co-binding’) or (v) bound by both the anchor and co-binding TF (‘Anchor + Co-binding’). We summed the number of molecules for each state in different samples and computed the proportions of molecules. We summarized the proportions of each state for all predictions and all anchors together. We compared the proportions of each state in genomic regions predicted to contain a co-binding event or not using a two-sided Wilcoxon signed-rank test. We compared the same type of regions in the ‘Anchor + Co-binding’ state for individual anchors using a one-sided Wilcoxon signed-rank test.

## Results

### COBIND discovers co-binding patterns *de novo*

#### Comparisons with other tools on simulated data

We report a new computational framework, COBIND, to predict space-fixed co-binding patterns in the vicinity of TFBSs provided as input. COBIND relies on applying NMF to the one-hot encoded regions flanking the user-provided TFBSs to predict co-occurring DNA motifs with fixed spacing (Materials and methods). We compared COBIND to other tools discovering motifs *de novo* (STREME, RSAT *dyad-analysis*, RSAT *oligo-analysis* and RSAT *position-analysis*). Additionally, we compared COBIND to SpaMO, which predicts spatially co-occurring instances of known motifs (Additional file 1: Supplementary Table S3). For comparisons, we generated synthetic data by injecting instances of known motifs (13 distinct motifs were used) at different frequencies in random sets of 800 to 80 000 DNA sequences (Materials and methods).

**Figure 2.**
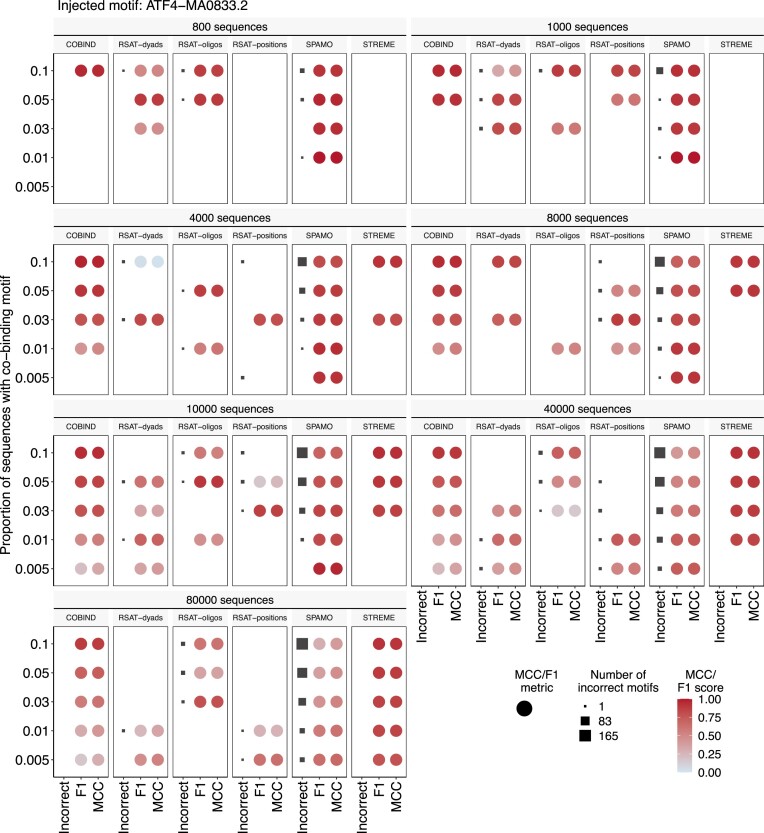
Comparisons between COBIND and other tools on simulated data. We ran COBIND, STREME, RSAT-dyads, RSAT-oligos, RSAT-positions and SPAMO (subfacets) on simulated data containing different numbers of sequences (from 800 to 80 000, see facets) with inserted JASPAR MA0833.2 TF binding motif instances for ATF4 (see Methods for details). We considered (i) *F*1 scores and Matthew's correlation coefficient (MCC) (in case multiple correct motifs are found, a motif with the highest *F*1 score is visualized). (ii) the number of incorrect motifs discovered in each experiment. Good performance of a tool would be indicated by a discovered motif with a high *F*1 score and high MCC and no incorrectly discovered motifs.

COBIND predicted the correct motif inserted when considering a minimum of 800 sequences for 10 of the 13 used inserted motifs. For example, COBIND accurately predicted the injected ATF4 motif in datasets of varying sizes without incorrect motif predictions (Figure 2). When considering the shorter TAL1 motif (3-bp known to be co-bound by the dimer GATA:TAL1), COBIND provides the most accurate predictions in multiple datasets when other tools obtain lower *F*1 scores and MCC values and predict incorrect motifs (Additional file 1: Supplementary Figure S18-S29). Overall, COBIND predicted no false positive motif across most configurations (see the number of incorrect motifs discovered in Additional file 1: Supplementary Figure S18-S29). In most cases, COBIND discovered the injected motifs in the correct sequences, as illustrated by high *F*1 scores and MCC values. Comparably, STREME discovered only correct motifs but failed to predict any motifs in some configurations (especially when the number of sequences with the injected motif was low). When considering many sequences (datasets of 40 000 and 80 000 sequences), COBIND and STREME recovered the correct motifs. SpaMo discovered the correct inserted motif in most cases, but it came at the cost of predicting many false positives. One should note that SpaMo requires a reference set of known motifs to predict co-occurrences, while COBIND predicts co-binding motifs *de novo*.

COBIND exhibited a similar run time to the other tools on datasets with up to 10 000 sequences. Nevertheless, it was slower than the other tools, except STREME, with larger datasets (>40 000 sequences) due to the motif clustering step applied to the results of the NMF with 3–6 components. Notwithstanding, the COBIND workflow (from motif discovery with NMF to co-binding summary) takes ∼500 s on 40 000 sequences as it allows for parallelization across CPU cores (Additional file 1: Supplementary Figure S30).

#### Proof-of-concept: POU5F1 co-binding with SOX2 or SOX17

**Figure 3.**
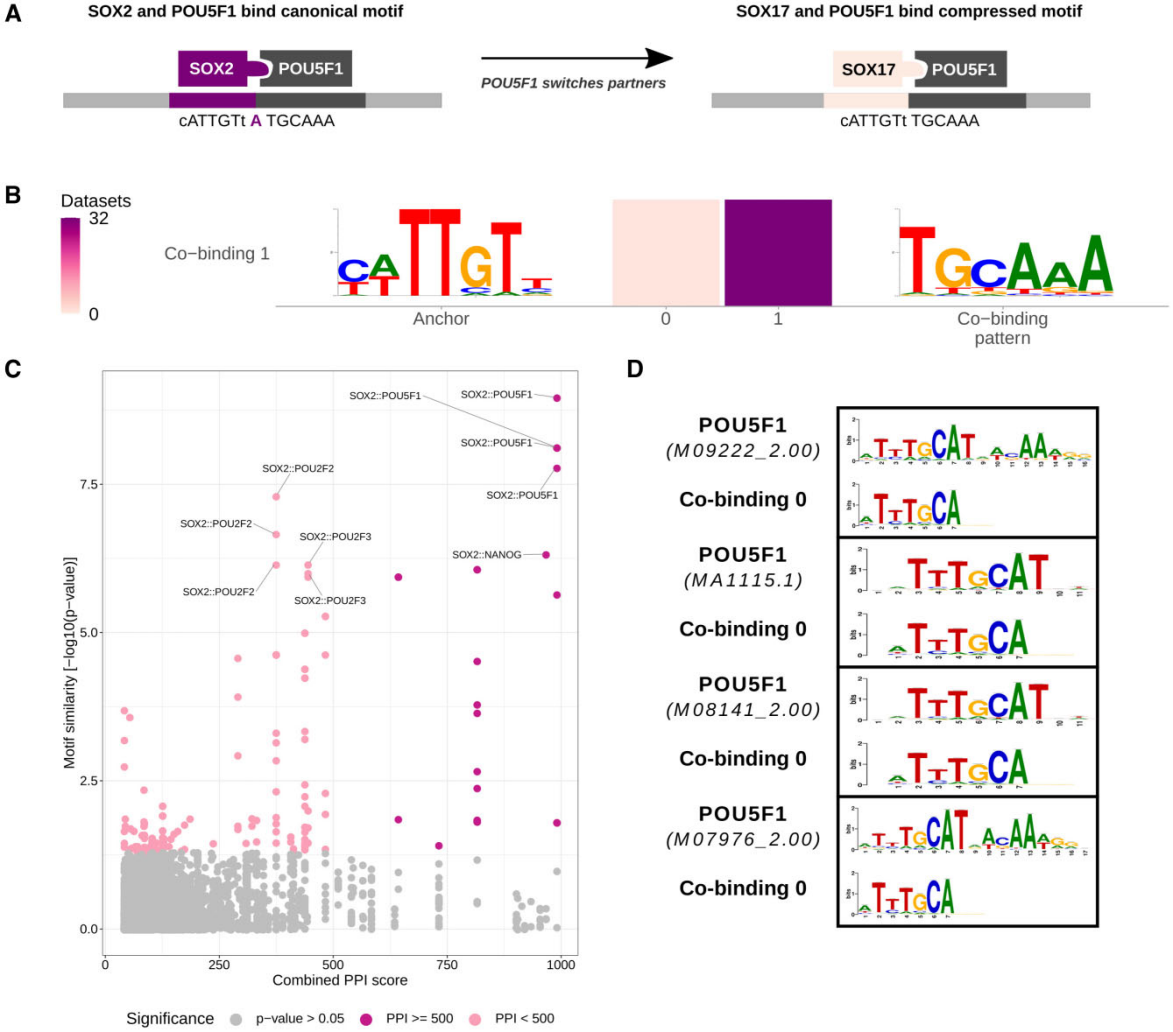
Application of COBIND to SOX2 and SOX17 TFBS datasets. COBIND predicts POU5F1 to co-bind with SOX2 (human and mouse) and SOX17 (mouse), upholding the previously known mechanism of POU5F1 switching partner TFs and binding motifs with different syntax. (**A**) POU5F1 partners with SOX2 to bind its canonical motif. In contrast, POU5F1 associates with SOX17 to bind a compressed motif. (**B**) The co-binding patterns discovered in SOX2 and SOX17 datasets correspond to the expected canonical and compressed motifs. One co-binding pattern was discovered downstream of the anchor with either zero or one nucleotide between them. (**C**) The co-binding motif found in the SOX2 datasets was associated with POU5F1 using motif similarity and PPI data (significant motif similarity *P*-value < 0.05 and PPI combined score > 500). (**D**) Visual representation of the similarity between the discovered co-binding motif and motifs recognized by POU5F1 in JASPAR and CIS-BP.

As a proof-of-concept, we applied COBIND to UniBind TFBS datasets for the SOX2 and SOX17 TFs. Previous studies established that both TFs partner with POU5F1 in pluripotent cells, where POU5F1 co-binds with SOX2 at regulatory elements to promote pluripotency. At the same time, it co-binds with SOX17 to control endoderm differentiation at other regulatory elements (11,16). While POU5F1 and SOX2 cooperate through binding at instances of their respective canonical motifs, POU5F1 can associate with SOX17 to bind a compressed motif (Figure 3A). COBIND successfully discovered the two co-binding patterns - canonical and compressed - recognized by POU5F1 when applied to the SOX2 and SOX17 TFBS datasets from *H. sapiens* and *M. musculus* (Figure 3B). COBIND retrieved the correct pattern in 11 human SOX2 datasets (58%), with ∼7% of the sequences predicted to harbor the pattern, and in 20 mouse datasets (27%), with ∼6% of the sequences harboring the pattern (Additional file 1: Supplementary Figure S31A, B). In agreement with previous studies, the datasets where COBIND predicted the pattern derived from human and mouse ESC and mouse embryonic fibroblast (16,18,54). When considering the SOX17 datasets, COBIND successfully discovered the canonical co-binding pattern and the compressed motif. It predicted the compressed co-binding pattern in two datasets (50%; derived from mouse ESC), with 5% of the sequences harboring the co-binding motif (Additional file 1: Supplementary Figure S31C).

In this proof-of-concept example, we knew *a priori* that POU5F1 was the binding partner of SOX2 and SOX17. Nevertheless, COBIND discovered *de novo* the co-binding motif. In other settings, one would not know the TF binding to the co-binding motif discovered. To address this challenge, we combined motif similarity to already known motifs from JASPAR and CIS-BP with protein-protein interaction (PPI) data from the STRING database to infer the TFs potentially binding the motifs revealed by COBIND (Materials and methods). This strategy confirmed that the framework inferred POU5F1 as the binding partner of SOX2 and SOX17 (Figure 3C, D; Additional file 1: Supplementary Figure S31).

#### COBIND discovers known and novel co-binding patterns in a large-scale analysis across several species

**Figure 4.**
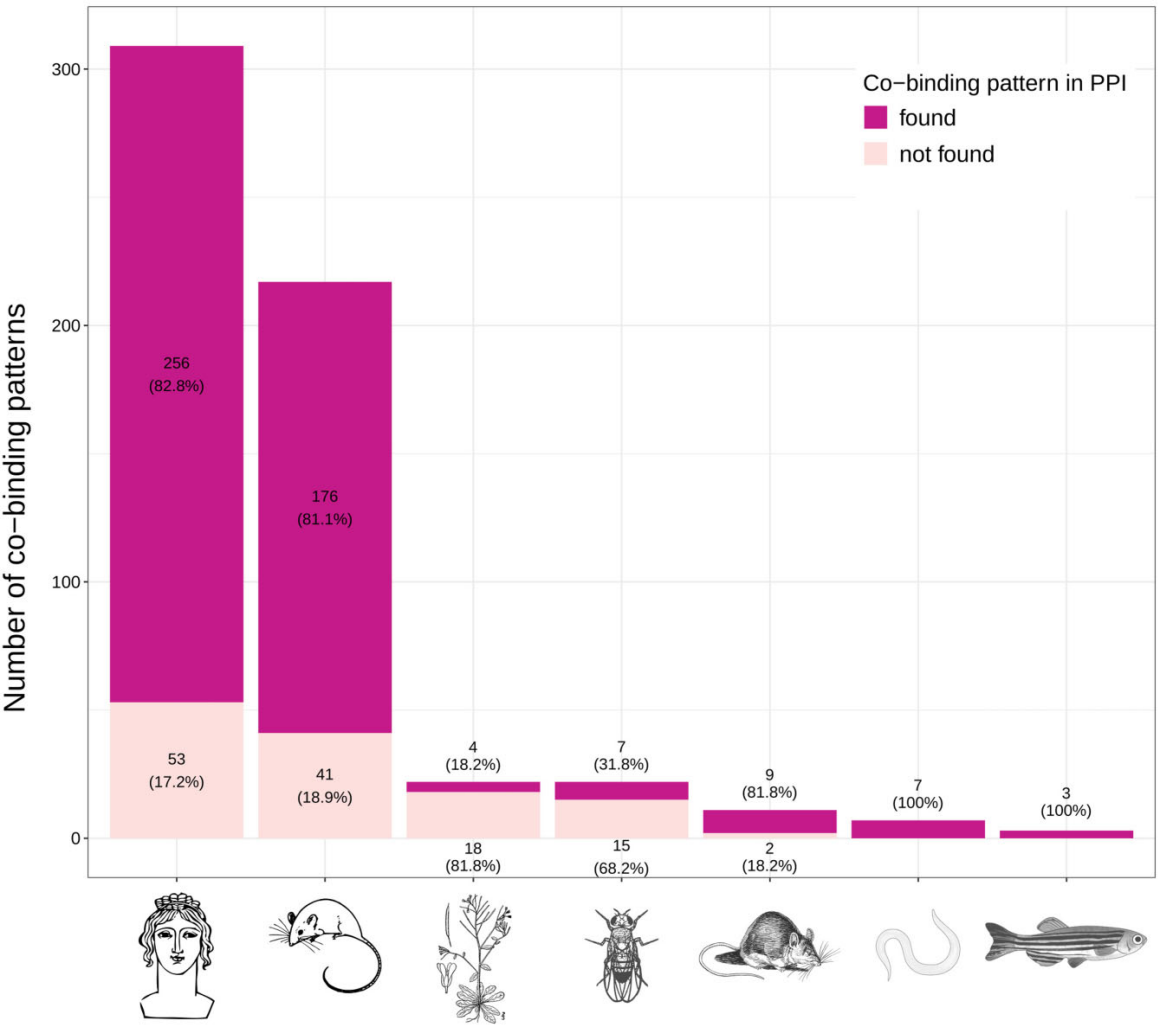
Overview of COBIND predicted co-binding patterns. The bar plot provides the number of co-binding patterns (y-axis) discovered by COBIND with (dark pink) or without (light pink) support from motif similarity and PPI data. The bars contain the corresponding numbers and percentages. Each bar summarizes the results for a species (x-axis).

We ran COBIND on 5699 UniBind TFBS datasets associated with 401 unique TFs from seven species (Materials and methods; Additional file 1: Supplementary Table S2). Beyond predicting co-binding patterns with COBIND, we inferred the TFs likely binding to the discovered patterns following the strategy described above (also see Materials and Methods). Altogether, COBIND revealed 591 co-binding patterns for 224 unique TFs (22 in *A. thaliana* datasets, 7 in *C. elegans*, 3 in *D. rerio*, 22 in *D. melanogaster*, 309 in *H. sapiens*, 217 in *M. musculus* and 11 in *R. norvegicus*). For 78% of the reported co-binding motifs (462 out of 591), we found motif similarity and PPI data to support the inferred pair of co-binding TFs (Figure 4). Specifically, the data supported all co-binding patterns for *D. rerio* and *C. elegans*, more than half of the patterns for *R. norvegicus*, *H. sapiens* and *M. musculus*, 18.2% for *A. thaliana* and 31.8% for *D. melanogaster*. We provide all predictions for the community to explore through a dedicated website at https://cbgr.bitbucket.io/COBIND_results_page/.

Next, we evaluated the frequency at which a TF_A_ is predicted as a partner of TF_B_ and, conversely, TF_B_ as a partner of TF_A_. We analyzed co-binding patterns predicted in a specific cell type for each TF_A_, provided data was available for a predicted co-binding TF_B_ in the same cell type (noting that multiple TFs can recognize the same co-binding pattern). When examining datasets linked to TF_B_, COBIND successfully predicted back TF_A_ in 77% of the co-binding patterns from human datasets and 84% from mouse datasets.

Furthermore, the analysis of motif similarity between COBIND’s predictions revealed that 67% (143 out of 214) of the TFs shared a co-binding motif with another member of the same TF structural family (Materials and Methods), which confirmed the conservation of co-binding patterns within families of TFs across species. It is noteworthy that anchor regions bound by TFs from the same structural family tend to overlap (Mann–Whitney U test *P*-value < 0.05) with those bound by different TF families, even though they typically show low Jaccard index values (Additional file 1: Supplementary Figure S32). This observation was consistent across different species, covering all regions with and without predicted co-binding patterns (Additional file 1: Supplementary Figure S32). However, no statistically significant differences were noted (Mann–Whitney *U* test *P*-value > 0.05) when comparing regions with predicted co-binding patterns to those without, irrespective of whether the same or different TF family members bind them.

As a case example, COBIND predicted two co-binding patterns around TEAD1 TFBSs. Specifically, COBIND revealed the same motif with different spacings from the provided anchor TFBSs (2nt upstream or 3nt downstream of TEAD1 TFBSs; Additional file 1: Supplementary Figure S33A). We found that the two co-binding patterns occurred in ∼6% (3-bp downstream pattern) and ∼7% (2-bp upstream pattern) of the input sequences from mouse nerve tumor cells and in ∼3% (2-bp upstream and 3-bp downstream patterns each) of the sequences from human pancreas and astrocytoma cells. Our approach inferred that TEAD2 and TEAD4 cooperate with TEAD1 (Additional file 1: Supplementary Figure S33B, C). Previous studies reported that TEAD TFs bind either an isolated M-CAT element or direct DNA repeats with spacing ranging from 0 to 6 bp (55–57). These studies further support the co-binding patterns predicted by COBIND. The predictions around TEAD1 TFBSs provide an example of COBIND’s capacity to predict co-binding events supported by motif similarity and PPI data.

To exemplify predictions of new co-binding TFs, COBIND revealed a co-binding motif located 4 bp upstream of HY5 anchor TFBSs in *A. thaliana* (Additional file 1: Supplementary Figure S34A). The analyses of TFBS datasets associated with the TFs ABF1, ABF3, ABF4, GBF2 and GBF3 displayed the same co-binding pattern. We found that the canonical motifs recognized by human NF-Y TFs (NF-YC and NF-YA) were similar to the co-binding motif discovered by COBIND (Additional file 1: Supplementary Figure S34B, C). However, no PPI data currently support the physical interactions between the anchor TFs and NF-Y orthologs in plants. Nevertheless, the bZIP67 TF, which belongs to the same basic leucine zipper (bZIP) family as HY5, ABF1, ABF3, ABF4, GBF2 and GBF3, is known to form a transcriptional complex together with NF-YC2 and bind ER stress response elements (ERSEs) to regulate omega-3 fatty acid content in *A. thaliana* (58). In agreement with this observation, we noted that the anchor motif combined with the co-binding motif discovered by COBIND forms the motif associated with ERSEs (59). Furthermore, HY5 competes with bZIP28, another member of the bZIP family, to bind ERSEs on promoters of unfolded protein response genes, and bZIP28 interacts with NF-Y protein complexes (59,60). Consistent with this knowledge, we found that most of the genomic regions predicted by COBIND to harbor the co-binding events in the HY5 dataset were in promoter regions (Additional file 1: Supplementary Figure S34D). Altogether, these multiple lines of evidence strongly support the co-binding patterns predicted by COBIND between bZIP and NF-Y TFs in *A. thaliana*.

### COBIND discovers the extended motif bound by CTCF

**Figure 5.**
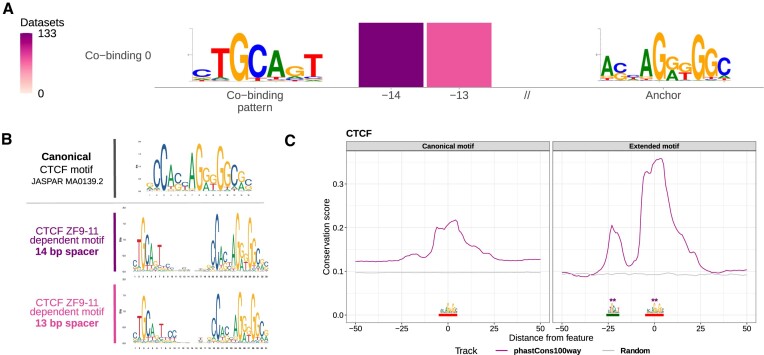
CTCF binds a conserved extended motif. (**A**) COBIND predicted two binding configurations through an extended motif for CTCF in human and mouse datasets. The extended binding pattern was discovered upstream of the anchor with either thirteen or fourteen nucleotides between them. (**B**) The two binding configurations derive from contacts between the zinc fingers (ZF) 9–11 and the DNA at the upstream motif. (**C**) Comparison between vertebrate evolutionary conservation of *H. sapiens* genomic regions harbouring the CTCF canonical motif only (left) and regions with the extended motif revealed higher conservation of the extended motif. The purple lines provide the mean conservation score across the regions considered. The grey lines provide the mean conservation score across the same number of random genomic regions in the human genome. ** indicates a Wilcoxon test *P*-value < 0.001 at the anchor (red) and motif sites (green).

We investigated another example where COBIND predicted a co-binding pattern unsupported by motif similarity and PPI data. Specifically, COBIND identified a co-binding motif in the proximity of CTCF TFBSs for several datasets (Figure 5A). We observed two spacing configurations between the anchor CTCF TFBSs and the predicted co-binding events. Across the datasets, COBIND predicted from 2.6 to 5% of the sequences to harbor the co-binding patterns. All datasets from human and zebrafish and 25% of the mouse datasets (95 out of 382), which cover a large spectrum of cell types (Additional file 1: Supplementary Table S4), contained the identified co-binding patterns (Additional file 1: Supplementary Figure S35). Previous studies have reported the motif predicted by COBIND as an extension of the canonical CTCF motif (61,62). These studies revealed that the eighth zinc finger of CTCF acts as a linker instead of a clamp to allow zinc fingers 9–11 to bind the extended motif (Figure 5B), affecting the binding efficiency, residence time, and binding off-rate of CTCF (61,62). Consequently, COBIND did not predict co-binding between distinct TFs, but CTCF seldomly bound an extended motif through different combinations of zinc finger contacts with the DNA.

Since evolutionary conservation is a hallmark of functional importance (47), we assessed the functional relevance of the genomic regions harboring the discovered binding patterns by examining their evolutionary conservation across vertebrates (Materials and Methods). We observed that the anchor and extended motif sites (Figure 5C, right, red and green segments, respectively) were more conserved in the regions predicted to harbor the extended motif than in the other regions (Wilcoxon test *P*-value < 0.001 for both segments; Figure 5C, right versus left). The increased evolutionary conservation was consistent across all spacing configurations observed in human and mouse. These results exemplify how COBIND can predict relevant DNA patterns that do not correspond to the co-binding of two proteins but to binding variants for the same TF, a particular case of the zinc finger families.

### Genomic regions harboring a co-binding pattern are evolutionarily more conserved than regions without co-binding

**Figure 6.**
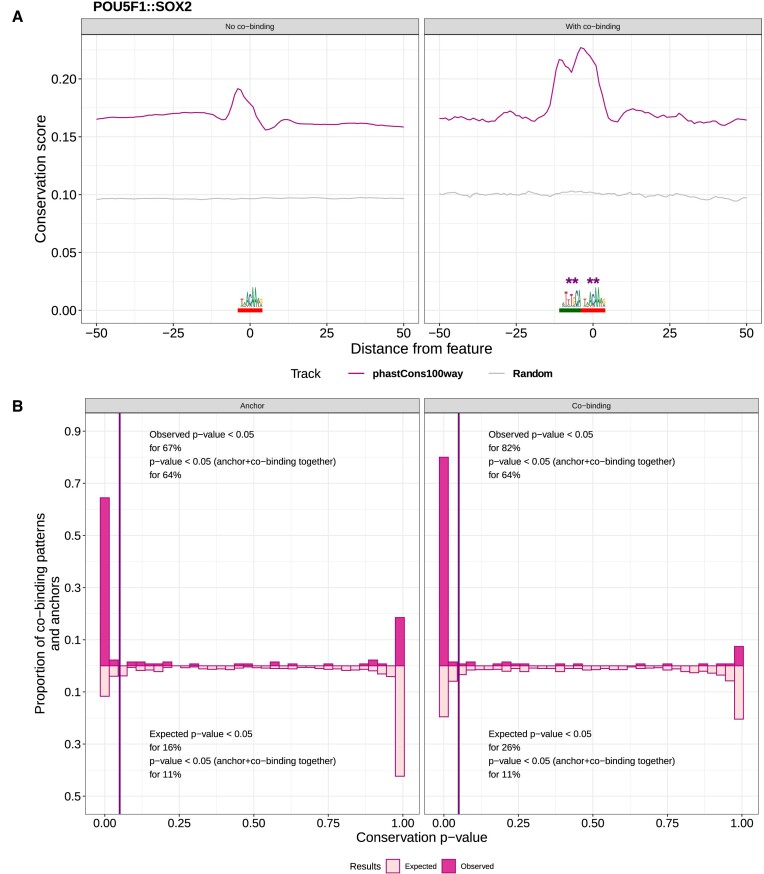
Analysis of the evolutionary conservation of co-binding patterns discovered by COBIND in the human genome. (**A**) Comparison between vertebrate evolutionary conservation of genomic regions where COBIND predicted a co-binding pattern (right) or not (left). The purple lines provide the mean conservation score across the regions considered. The grey lines provide the mean conservation score across the same number of random genomic regions in the human genome. ** indicates a Wilcoxon test *P*-value < 0.001. The anchor SOX2 anchor motif is underlined in red, and the co-binding motif is underlined in green. (**B**) We compared the evolutionary conservation scores at the anchors' motif locations in genomic regions harboring a co-binding pattern or not. The left panel represents the histogram of the proportion of datasets with the anchor sites harboring higher evolutionary conservation in co-bound regions than in other regions. The right panel represents the corresponding histogram when comparing genomic locations of the co-binding motif. Dark pink represents observed values, and light pink the expected values computed from random assignment of genomic regions predicted as co-bound or not.

We assessed the functional relevance of all the co-binding patterns discovered by COBIND across species by analyzing their evolutionary conservation (Materials and methods). Specifically, we compared the evolutionary conservation of the anchor and co-binding positions (Figure 5C, red and green, respectively) in genomic regions harboring a co-binding pattern, named co-bound regions for simplicity, to those without a predicted co-binding pattern. We first describe the analyses of the case studies presented above as examples. We found that the co-bound regions associated with SOX2::POU5F1 and TEAD1::TEAD in the human and mouse genomes exhibited a significantly increased evolutionary conservation compared to those without predicted co-binding (Wilcoxon test *P*-value < 0.001; Figure 6A; Additional file 1: Supplementary Figure S36A). Notably, we observed increased conservation at the co-binding and the anchor motifs. When considering the co-binding pattern associated with HY5 in *A. thaliana*, we did not observe a significant difference in conservation between the regions with and without the co-binding pattern. Nevertheless, both the anchor and the co-binding motifs exhibited peaks of evolutionary conservation, confirming the likely functional relevance of the predicted co-binding pattern across the flowering plant kingdom (Additional file 1: Supplementary Figure S37A).

Across all datasets and species, we systematically compared the evolutionary conservation at locations of the anchor and predicted co-binding sites discovered by COBIND in co-bound regions versus regions without co-binding sites. Altogether, we observed across species that 23% (for *A. thaliana*), 55% (for *D. melanogaster*), 60% (for *C. elegans*), 82% (for *H. Sapiens*), and 83% (for *M. musculus*) of the datasets with predicted co-binding patterns exhibited a co-binding motif with significantly more conservation in co-bound regions than in the other regions (Figure 6B; Additional file 1: Supplementary Figure S36B-S38). Furthermore, the associated anchor sites were more conserved in the co-bound regions for 31% (*A. thaliana*), 55% (for *D. melanogaster*), 60% (for *C. elegans*), 64% (for *M. musculus*), and 67% (*H. sapiens*) of the datasets (Figure 6B; Additional file 1: Supplementary Figure S36B, Supplementary Figure S37B, Supplementary Figure S38). Finally, we observed for 64% of the human datasets that the locations of both the anchor and a co-binding motif were more conserved in the co-bound regions than in regions without co-binding sites (61% for *M. musculus*, 60% for *C. elegans*, 45% for *D. melanogaster*, and 23% for *A. thaliana*). We compared with random expectation to further support the higher conservation of the anchor and co-binding sites in co-bound regions than similar locations in regions without a predicted co-binding pattern. Specifically, we randomly assigned, for each dataset, each genomic region to harbor or not a co-binding pattern (keeping the same number of regions in each category as the number predicted by COBIND). Figure 6B and Additional file 1: Supplementary Figures S36B and S37B confirm that more datasets exhibit increased conservation in co-bound regions than expected by chance. The consistent increased evolutionary conservation of the co-binding patterns supports the functional importance of COBIND’s predictions across species. Furthermore, our results suggest that the underlying genomic regions harboring a fixed binding motif syntax are evolutionarily important.

### DNase I hypersensitive footprints support the predicted co-binding patterns

We further assessed the co-binding patterns discovered by COBIND by analyzing orthogonal experimental data probing chromatin openness. The DNase-seq assay captures open chromatin regions by revealing DNase I hypersensitive sites (DHS) (63). Importantly, TFs interacting with the DNA in open chromatin regions protect their TFBSs from the DNase cleavage, which leaves a footprint on the corresponding TFBSs (50,64). We retrieved 66 DHS footprint datasets from 53 human cell types (50) that matched some of the UniBind TFBS datasets used in this study. This data allowed us to investigate DHS footprints at the discovered co-binding motifs for 70 co-binding patterns associated with 44 TF binding profiles as anchors. For each co-binding pattern, we compared the depth of the DHS footprint at the locations of the co-binding motifs between the co-bound regions and the other regions (Materials and methods).

**Figure 7.**
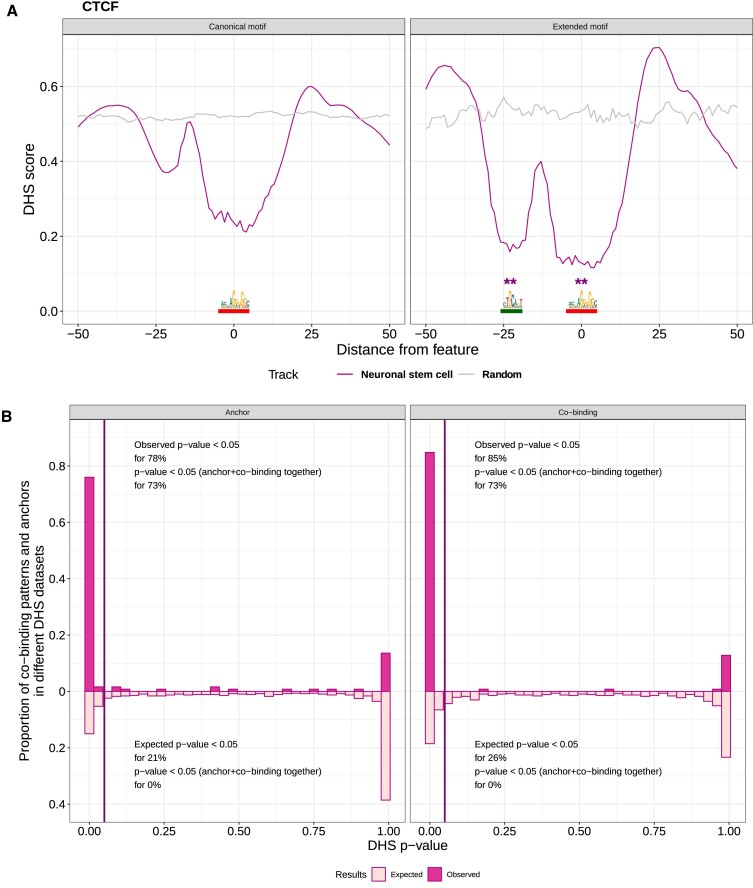
DHS footprinting analyses at anchor and co-binding locations. (**A**) The plots represent the average DHS score in regions surrounding CTCF TFBSs in neuronal stem cells (purple lines) or at random regions (grey lines). The left plot provides DHS scores at genomic regions where COBIND did not predict the CTCF extended motif; the right plot provides the DHS scores at genomic regions harbouring the CTCF extended motif. ** indicates a Wilcoxon test *P*-value < 0.001. The anchor CTCF motif is underlined in red and the upstream portion of the extended motif is in green. (**B**) We compared DHS scores at the anchor motif locations in genomic regions harbouring a co-binding pattern or not. The left panel represents the histogram of the datasets where the DHS footprint was deeper in genomic regions harbouring a co-binding pattern. The right panel represents the corresponding histogram when comparing genomic locations of the co-binding motif locations. Dark pink represents observed values, and light pink the expectation computed from randomly assigning regions as containing or not a co-binding pattern.

As we ran COBIND on regions surrounding TFBSs predicted as high-quality direct TF–DNA interactions with ChIP-seq and computational evidence from UniBind, we expected DHS footprints at the anchor TFBSs. Indeed, we observed footprints of TF-DNA interactions; for instance, the DHS footprints observed for the CTCF datasets (Figure 7A). Notably, the DHS footprints at the CTCF anchor locations were significantly deeper in regions predicted with the extended motif than in the other regions (Figure 7A, red segments). Furthermore, the analyses exhibited deep DHS footprints at the location of the co-binding motifs (Figure 7A, green segment on the right compared to the equivalent locations on the left panel). Overall, we found significantly deeper DHS footprints at the locations of a co-binding motif in the predicted co-bound regions than in the other regions for 85% of the co-binding pattern - DHS dataset pairs. In comparison, 26% was expected by chance when randomly assigned the genomic regions as co-bound or not (Figure 7B, right panel). The anchor TFBSs at co-bound regions exhibited deeper DHS footprints than the TFBSs in other regions for 78% of the pairs, while 21% were expected by chance (Figure 7B, left panel). When considering deeper DHS footprints at both the anchor and a co-binding pattern sites, it was observed for 73% of the pairs, while it was never observed with the random assignments (Figure 7B). As CTCF datasets represented 43% of the total datasets analyzed here, we performed the same analysis, excluding the CTCF datasets. We found that 53% (25% expected by chance) of the co-binding pattern-DHS dataset pairs showed significant DHS footprints at the anchor TFBSs, and 68% (23% expected by chance) exhibited significantly deeper DHS footprints at the co-binding motif locations in co-bound regions than in the other regions. Altogether, 45% of the pairs exhibited deeper DHS footprints at both the co-bound and anchor TFBS locations. Overall, the DHS footprint analyses supported the co-occupancy of the anchor and the co-binding motifs at the co-bound regions.

### Single-molecule footprints support co-occupancy at single-molecule resolution for some TFs

The DHS footprinting analysis described above relied on bulk DNase-seq data. Consequently, the results provided average estimations of the co-occupancy of TFs across cells. We aimed to investigate the co-occupancy of TFs at the co-binding patterns predicted by COBIND at single-molecule resolution. To this end, we considered single-molecule footprinting (SMF) data, which probed the co-occupancy of TFs and nucleosomes at single-molecule resolution for accessible genomic regions in mouse embryonic stem cells (mESC) (51). The SMF data overlapped 1694 regions with predicted co-binding patterns (483 453 reads) and 54 727 regions (14 457 475 reads) not predicted to contain any co-binding pattern for 17 TFs in mESC (Materials and Methods).

**Figure 8.**
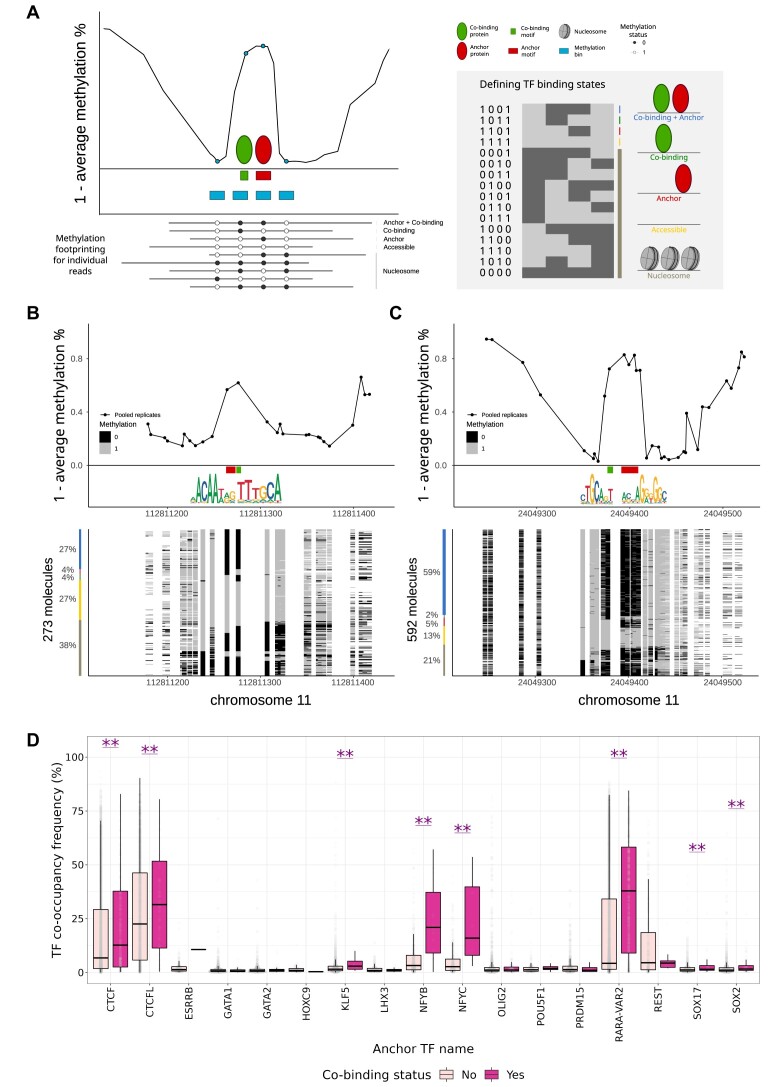
Single-molecule footprinting analysis. (**A**) For each region, single molecules are grouped and assigned to one of the five states (‘Co-binding + Anchor’, ‘Co-binding’, ‘Anchor’, ‘Accessible’, and ‘Nucleosome’) based on the binary cytosine methylation status in and around anchor and co-binding patterns (for two regions of interest, four bins are analysed). (B, C) Single-locus examples of predicted co-bound regions where the anchor TFBSs associate with SOX2 (**B**) and CTCF (**C**). Line plots in the top panels represent the average methylation levels. We provide logos and locations of the anchor motifs (red) and the predicted co-binding motifs (green). Stacks of sorted single molecules are visualized in the lower panels (light grey indicates methylated Cs, accessible positions; black - unmethylated Cs, protected). The y-axis corresponds to the total number of molecules. Coloured bars and percentages show the proportion of molecules in each state (colours corresponding to A). (**D**) We provide boxplots and dotplots showing the distribution of ‘Anchor + Co-binding’ state frequencies of molecules that overlap the regions in two groups: one with predicted co-binding (shown in dark pink) and one with only an anchor TFBS (shown in light pink). The regions with co-binding events show a significantly higher frequency of molecules as co-occupied (‘Anchor + Co-binding’). The differences between groups persist for molecules in other states. ** represents a Wilcoxon test *P*-value < 0.05.

For each genomic region predicted by COBIND to contain a co-binding pattern or not, we determined the fractions of molecules in each of the following five states: (i) accessible, (ii) occupied by nucleosomes, (iii) only occupied at the anchor motif, (iv) only occupied at the co-binding motif or (v) co-occupied at both the anchor and the co-binding motifs. This is done by evaluating the binary methylation status of each molecule for each cytosine in and around the anchor and co-binding pattern sites (Figure 8A). Figure 8B and C illustrates the SMF data analysis at two genomic regions predicted to harbour co-binding events for SOX2 and POU5F1 (Figure 8B) and the extended CTCF motif (Figure 8C). We observed footprints of co-occupancy at both the anchor motif and the co-binding motif locations. Specifically, the analysis of 273 molecules across five replicates revealed co-occupancy for 27% of the molecules when considering the SOX2-associated region (Figure 8B). Five hundred ninety-two molecules across six replicates revealed co-occupancy of the extended CTCF motif for 59% of the molecules (Figure 8C). In contrast, the example regions where COBIND did not predict a co-binding pattern for SOX2 or CTCF, we only observed occupancy for the SOX2 anchor (17% of 89 molecules) or the canonical CTCF sites (46% of 297 molecules) while co-binding sites were unoccupied (Additional file 1: Supplementary Figure S39A-B). When considering all the regions with SMF data, we found a larger fraction of co-occupied anchor and co-binding motif locations on the same molecule in COBIND-predicted co-bound regions than in other regions (*P*-value < 0.001; Additional file 1: Supplementary Figure S39C). Significant differences existed in all state abundances, except for the specific occupancy of the co-binding sites. This agrees with these sites not being occupied when the anchor sites are not either. However, not all anchor TF binding profiles satisfied this observation (Figure 8D). Moreover, we found that genomic regions predicted by COBIND to harbour a co-binding pattern were more occupied (for ‘Anchor + Co-binding’ occupancy state independently) than the other genomic regions for 13 out of 17 anchor TF; statistical significance was observed for 8 TF (*P*-value < 0.05; Figure 8D). Altogether, the results confirmed that SMF data supported the COBIND co-occupancy predictions for some TFs, with the co-bound regions more accessible or co-occupied at single-molecule resolution.

## Discussion

We introduced COBIND, a computational framework for discovering *de novo* space-fixed DNA motif patterns in the vicinity of predetermined genomic regions. We applied COBIND to sets of TFBSs from UniBind to identify locally enriched DNA motifs that define co-binding patterns of cooperative TFs. The method uncovered both established and new co-binding patterns, with most TFs sharing a co-binding motif with other TFs from the same family. Additionally, we inferred, when possible, the TFs most likely co-binding the discovered patterns. We make the collection of co-binding patterns revealed by COBIND available to the scientific community for each of the 214 TFs and 58 TF families across seven species. The co-binding events captured by COBIND are likely functionally relevant since they exhibit higher evolutionary conservation than isolated TFBSs. Furthermore, chromatin and single-molecule footprinting data support the occurrence of the identified co-binding events on the same DNA molecules.

We used a combination of motif similarity and prior knowledge of PPIs to identify TFs that may cooperatively bind to the patterns discovered by COBIND. Identifying well-known TF co-binding events, such as POU5F1 with SOX2 or SOX17, essential pluripotency regulators (15,16,65), confirmed the approach's efficacy. Importantly, we used a multi-species collection of DNA binding motifs to predict new co-binding configurations for pairs of TFs currently unsupported by PPIs. We discovered that NF-Y proteins are potential partners for HY5, ABF1, 3–4 and GBF2-3 TFs. Previous studies identified NF-Y proteins as co-binders for bZIP67 and bZIP28 proteins belonging to the same TF family (58–60). More specifically, other studies also pointed to physical interactions of either HY5 or ABF1, 3–4 with NF-YC9 proteins (66,67). We identified the NF-Y-bound co-binding motifs using motifs associated with human TFs because DNA binding profiles for NF-Y are limited in *A. thaliana*. As HY5 competes with bZIP28, we acknowledge that the NF-Y binding pattern observed close to HY5 TFBSs might be occupied only when bZIP28 is binding. Nevertheless, using multi-species libraries generated potential co-binding predictions that will require further validations to decipher the binding cooperation of TFs at the corresponding genomic regions.

We observed that the COBIND predictions associated with CTCF anchor TFBSs exhibited a binding pattern that did not result from the co-binding of two TFs. Previous studies have discussed this binding pattern and have suggested that CTCF co-binds with ZBTB3 in mouse liver and various human cell lines, including embryonic stem cells and K562 (68,69). Based on motif similarity, we inferred that ZBTB3 could also bind the additional motif identified by COBIND. Instead, the co-binding pattern identified by COBIND corresponds to an extended binding motif for CTCF, involving zinc fingers 9–11 (61). This orthogonal evidence allowed the two variants of extended CTCF motifs to complement the JASPAR database (MA1930.1 and MA1929.1) (7,70). Furthermore, this binding pattern is critical for optimal CTCF protein residence time on DNA (61). Importantly, we found that the genomic regions harbouring the extended motif were conserved in both mouse and human genomes, supporting the functional relevance of this binding pattern. Studies frequently reported that zinc-finger proteins bind DNA through a subset of their zinc fingers, but it remains unclear whether the other fingers have additional binding preferences (71). Thus, binding to extended motifs with additional zinc fingers may be a common characteristic of zinc finger proteins that *de novo* motif prediction tools like COBIND could capture.

The conservation of TFBSs is an essential indicator of their functional relevance in gene regulation (47). Furthermore, the combinatorial binding of TFs is important for evolutionary stability, and increased co-binding of TFs associates with a higher probability of a regulatory region being conserved (72). This study analysed the conservation of co-binding patterns across different species. The regions predicted to harbor TFBSs with fixed spacing were more evolutionarily conserved than those with a single TFBS. The increase in conservation at regions where multiple TFs are likely co-binding compared to single events confirms other reports showing that (i) TFBSs of cooperative TFs are more evolutionarily conserved in mammalian embryonic stem cells (73) and drosophila (74) and (ii) that TF cooperativity can increase speed of evolution (75). Interestingly, not only were the predicted co-bound motif locations conserved, but the anchor motif locations also showed an increase in conservation, which agrees with the previous reports. We found most co-binding patterns with significant conservation from the human and mouse datasets, which may reflect the more significant number of datasets analysed for these species. Overall, the increased conservation of both the anchor and co-binding sites was found in more datasets than expected by chance. However, this was not observed for *A. thaliana*; we hypothesize that this is due to the limited number of conservation tracks, which was restricted to flowering plants (48).

Complementing the conservation analysis, similar results were observed in the DHS footprinting data. Predicted co-binding patterns, along with their anchors, exhibited deeper footprints compared to single TFBSs. A lower signal may indicate a higher false positive rate for single binding events. However, it can also reflect single binding events occurring in a smaller subset of cells while cooperative binding would be shared across cells. Future experiments probing TF binding at single-cell resolution would allow for assessing this hypothesis.

COBIND is a tool for the *de novo* discovery of co-occurring DNA patterns. Our synthetic data assessment found that STREME performed well in retrieving the injected motifs *de novo*. However, it underperformed with small numbers of sequences. Moreover, STREME, unlike COBIND, is not intended to capture specific cooperative binding events; therefore, capturing the specific spacings and orientations relative to an anchor motif would require specific post-processing steps. Importantly, these tools do not require *a priori* knowledge of DNA motifs, such as a known DNA motif binding library, to perform the discovery. The requirement of known motif collections as an input is a standard limitation for other tools dealing with TF binding analysis, such as SpaMo or TACO (20,21). This reliance on existing collections of DNA binding motifs can be problematic as many TF binding motifs still need to be discovered or included. The only input required for COBIND is a set of genomic reference regions to ‘anchor’ the analysis and generate regions where it will search for patterns. The *de novo* discovery can reveal potential new or unannotated DNA pattern motifs, thereby circumventing the constraints of other tools. As such, COBIND offers a powerful alternative to other motif discovery tools that rely on existing collections of DNA binding motifs. Nonetheless, a limitation is that additional analysis will be required to interpret newly discovered motifs.

In this study, we applied COBIND to genomic regions near TFBSs. However, one can use COBIND in different biological contexts. As a test case, we generated anchor regions for analysis by taking two nucleotides at the donor and acceptor sites of intron-exon boundaries in the human genome. Analysis with COBIND recovered known donor and acceptor motifs (Additional file 2: Supplementary Figure S40), demonstrating the versatility of the approach for DNA pattern discovery in other types of biological problems.

We utilized the available SMF data to assess the co-occupancy of the anchor and co-binding motif instances on the same DNA molecules (52). A limitation of the assay is that one can only apply it to organisms and cell lines that can survive without endogenous methylation. We restricted our analyses to mouse ESCs, for which SMF was already available. A recent study assessed chromatin openness using methylation in the GpC context, allowing applications to different cell types with larger genomic coverage (76). The SMF data we used was produced with a targeted sequencing approach. Consequently, only a selected set of genomic regions was analyzed, and the overlap with regions containing COBIND-predicted co-binding patterns was incomplete, limiting the full understanding of possible co-occupancy on single molecules.

We recognize that COBIND and this study have several limitations. A fundamental limitation of COBIND is the restricted prediction of co-binding motifs with fixed spacing with the anchor motif. Because of the strict spacing requirements and the limitations of the NMF, COBIND restricts the search space to the close proximity of TFBSs. Previous studies have hypothesized the existence of two distinct sets of enhancers with distinct information-processing mechanisms (10). One proposed mechanism distinguishes between enhancers where TF binding is highly cooperative and coordinated with fixed spacing, referred to as the ‘enhanceosome’ model, and enhancers where TF cooperation is flexible, known as the ‘billboard’ model (77). Regardless, these mechanisms are compatible with the presence of both fixed and flexible cooperation of TFs at regulatory elements, depending on the type of TF cooperation. Recent studies support both mechanisms, and even though some suggest that TFBS spacing and orientation are not key determinants of transcription regulation, other studies contradict this statement (10–14). The evolutionary conservation of the co-binding patterns revealed by COBIND further argues for the functional relevance of a strict grammar for a set of TFs and binding regions.

COBIND relies on the NMF algorithm to discover the co-binding patterns, so it requires enough sequences harbouring the fixed pattern. Our simulated data showed that we obtained reliable results when at least 10% of at least 800 sequences (also 5% of 1000 sequences) contained the pattern to be predicted by COBIND. Since we considered the anchor TFBS and the flanks independently in the COBIND processing, discovering overlapping motifs represents a challenge. COBIND could detect such overlaps by revealing a small co-binding motif, but interpreting such results becomes increasingly difficult as the partial motif becomes small. Furthermore, we used anchor TFBSs predicted from TF binding profiles corresponding to the canonical motifs recognized by the corresponding TFs. This approach prohibits the identification of co-binding patterns where the anchor TFs would recognize altered motifs when cooperating with other proteins. Furthermore, repetitive and low-complexity regions are established issues for *de novo* motif discovery tools (78,79). For instance, A/T-rich patterns with low complexity, such as A/T stretches, were discovered in 0.5% of random genomics regions matching the %GC composition of the real datasets used for the DHS analysis (Additional file 2: Supplementary Figure S41-Supplementary Figure S42). Therefore, we recommend that users interpret such patterns cautiously. Finally, another limitation lies in the computational time necessary for motif clustering when the NMF identifies many possible motifs. Nevertheless, our comparison to other tools revealed that COBIND’s computational time was not prohibitive.

## Supplementary Material

gkae743_Supplemental_Files

## Data Availability

COBIND is implemented in Python, R, and C++. The COBIND source code and documentation are available at https://bitbucket.org/CBGR/cobind_tool/src/main/. The source code and data to reproduce the results outlined in this report are available at https://bitbucket.org/CBGR/cobind_manuscript/src/master/ and pre-processed data deposited at https://doi.org/10.5281/zenodo.7681482. All results presented here are available to the community through a webpage at https://cbgr.bitbucket.io/COBIND_results_page/.
