# Supplementary material for: Identification of transcription factor co-binding patterns with non-negative matrix factorization
Source: Nucleic Acids Res. 2024 Sep 1;52(18):e85. doi: 10.1093/nar/gkae743 (PMC11472169; doi:10.1093/nar/gkae743)
Supplement: gkae743_Supplemental_Files [file gkae743_supplemental_files.zip › additional_file_1.pdf]

IDENTIFICATION OF TRANSCRIPTION FACTOR CO-BINDING PATTERNS WITH NON-NEGATIVE MATRIX FACTORIZATION

Ieva Rauluseviciute[1] Timothée Launay[1] Guido Barzaghi[2,3]
Sarvesh Nikumbh[4,5] Boris Lenhard[4,5] Arnaud Regis Krebs[2]
Jaime A. Castro-Mondragon[1] Anthony Mathelier[1,6,7,*]

[1] Centre for Molecular Medicine Norway (NCMM), Nordic EMBL Partnership, University of Oslo, 0318 Oslo, Norway
[2] European Molecular Biology Laboratory (EMBL), Genome Biology Unit, Meyerhofstraße 1, 69117 Heidelberg, Germany
[3] Collaboration for Joint Ph.D. degree between EMBL and Heidelberg University, Heidelberg, Germany
[4] MRC London Institute of Medical Sciences, Du Cane Road, London, W12 0NN, UK
[5] Institute of Clinical Sciences, Faculty of Medicine, Imperial College London, Hammersmith Hospital Campus, Du Cane Road, London, W12 0NN, UK
[6] Department of Medical Genetics, Institute of Clinical Medicine, University of Oslo and Oslo University Hospital, Oslo, Norway
[7] Center for Bioinformatics, Department of Informatics, Faculty of Mathematics and Natural Sciences, University of Oslo, Oslo, Norway

[*] Correspondence: Anthony Mathelier <anthony.mathelier@ncmm.uio.no>

ADDITIONAL FILE 1

SUPPLEMENTARY FIGURES

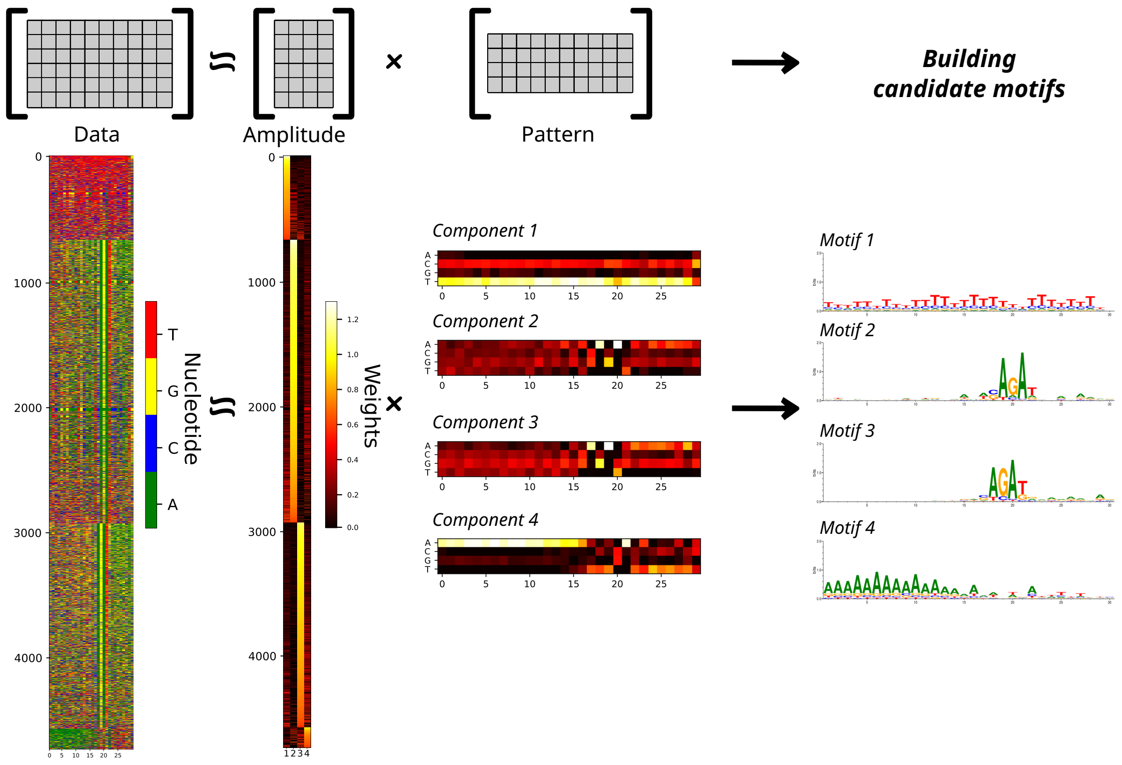

Figure S1. **Non-negative matrix factorization discovers sequence patterns from one-hot encoded sequences.** The factorization results in amplitude and pattern matrices with a defined number of components k (in this example, k=4). The pattern matrices reveal candidate pattern motifs. We seek motifs with highly condensed information content (IC) surrounded by positions with low IC. For example, motifs 2 and 3 have a higher Gini coefficient than motifs 1 and 4, for which we observe an even distribution of IC values along entire motif.

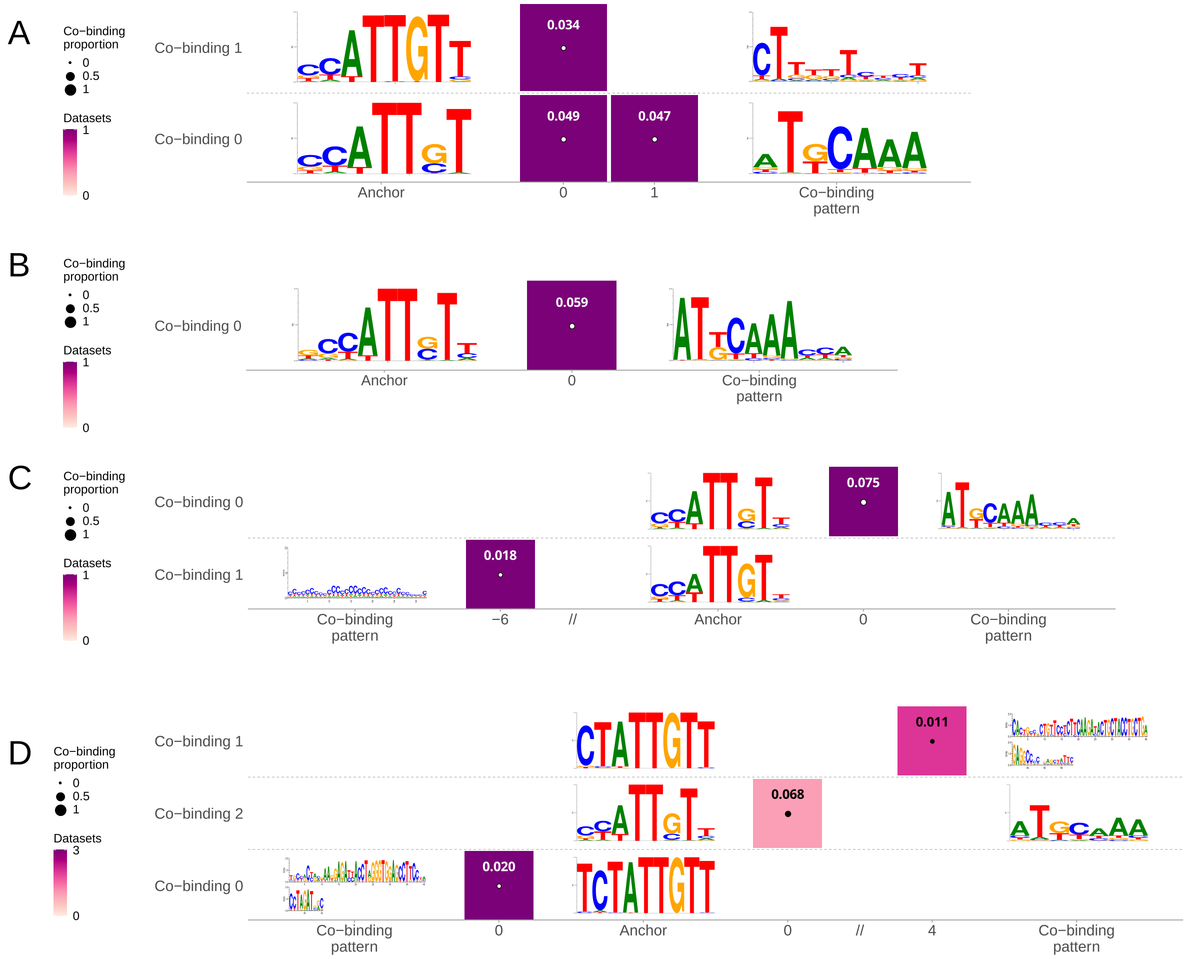

Figure S2. **Investigating larger flanking regions results in noisier co-binding motifs.** The figure presents the co-binding patterns discovered by COBIND when applied to genomic regions flanking the anchor motif, considering 30bp (**A**), 50bp (**B**), 100bp (**C**), and 150bp (**D**). Considering 50bp captures only one spacing configuration and loses one potential pattern. Considering 100bp discovered an extra non-informative motif. When considering 150bp, additional long and hard to interpret patterns are discovered.

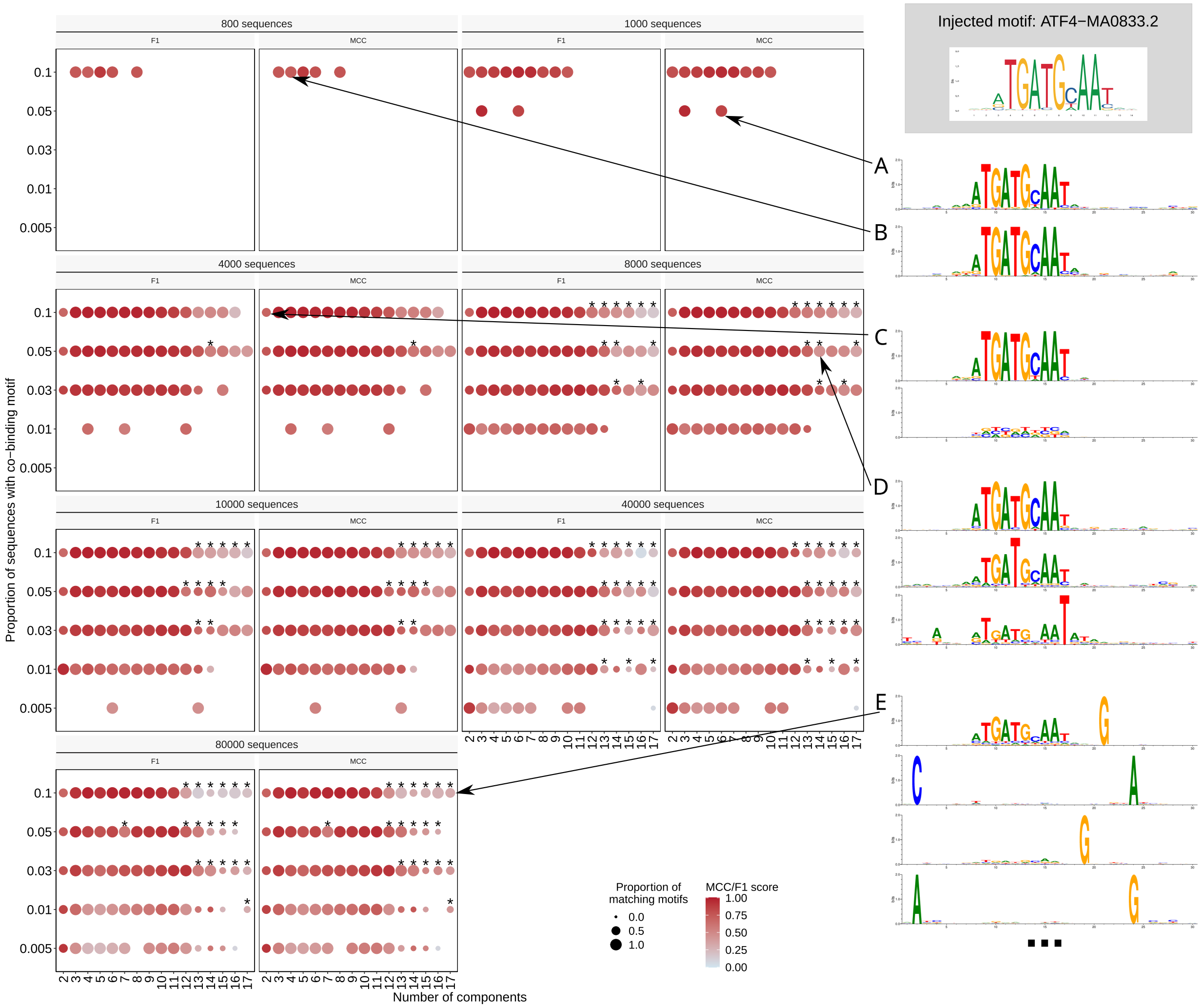

Figure S3. **Simulation experiments.** We ran NMF on random sequences in which we injected instances of a known motif (JASPAR MA0833.2 TF binding profile for ATF4) (see Methods). (**A-B**) The inserted motif is captured in many instances; examples of motifs discovered with 3 or 6 components NMF are presented. We observed that F1 scores and Matthew's correlation coefficients (MCC) are high and no incorrect motif is predicted. (**C**) When considering two components, we observed that an incorrect motif is predicted. (**D**) When using larger numbers of components, the correct motif is discovered multiple times (indicated by * when a correct motif is discovered more than once), which leads to lower F1 and MCC scores for each motif. (**E**) With a sufficiently large number of components, the injected motif gets decomposed over numerous clusters, which leads to partial motif discovery.

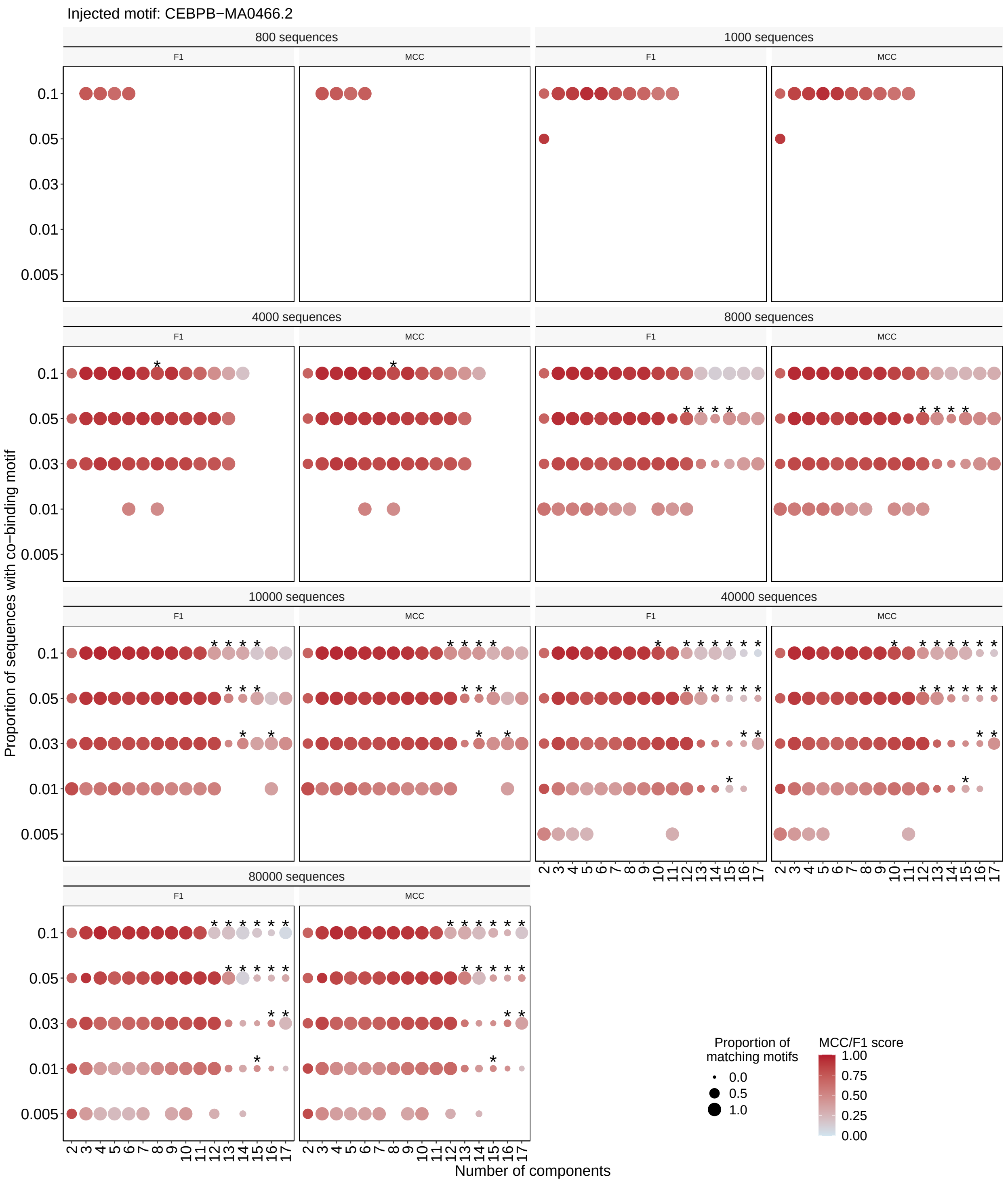

Figure S4. **Simulation experiments.** Same as Figure S3 but injecting instances of the JASPAR MA0466.2 TF binding profile, CEBPB.

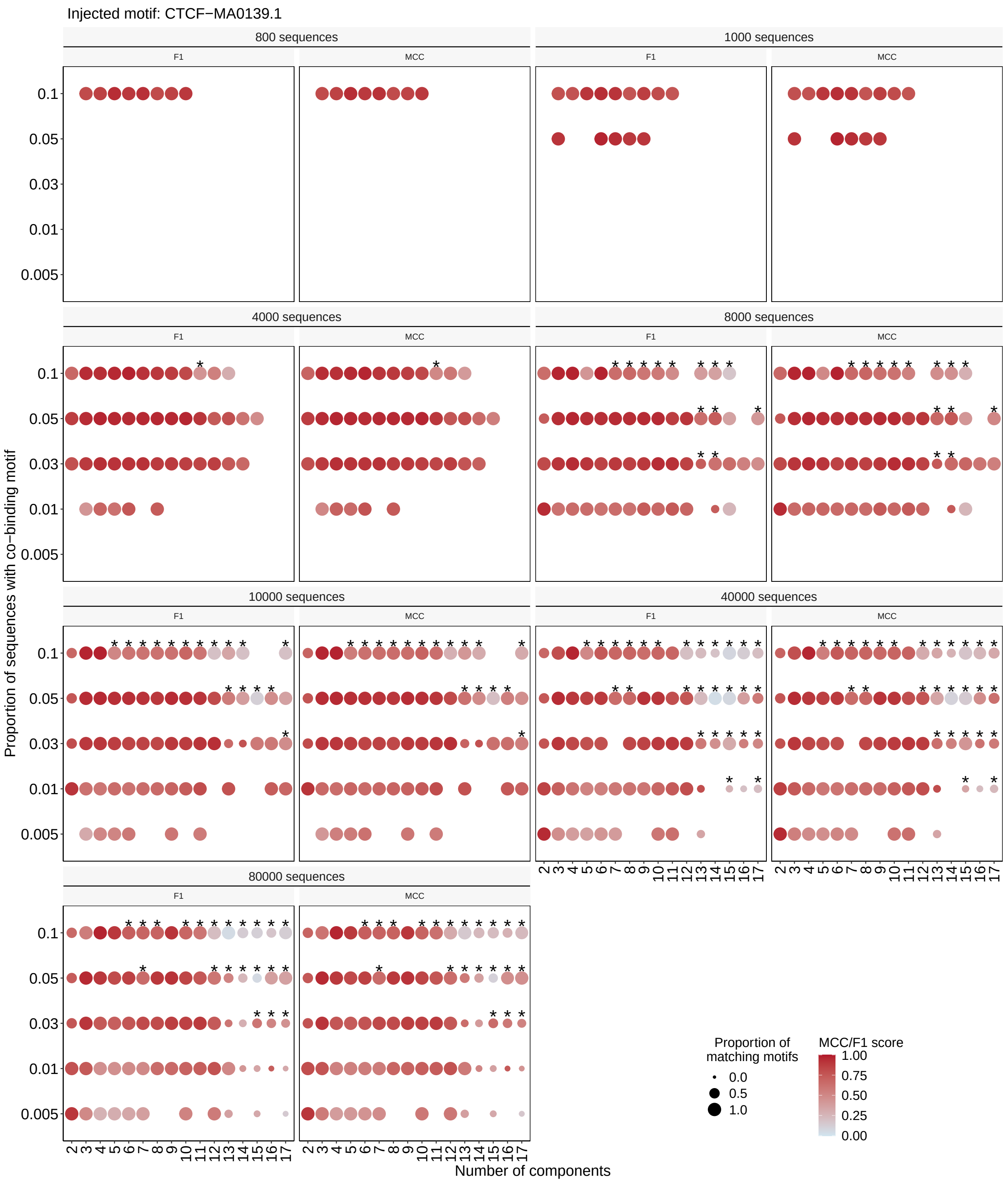

Figure S5. **Simulation experiments.** Same as Figure S3 but injecting instances of the JASPAR MA0139.1 TF binding profile, CTCF.

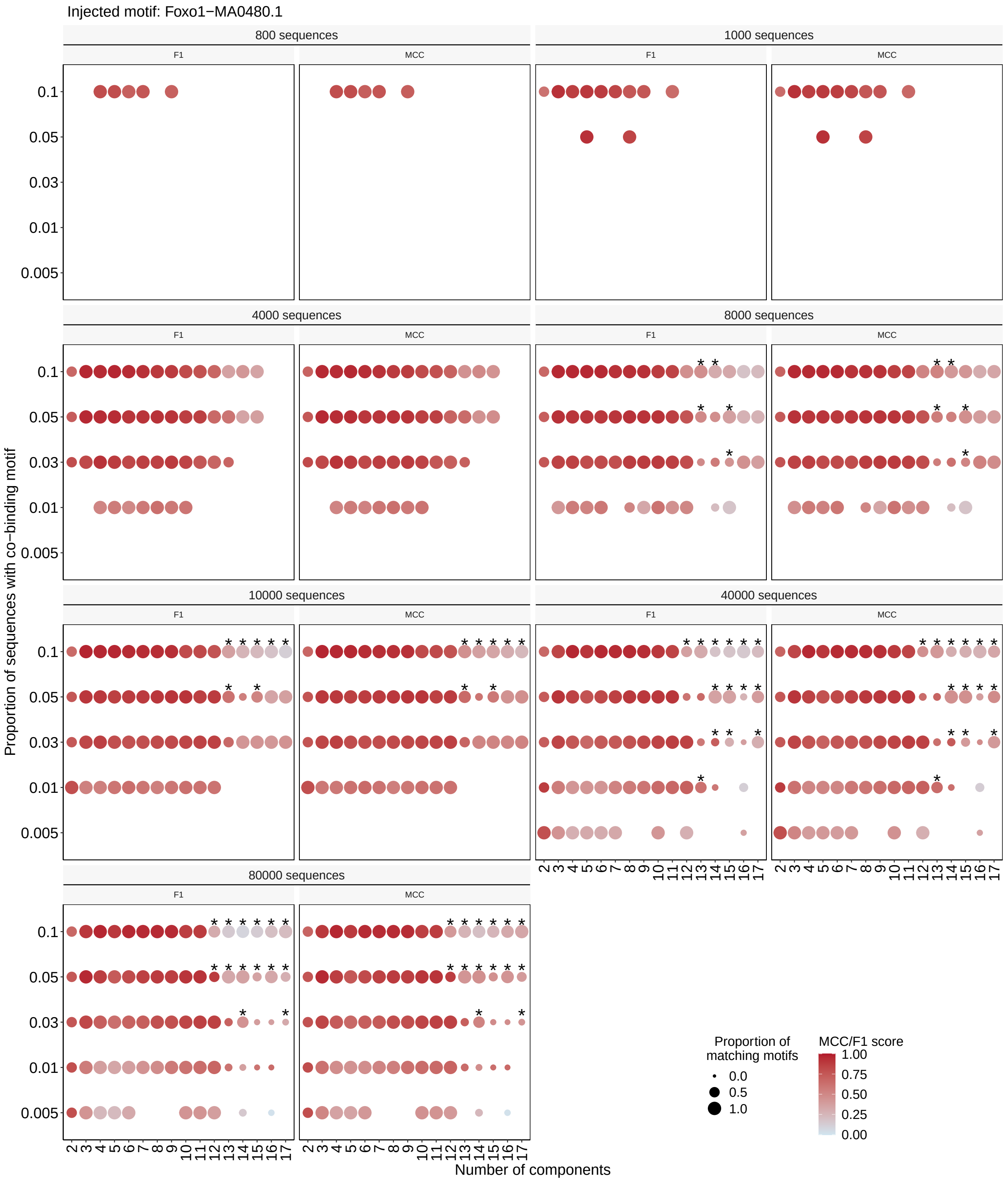

Figure S6. **Simulation experiments.** Same as Figure S3 but injecting instances of the JASPAR MA0480.1 TF binding profile, Foxo1.

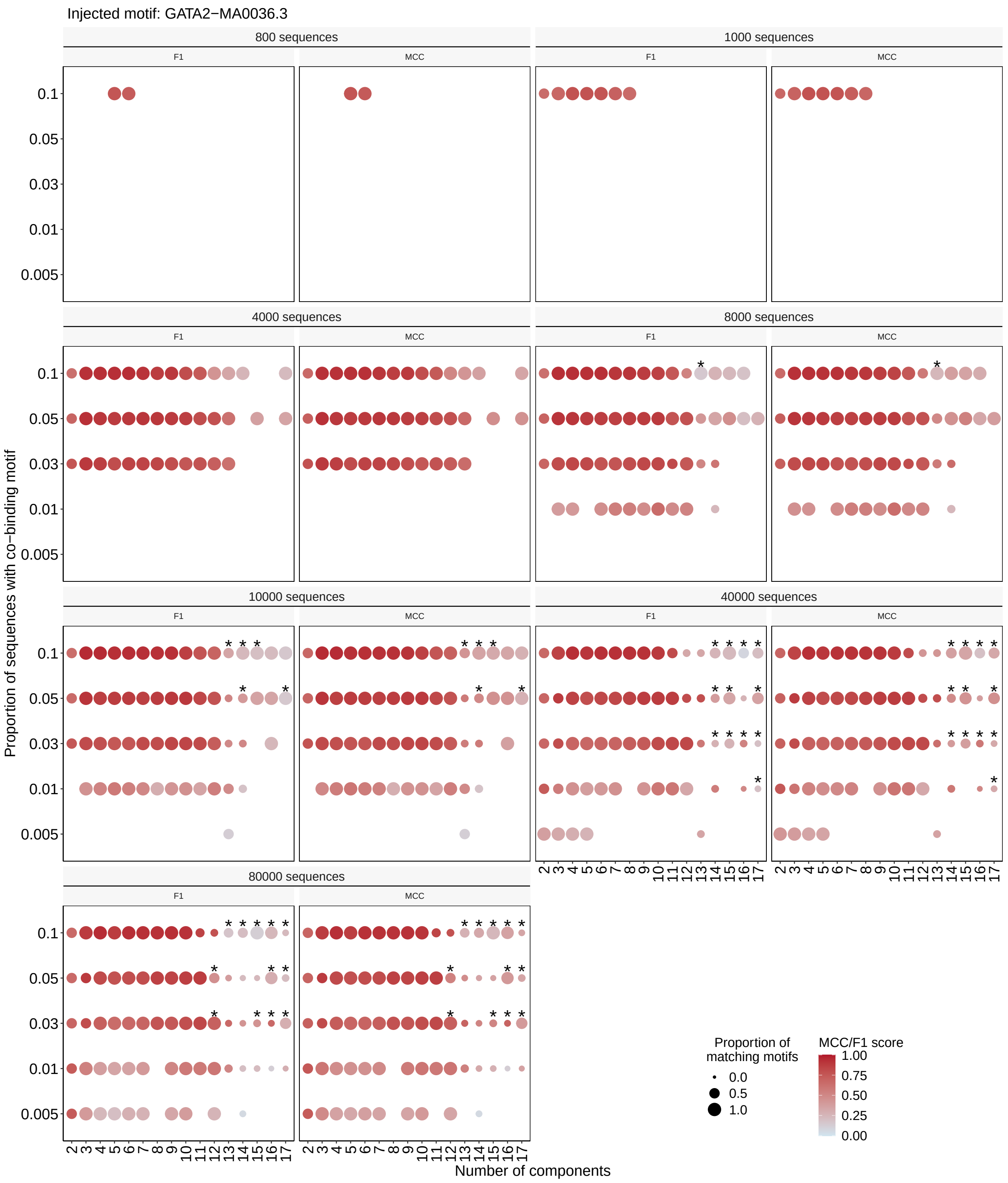

Figure S7. **Simulation experiments.** Same as Figure S3 but injecting instances of the JASPAR MA0036.3 TF binding profile, GATA2.

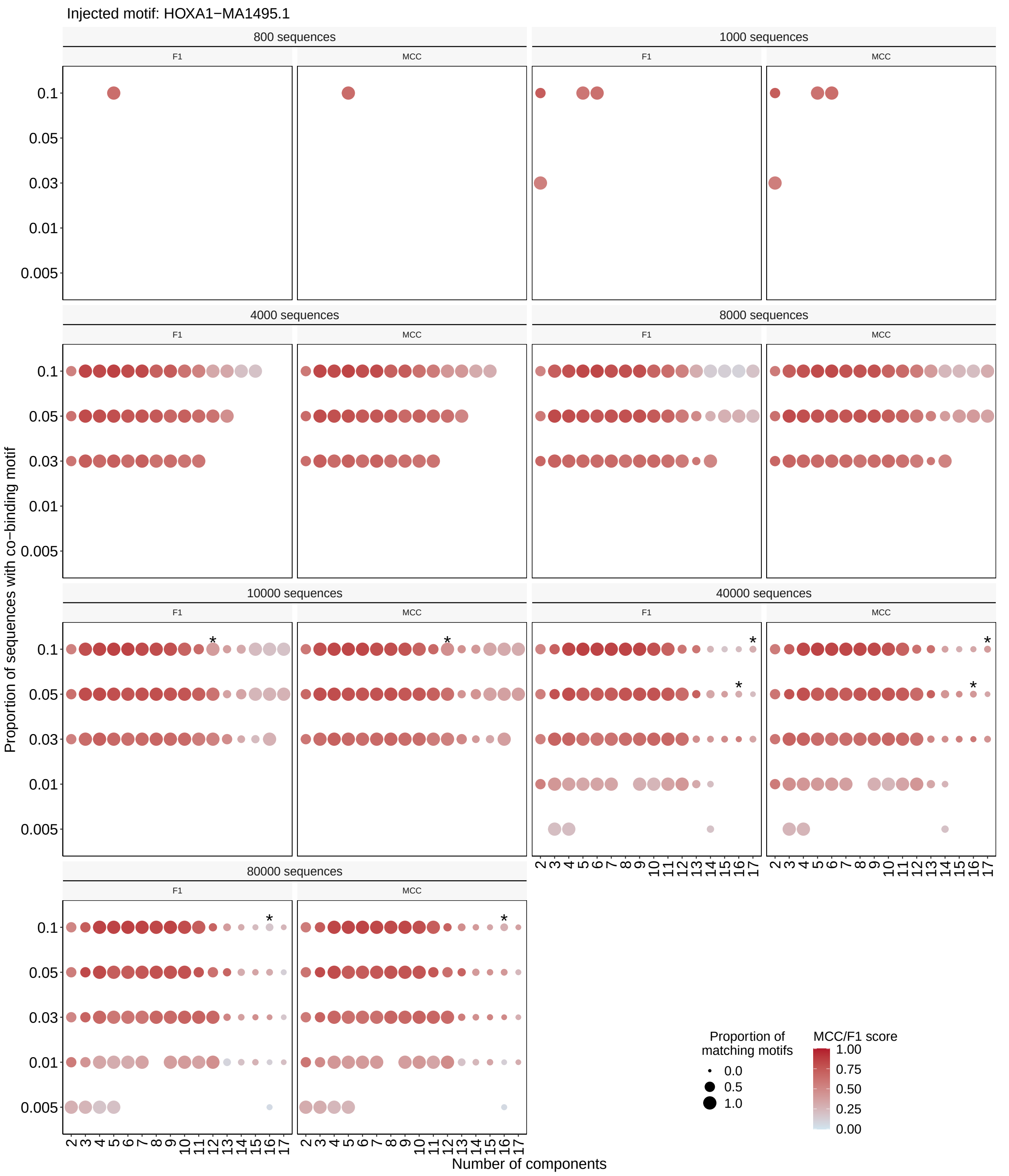

Figure S8. **Simulation experiments.** Same as Figure S3 but injecting instances of the JASPAR MA1495.1 TF binding profile, HOXA1.

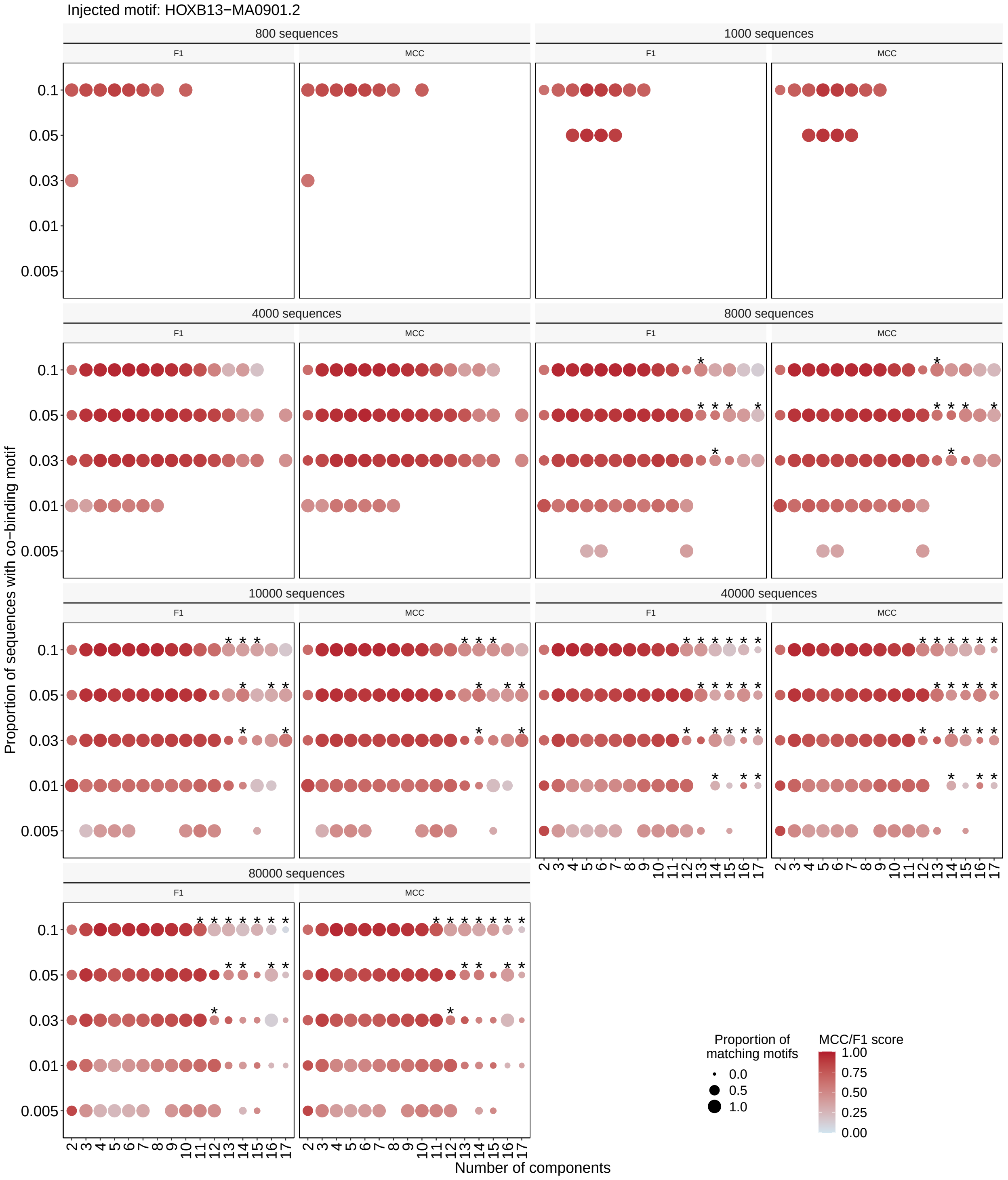

Figure S9. **Simulation experiments.** Same as Figure S3 but injecting instances of the JASPAR MA0901.2 TF binding profile, HOXB13.

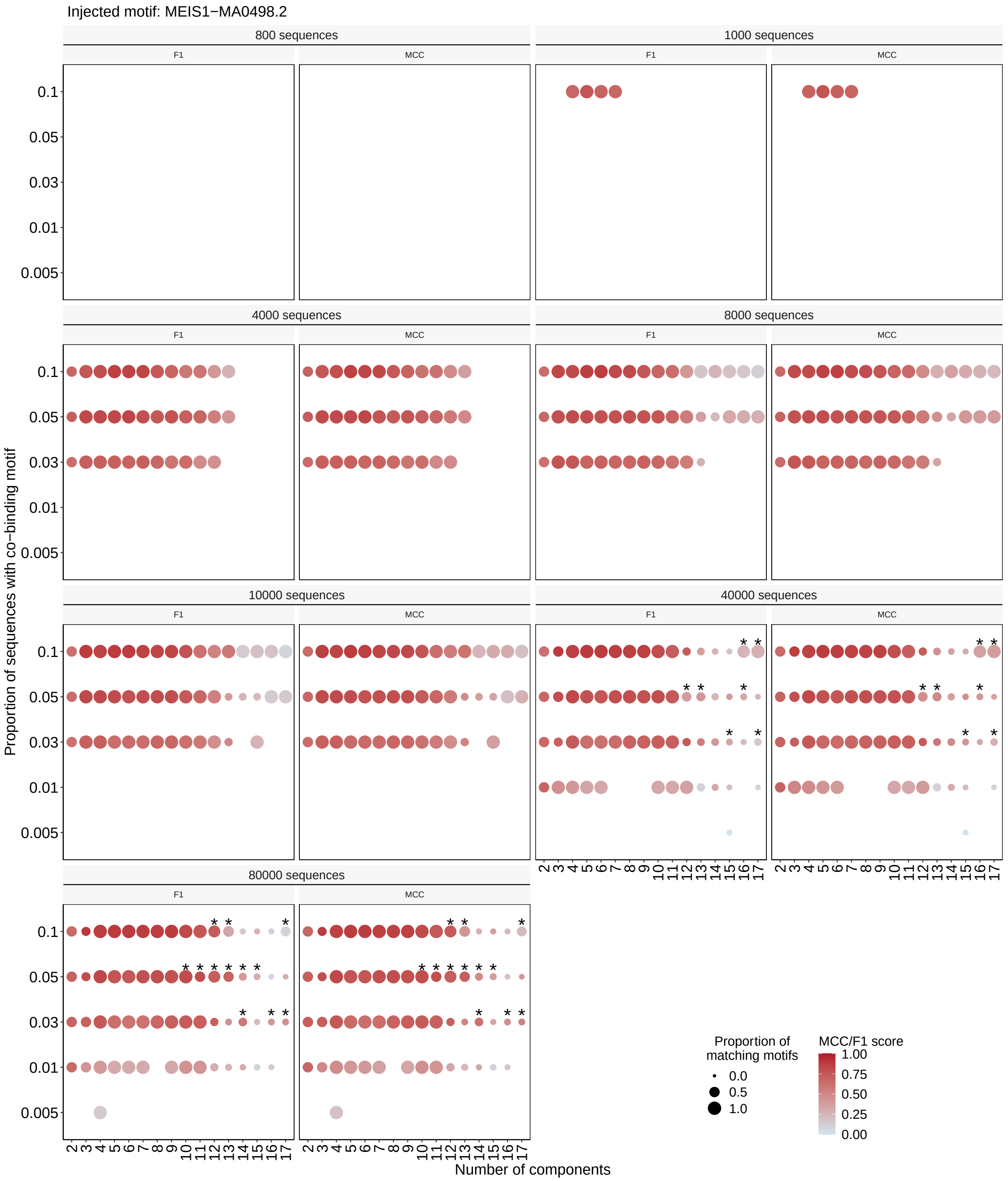

Figure S10. **Simulation experiments.** Same as Figure S3 but injecting instances of the JASPAR MA0498.2 TF binding profile, MEIS1.

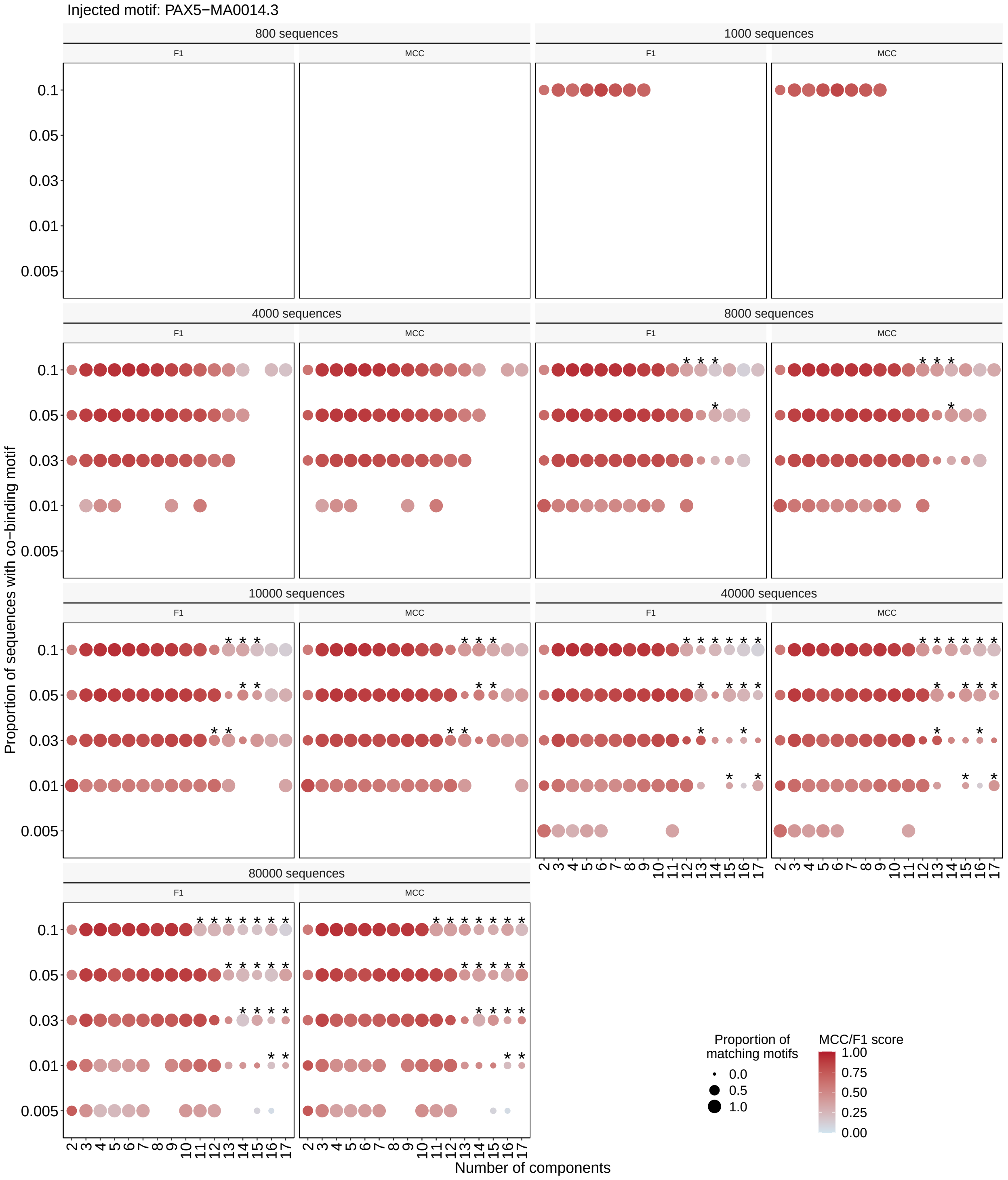

Figure S11. **Simulation experiments.** Same as Figure S3 but injecting instances of the JASPAR MA0014.3 TF binding profile, PAX5.

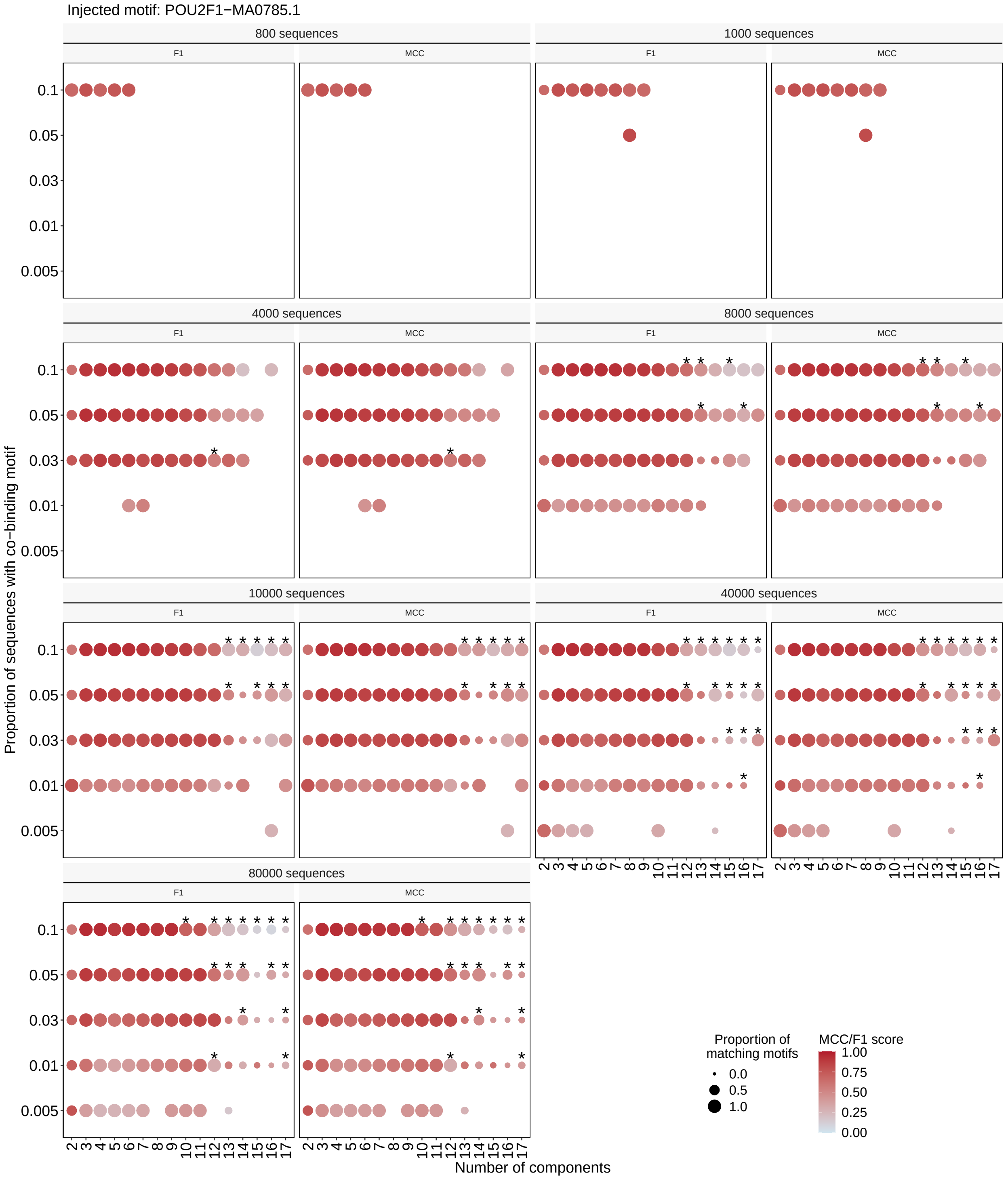

Figure S12. **Simulation experiments.** Same as Figure S3 but injecting instances of the JASPAR MA0785.1 TF binding profile, POU2F1.

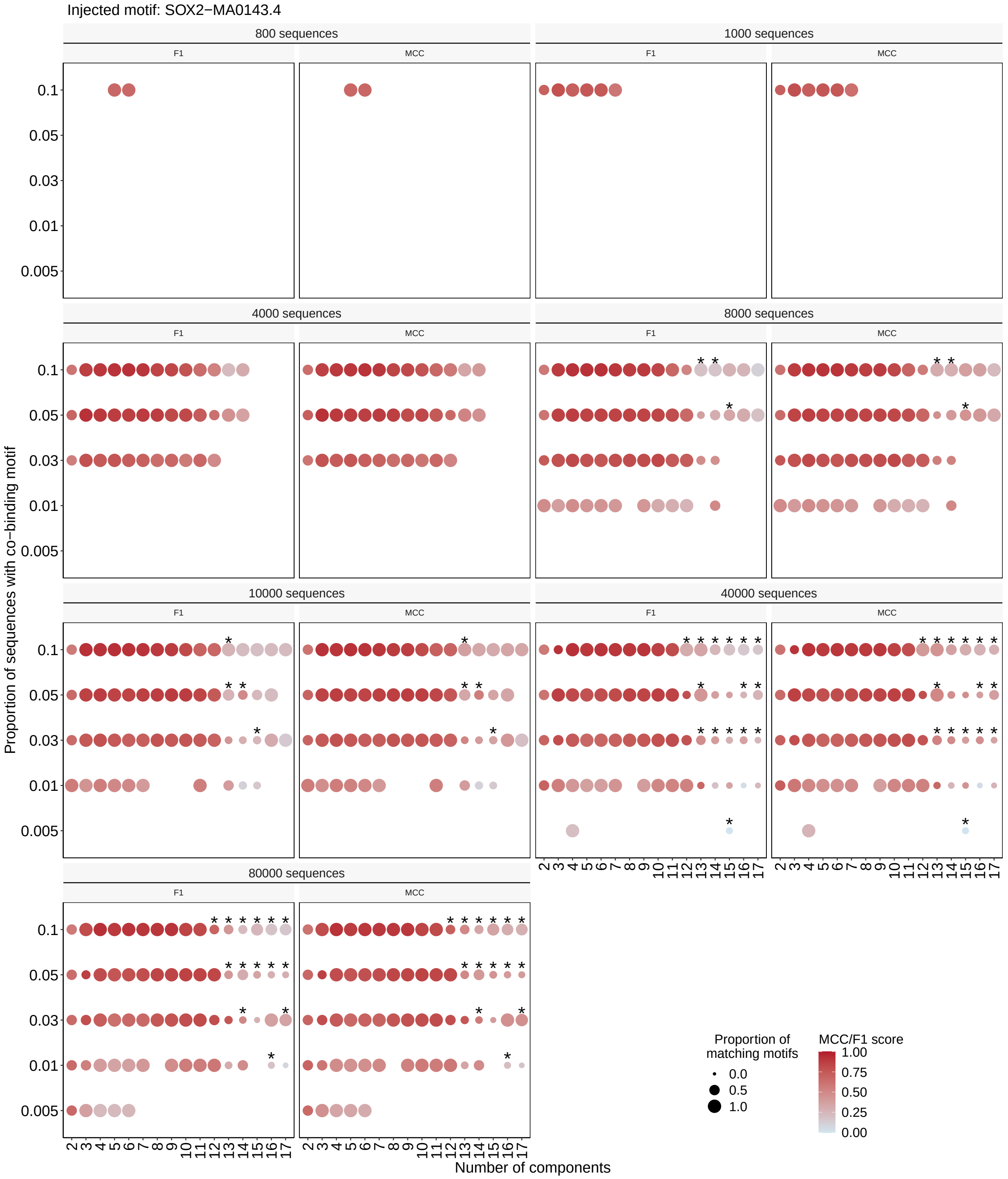

Figure S13. **Simulation experiments.** Same as Figure S3 but injecting instances of the JASPAR MA0143.4 TF binding profile, SOX2.

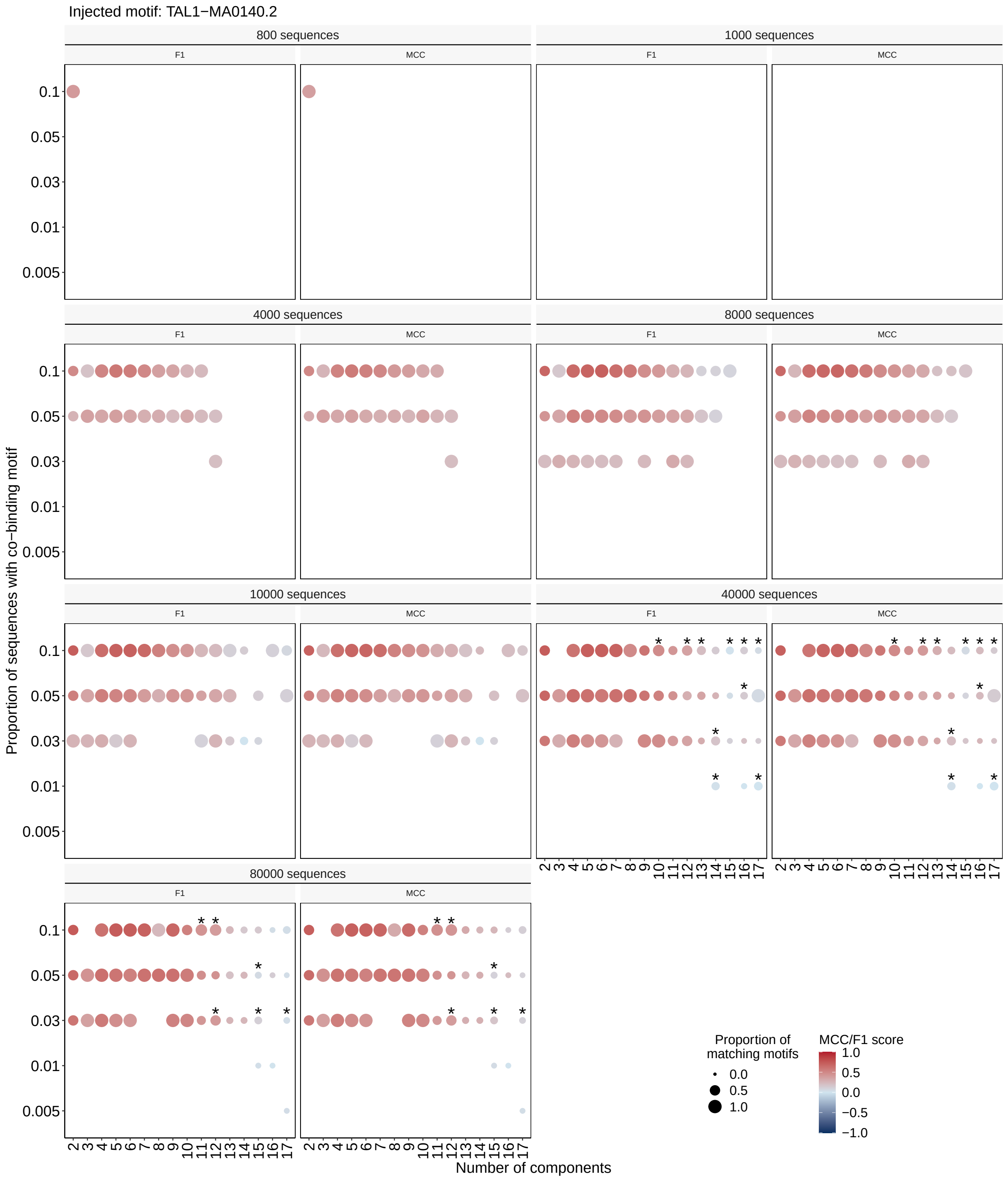

Figure S14. **Simulation experiments.** Same as Figure S3 but injecting instances of the JASPAR MA0140.2 TF binding profile, TAL1.

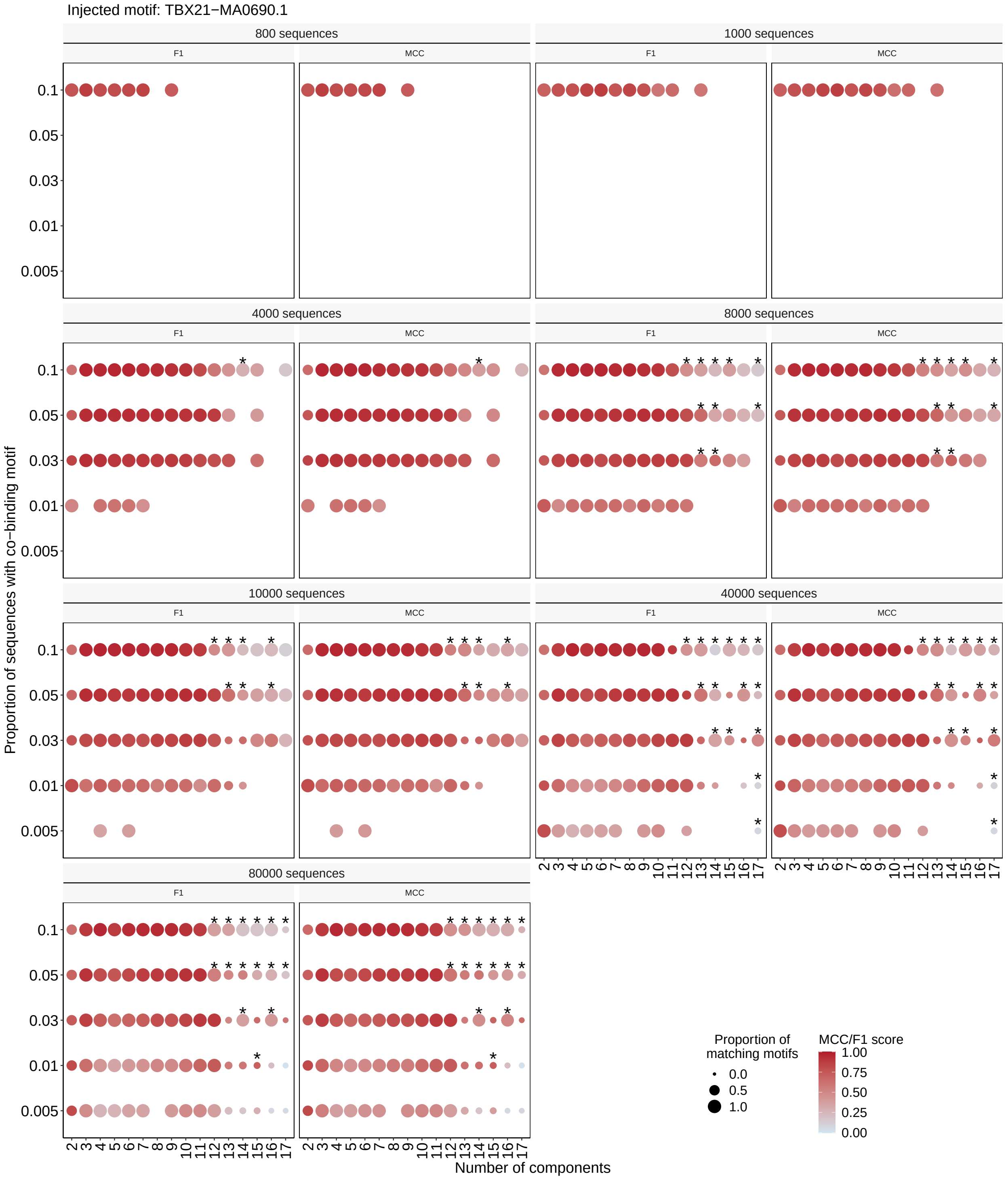

Figure S15. **Simulation experiments.** Same as Figure S3 but injecting instances of the JASPAR MA0690.1 TF binding profile, TBX21.

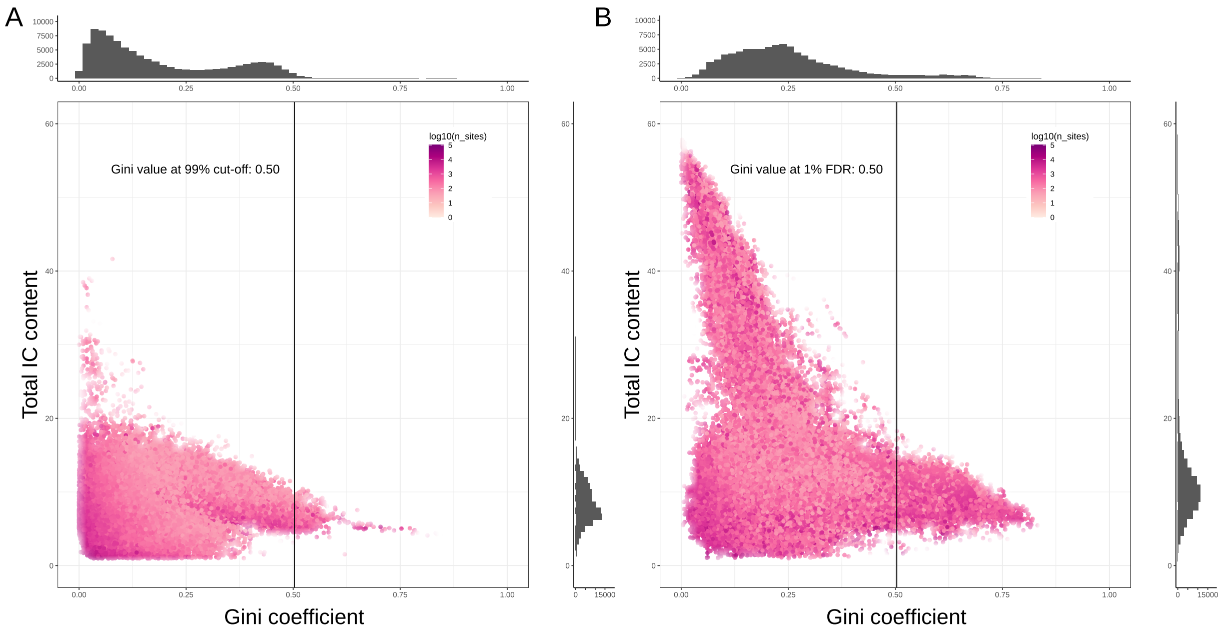

Figure S16. **Species-specific Gini coefficient thresholding using simulated human data.** (**A**) Distribution of Gini coefficients and information content (IC) values for the motifs discovered in simulated data (see Methods). The vertical line indicates the 99th percentile of the Gini distribution (equal to 0.5). (**B**) Distribution of Gini coefficients and information content (IC) values for the motifs discovered in the real human data. The vertical line represents a Gini coefficient cut-off value of 0.5, derived from the simulated data (A).

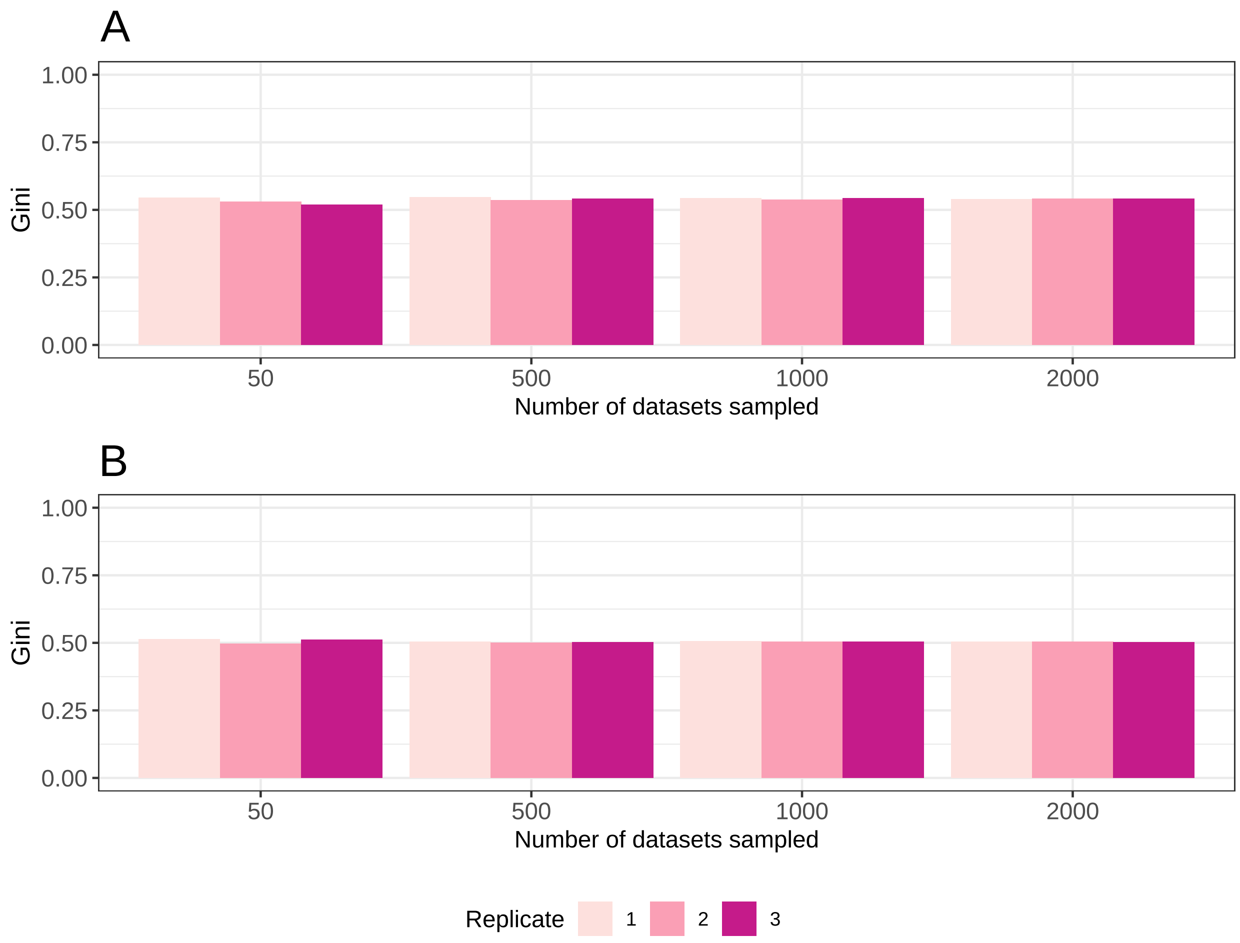

Figure S17. **Consistency of the Gini coefficient thresholds.** We observed consistent Gini coefficient thresholds (y-axis) derived from human (**A**) and mouse (**B**) simulated data from different numbers of datasets (x-axis) with three replicates.

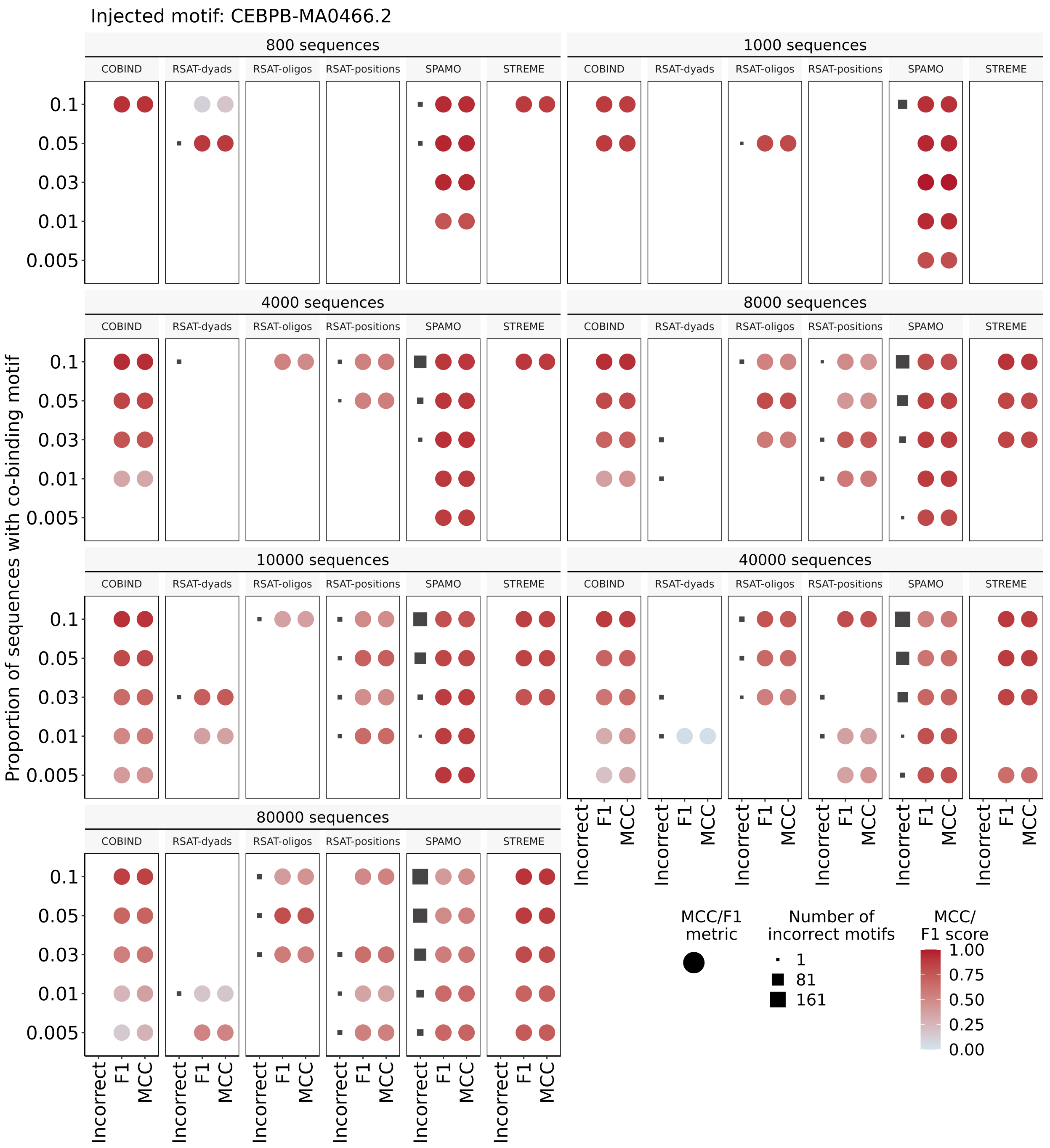

Figure S18. **Comparisons between COBIND and other tools on simulated data.** We ran COBIND, STREME, RSAT-dyads, RSAT-oligos, RSAT-positions, and SPAMO (subfacets) on simulated data containing different numbers of sequences (from 800 to 80,000, see facets) with inserted instances of the JASPAR MA0466.2 TF binding profile for CEBPB (see Methods for details). We considered (i) F1 scores and Matthew's correlation coefficient (MCC) (in case multiple correct motifs are found, results for the motif with the highest F1 score are provided), and (ii) the number of incorrect motifs discovered in each experiment. Good performance of a tool would be indicated by a discovered motif with high F1 score and high MCC and no incorrectly discovered motif.

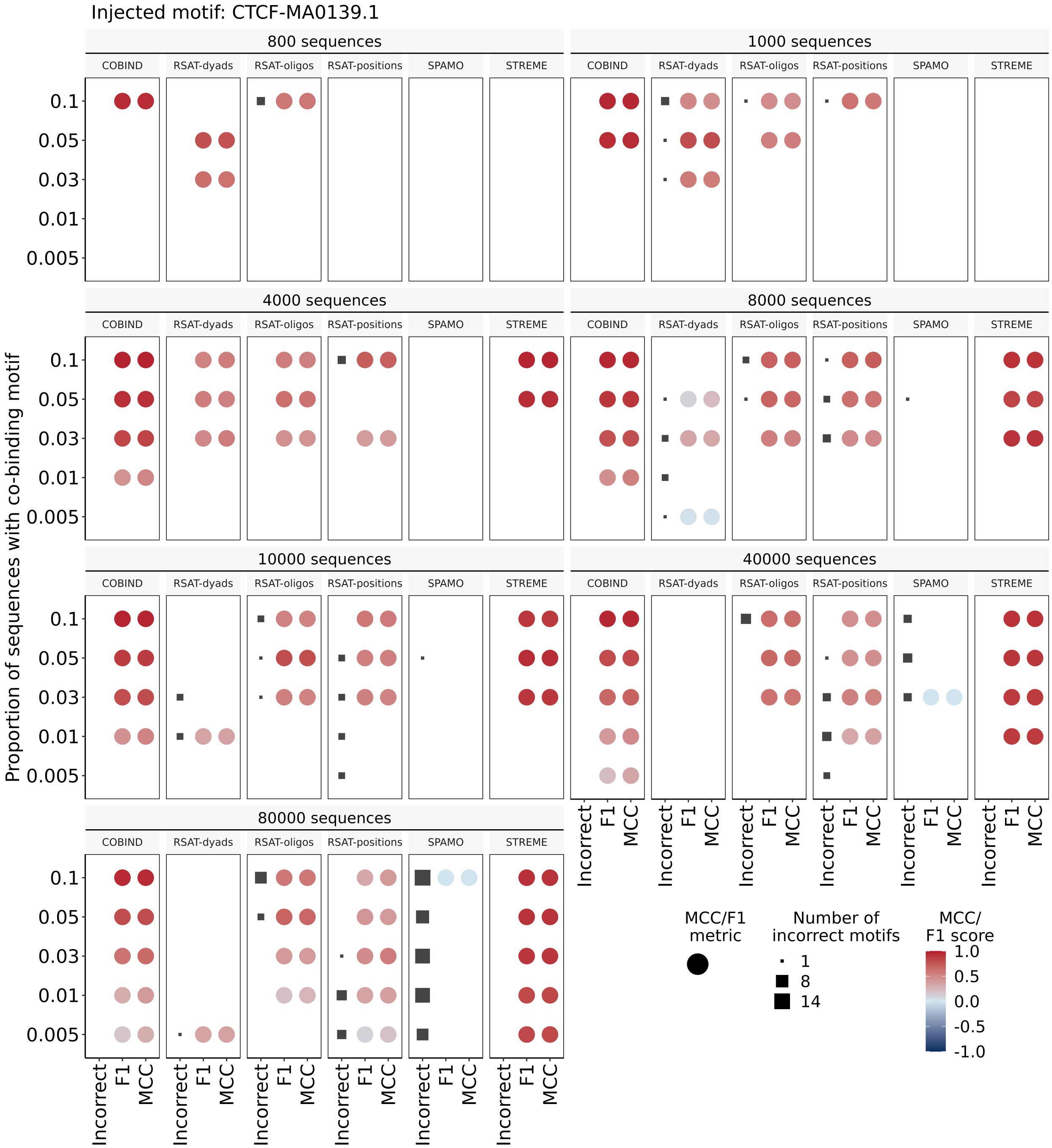

Figure S19. **Comparisons between COBIND and other tools on simulated data.** Same as Figure S18, but with instances of the JASPAR MA0139.1 TF binding profile for CTCF.

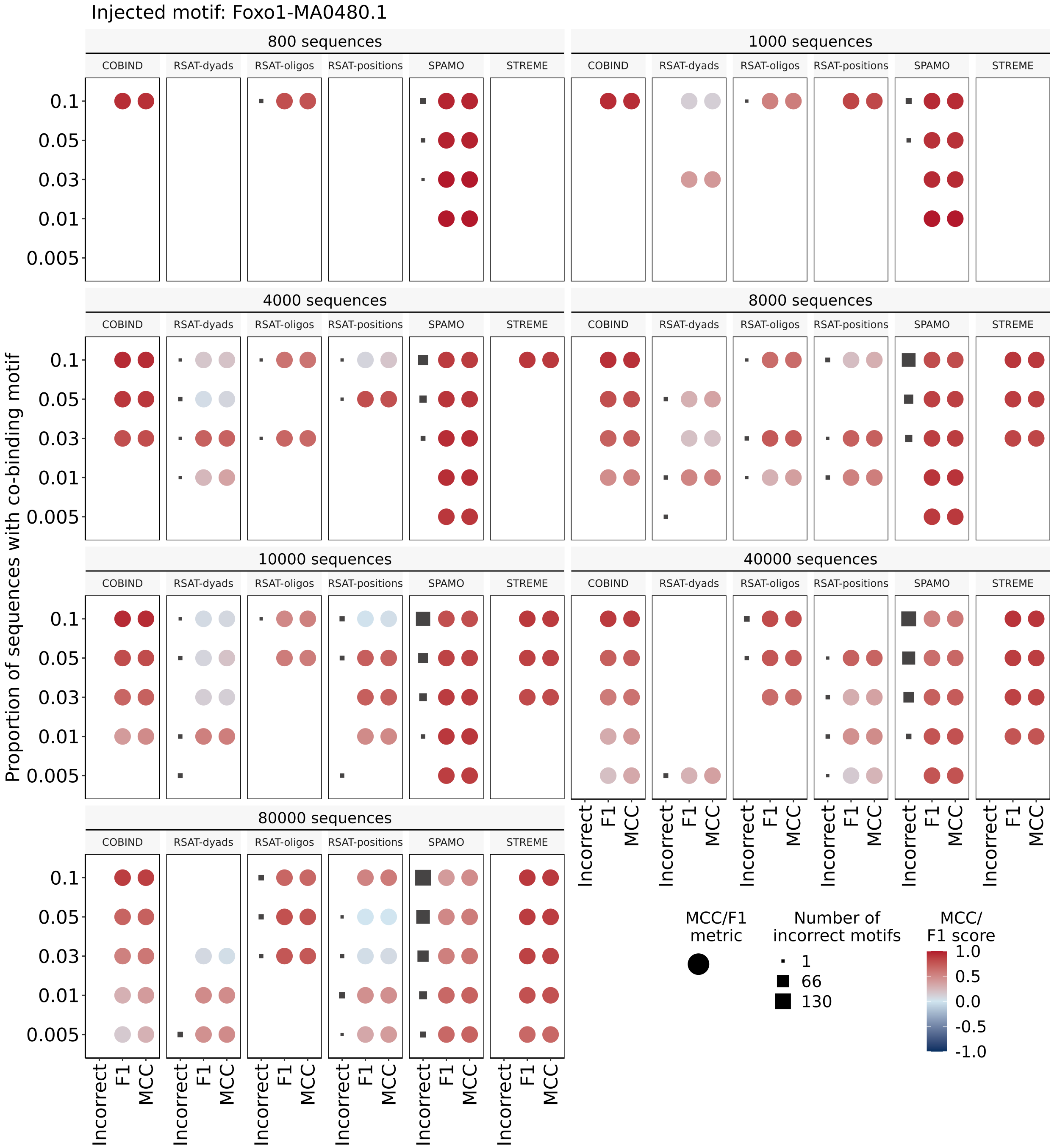

Figure S20. **Comparisons between COBIND and other tools on simulated data.** Same as Figure S18, but with instances of the JASPAR MA0480.1 TF binding profile for Foxo1.

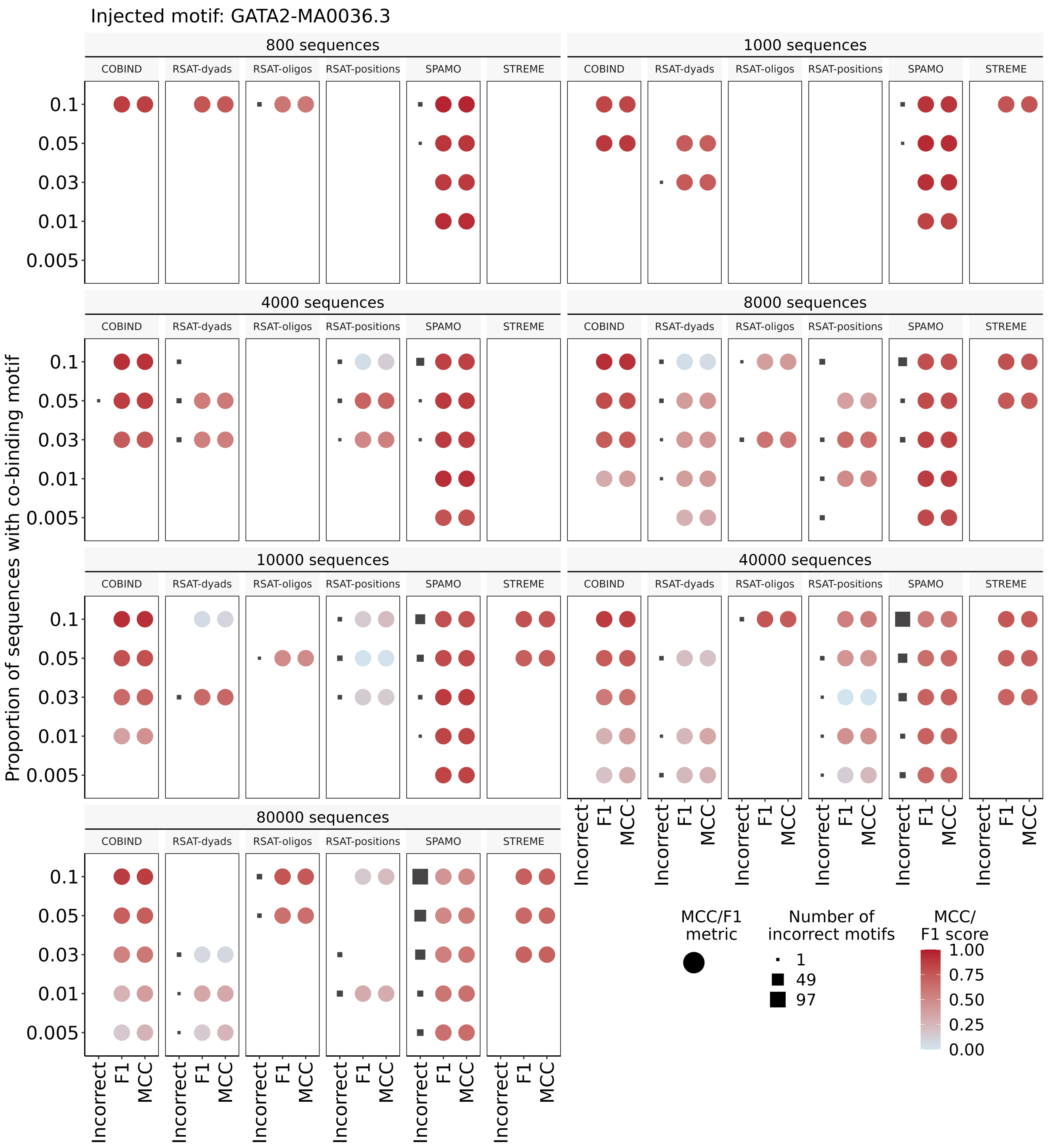

Figure S21. **Comparisons between COBIND and other tools on simulated data.** Same as Figure S18, but with instances of the JASPAR MA0036.3 TF binding profile for GATA2.

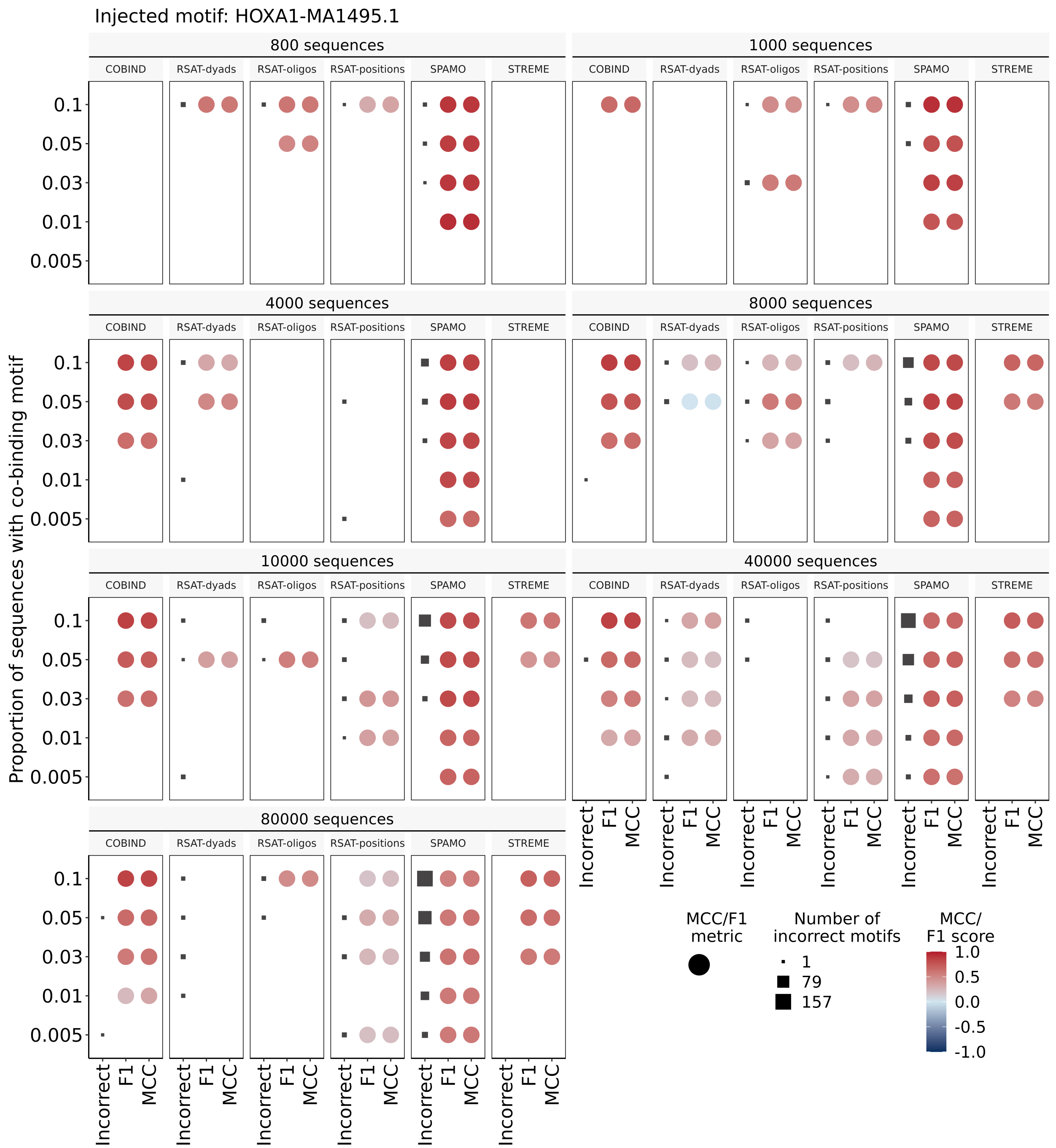

Figure S22. **Comparisons between COBIND and other tools on simulated data.** Same as Figure S18, but with instances of the JASPAR MA1495.1 TF binding profile for HOXA1.

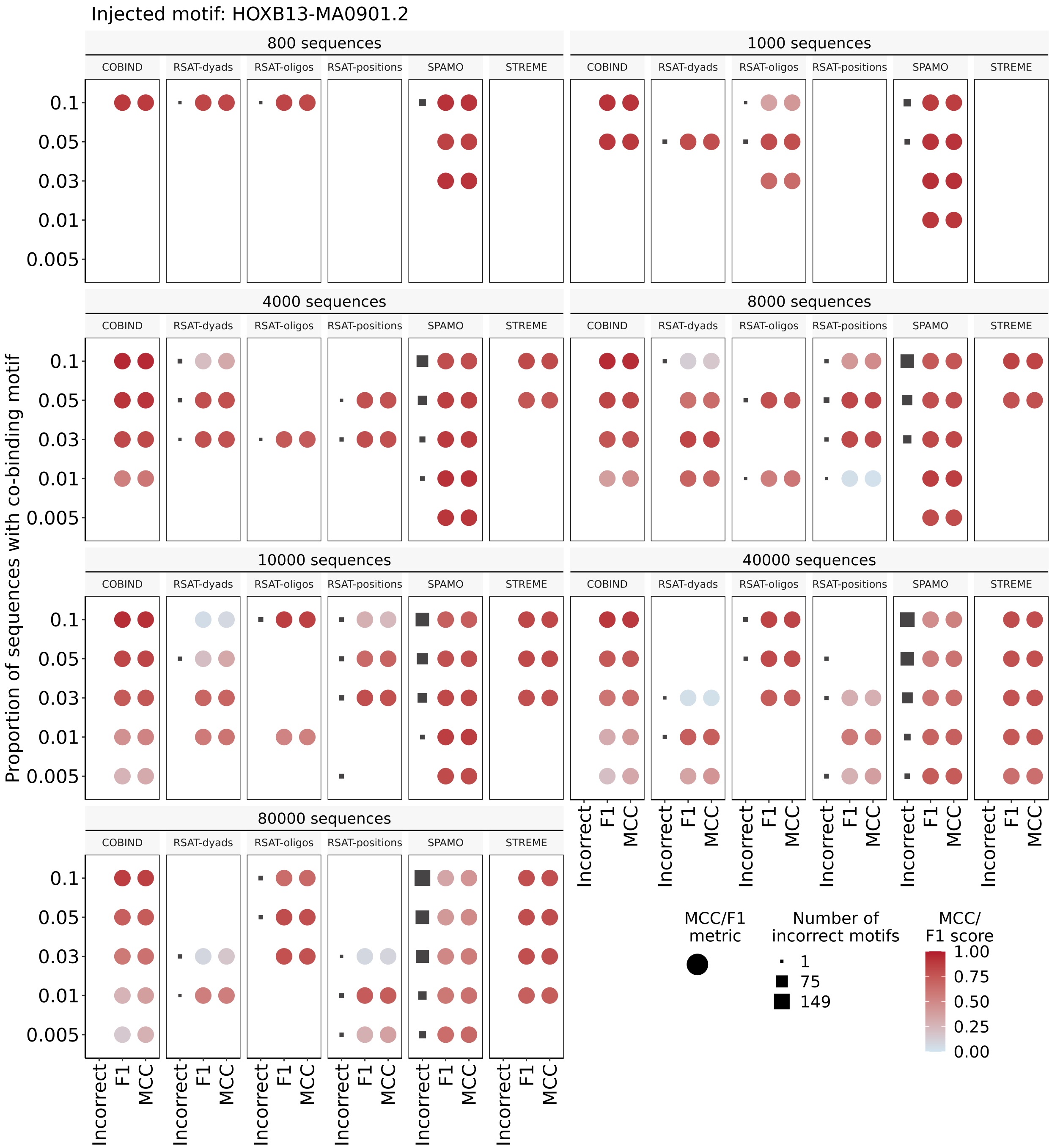

Figure S23. **Comparisons between COBIND and other tools on simulated data.** Same as Figure S18, but with instances of the JASPAR MA0901.2 TF binding profile for HOXB13.

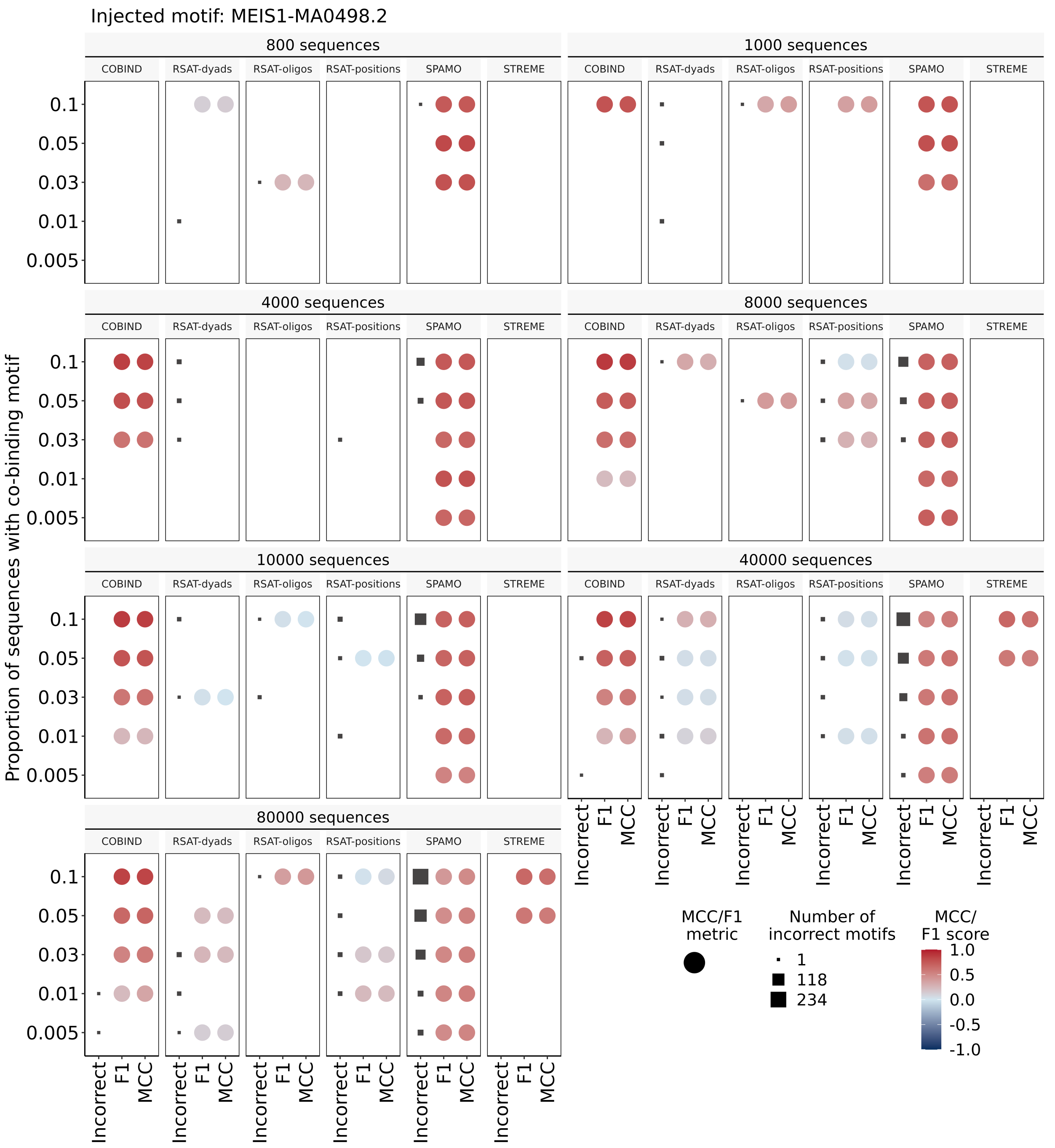

Figure S24. **Comparisons between COBIND and other tools on simulated data.** Same as Figure S18, but with instances of the JASPAR MA0498.2 TF binding profile for MEIS1.

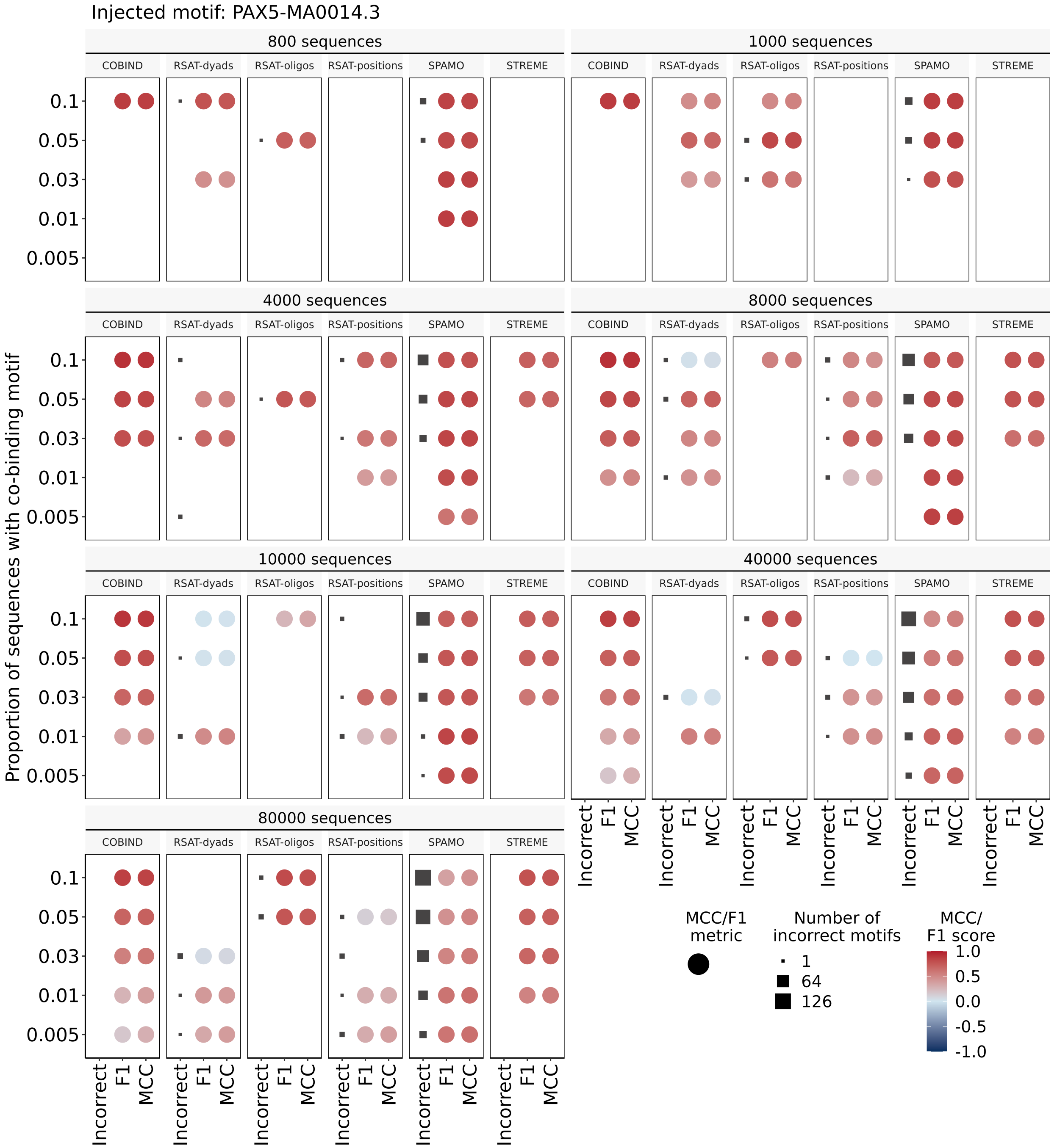

Figure S25. **Comparisons between COBIND and other tools on simulated data.** Same as Figure S18, but with instances of the JASPAR MA0014.3 TF binding profile for PAX5.

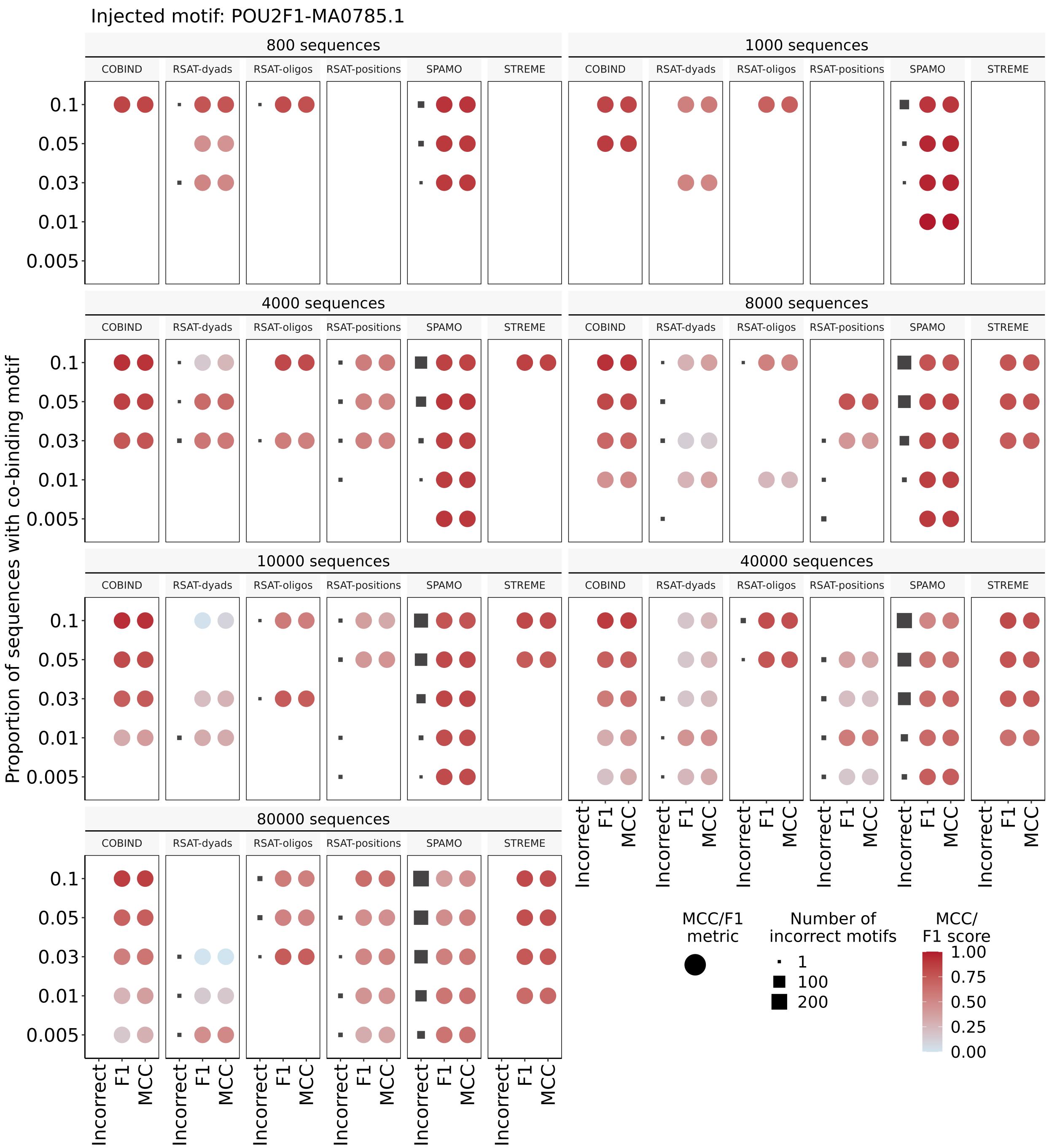

Figure S26. **Comparisons between COBIND and other tools on simulated data.** Same as Figure S18, but with instances of the JASPAR MA0785.1 TF binding profile for POU2F1.

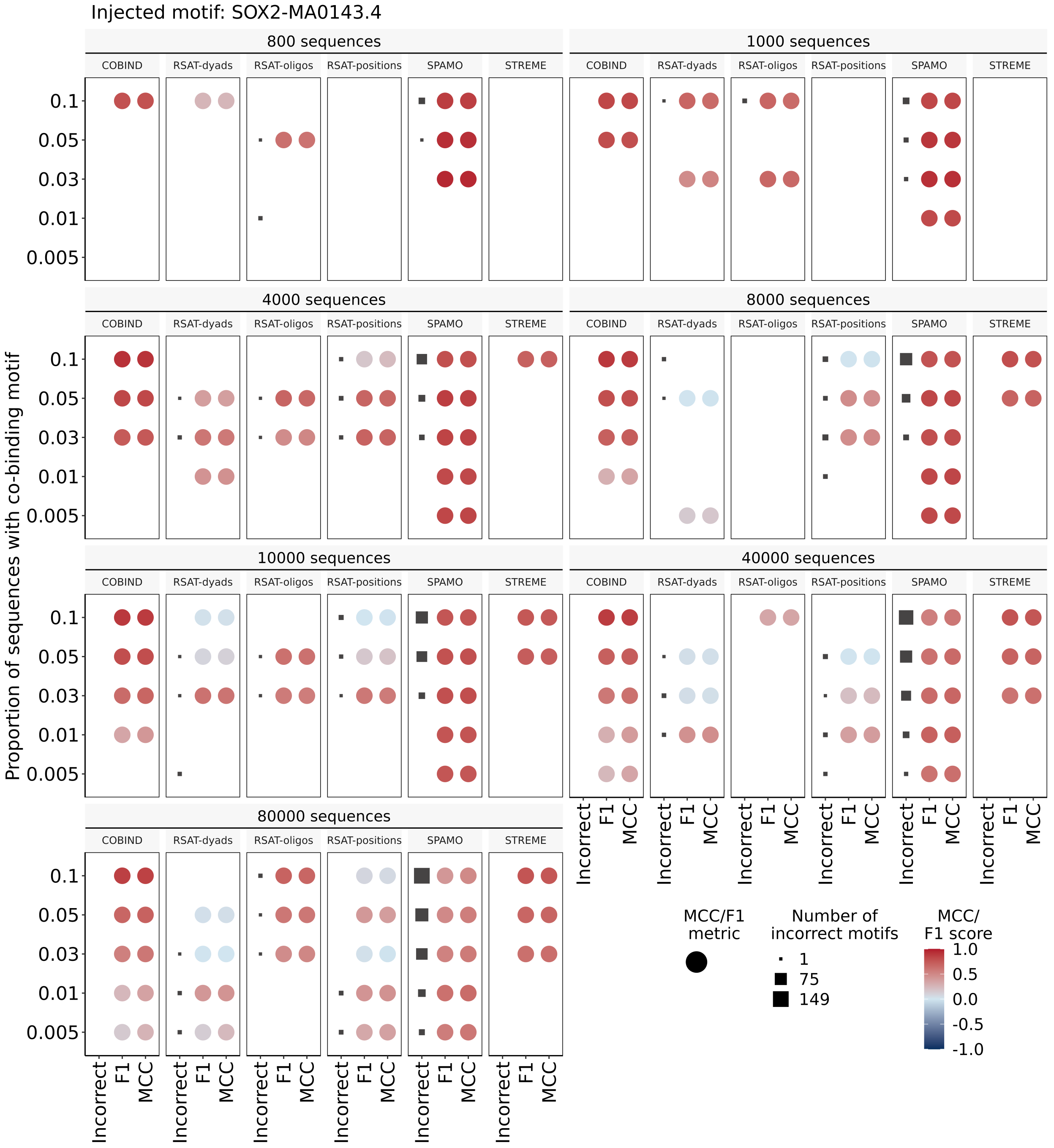

Figure S27. **Comparisons between COBIND and other tools on simulated data.** Same as Figure S18, but with instances of the JASPAR MA0143.4 TF binding profile for SOX2.

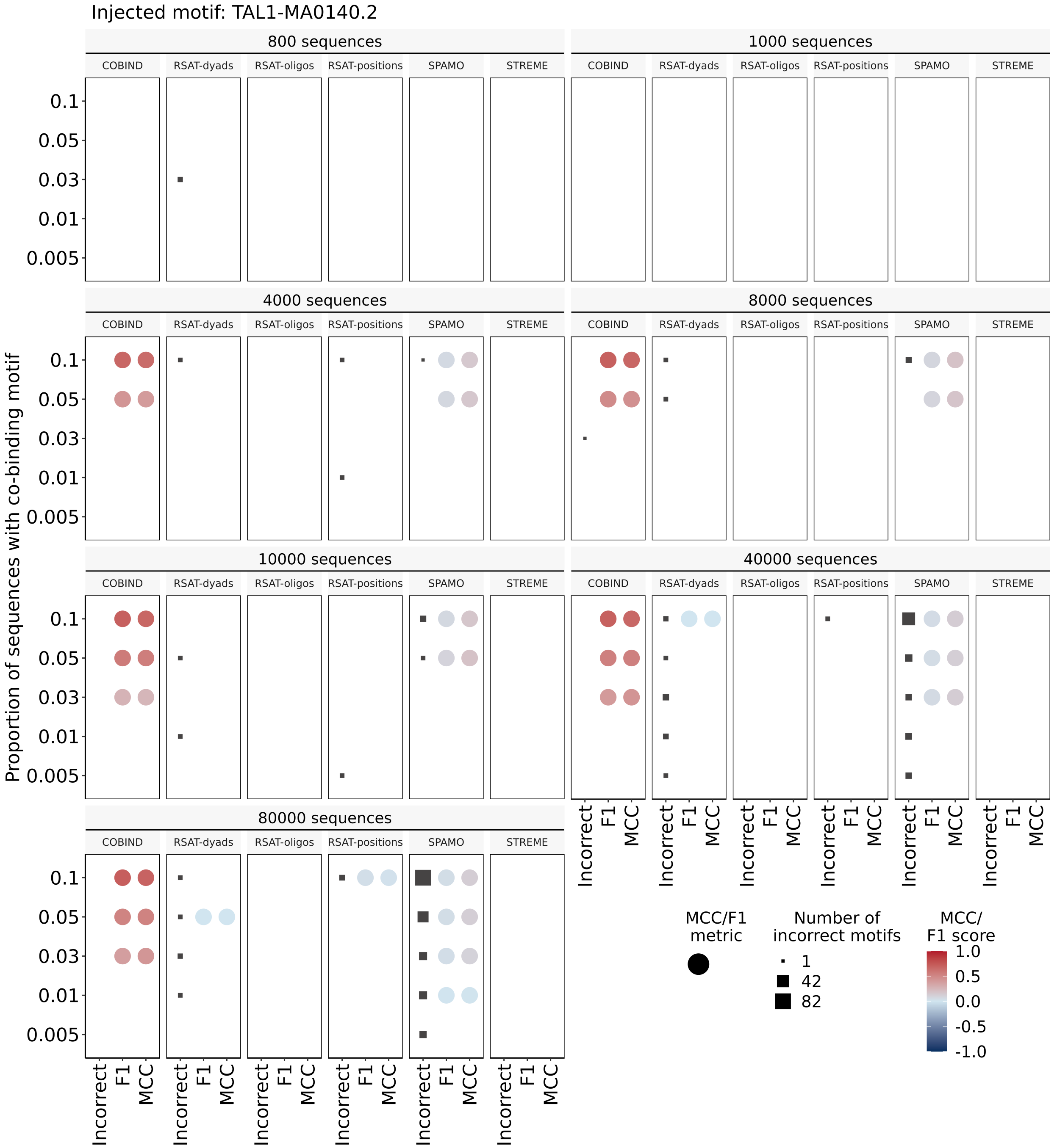

Figure S28. **Comparisons between COBIND and other tools on simulated data.** Same as Figure S18, but with instances of the JASPAR MA0140.2 TF binding profile for TAL1.

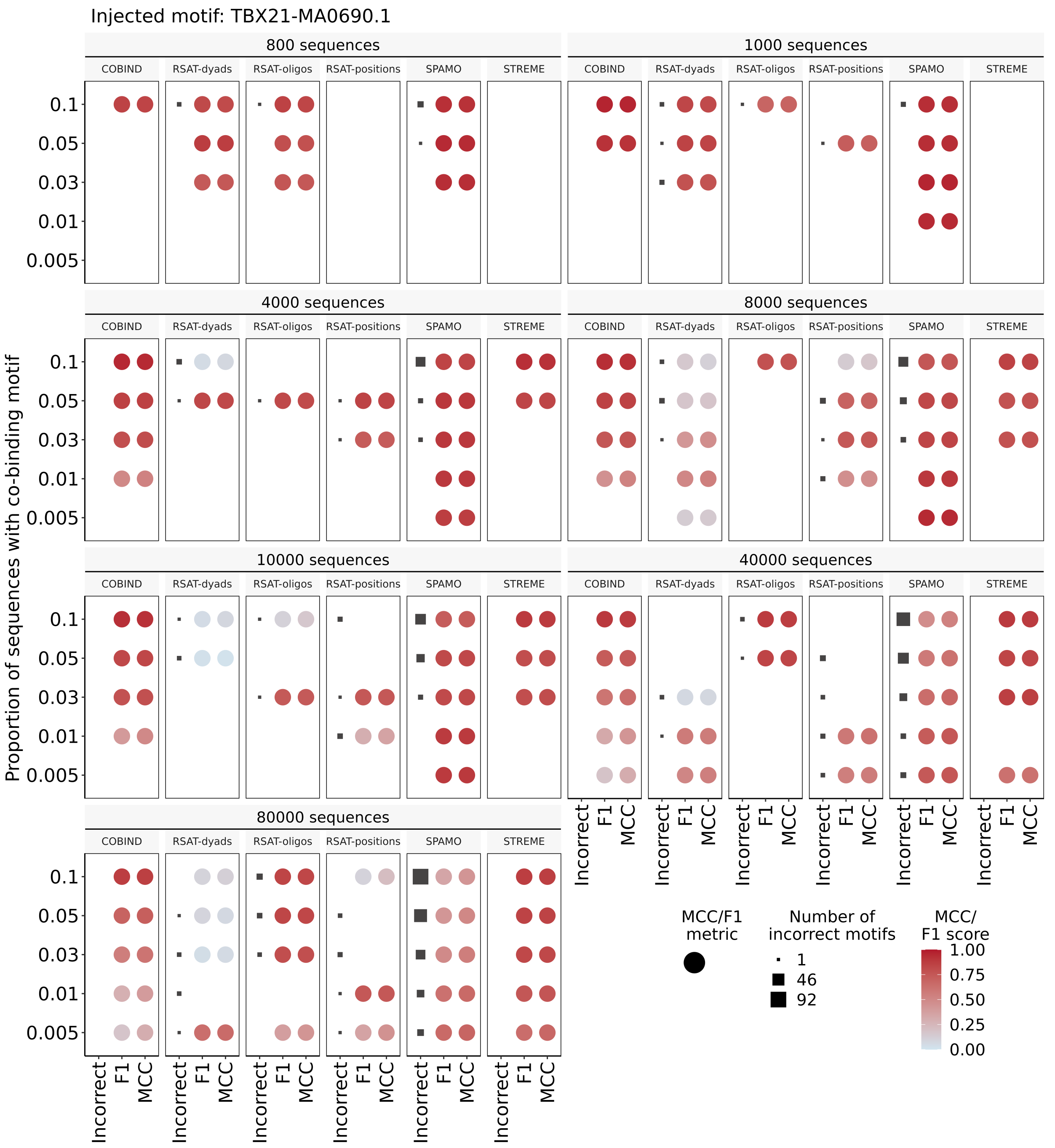

Figure S29. **Comparisons between COBIND and other tools on simulated data.** Same as Figure S18, but with instances of the JASPAR MA0690.1 TF binding profile for TBX21.

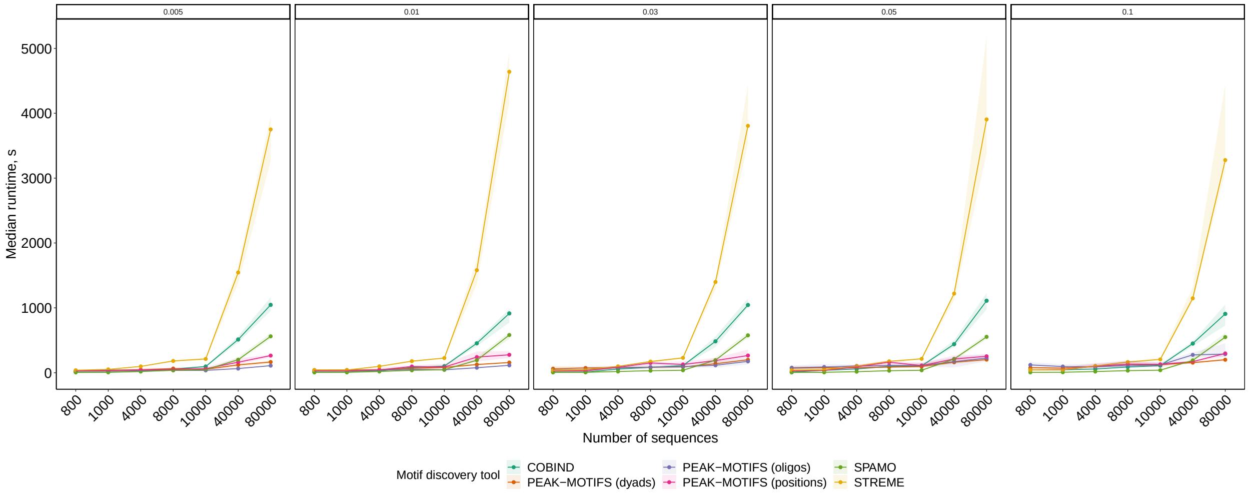

Figure S30. **Run time comparison on simulated data.** The figure reports the running time (y-axis) of the different tools (see legend) when applied to different numbers of sequences (columns) with distinct proportions of injected motif instances (see facets).

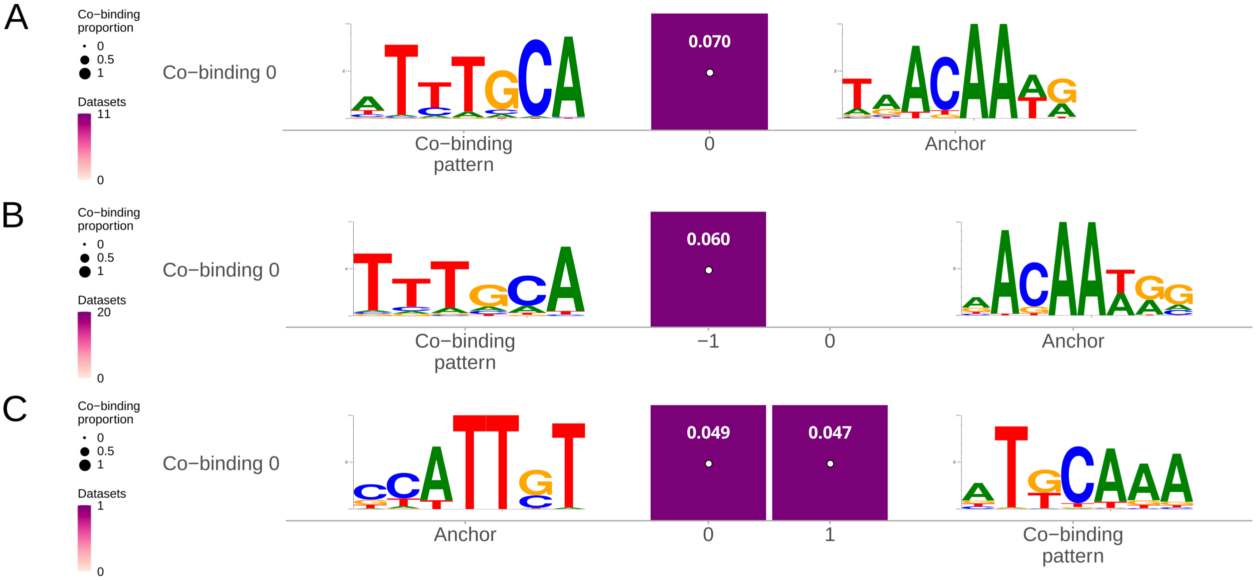

Figure S31. **Applying COBIND to SOX2 and SOX17 *Homo sapiens* and *Mus musculus* TFBS datasets.** POU5F1 partners with SOX2 to bind its canonical motif in *H. sapiens* (**A**) and *M. musculus* (**B**). In contrast, POU5F1 associates with SOX17 to bind a compressed motif in *M. musculus* (**C**).

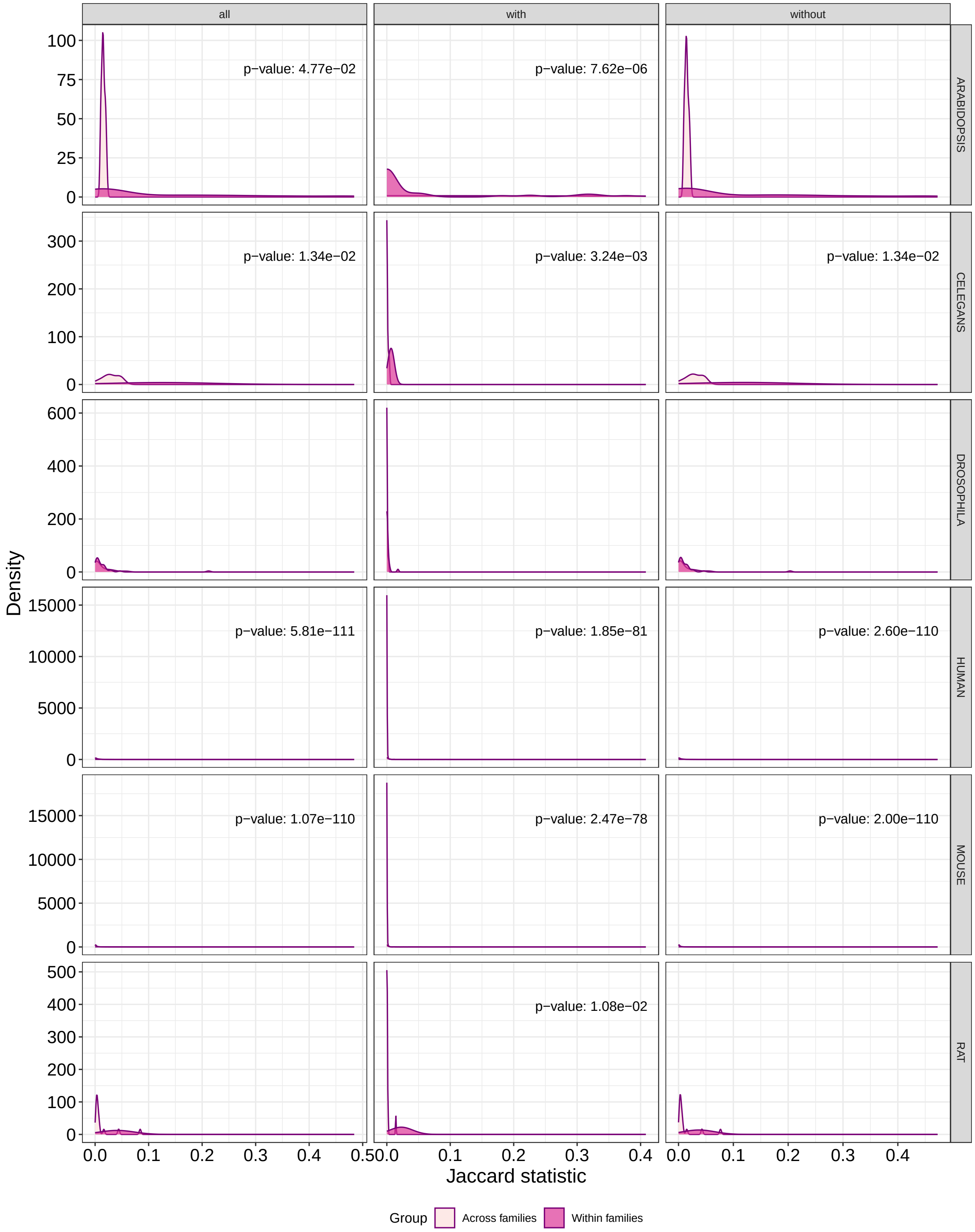

Figure S32. **Jaccard index distributions when comparing anchor sites for pairs of TFs.** We considered anchors sites for pairs of TFs with DBDs from the same or different structural family (dark pink, "within families" for same family; light pink, "across families" for different families). We compared

the two groups using Mann-Whitney U test; significant p-values (< 0.05) are indicated in the facets. The distributions of Jaccard index values are provided for different species and three types of regions: all anchor sites ("all"), only sites with predicted co-binding patterns ("with"), and sites without predicted co-binding patterns ("without").

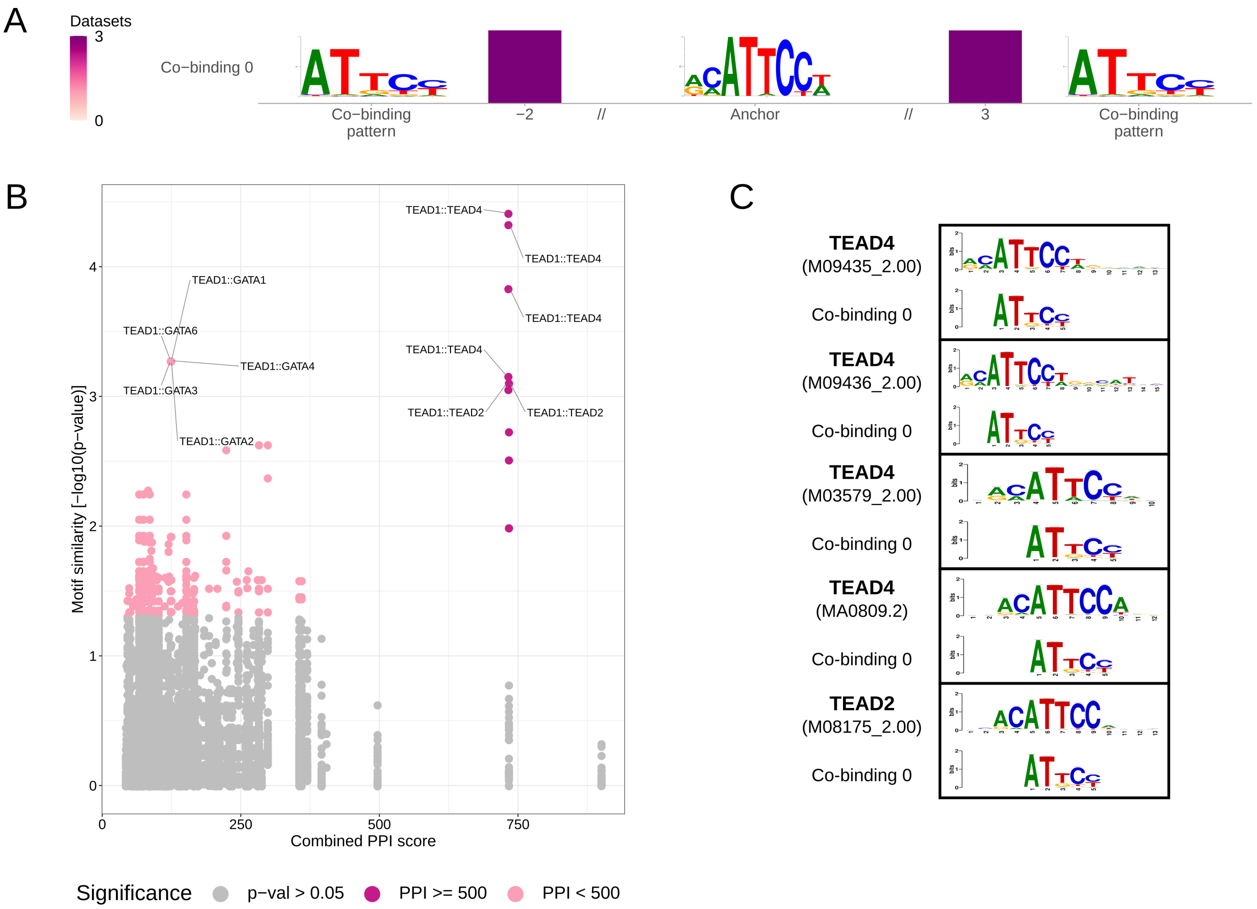

Figure S33. **TEAD1 COBIND results.** COBIND predicts TEAD1 to homodimerize; the predicted motif is similar to the known binding motif associated with TEAD TFs, and TEAD1::TEAD1 obtains the highest combined PPI score. (**A**) The same co-binding pattern is identified upstream (3 bp spacing) and downstream (2 bp spacing) of the anchor sites in the human and mouse genomes. (**B**) The co-binding motif has the highest similarity and PPI score (among mouse proteins) with TEAD proteins. (**C**) The Discovered co-binding motif matches best with TEAD4 and TEAD2 TF binding profiles.

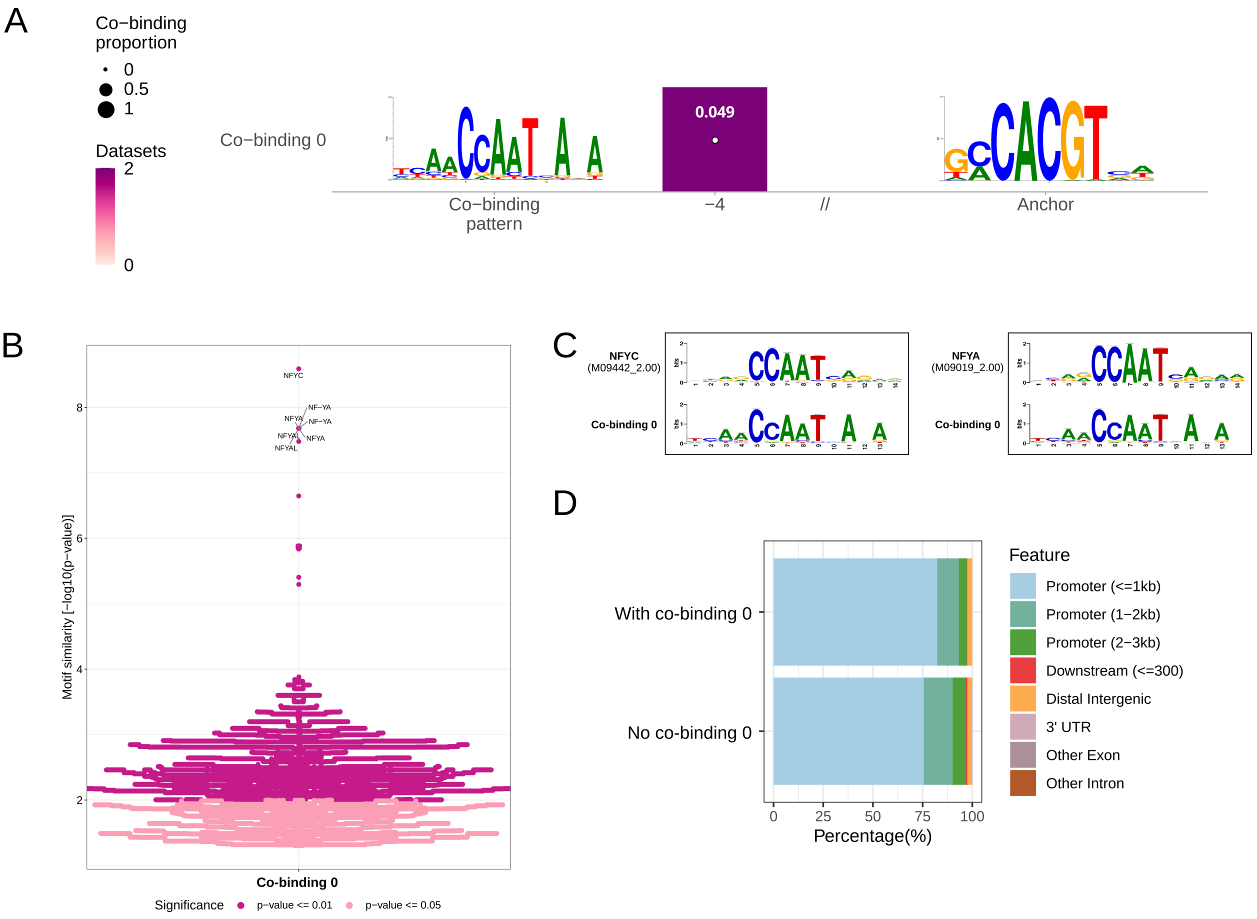

Figure S34. **HY5 COBIND results.** (**A**) COBIND predicts a co-binding pattern 4 bp upstream of the anchor HY5 sites in the *Arabidopsis thaliana* genome. (**B**) The co-binding pattern is similar to the motif bound by NF-Y family proteins. (**C**) The top matching motifs are associatedwith the NF-YC and NF-YA binding profiles, but they are not known to interact physically with HY5 from the PPI data. (**D**) Most regions predicted to contain the co-binding pattern localize in promoter regions.

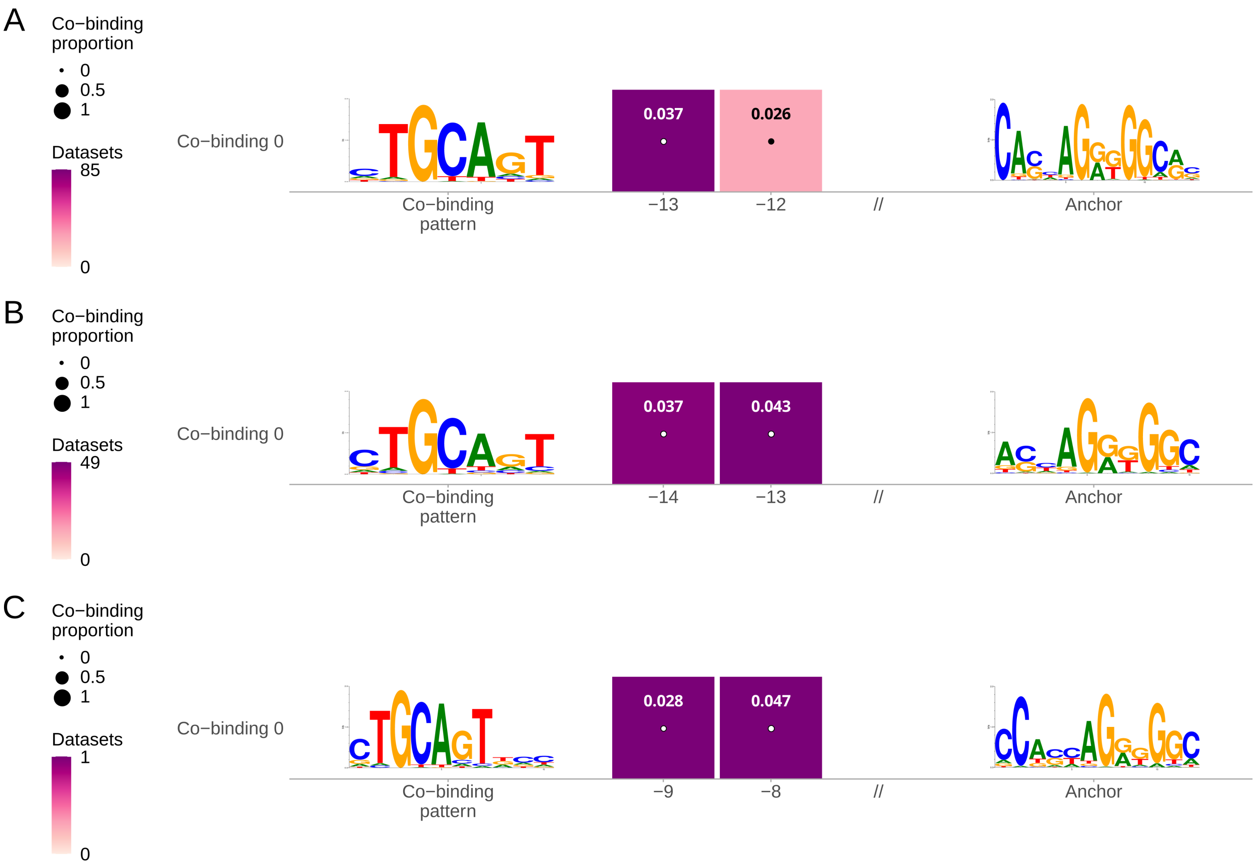

Figure S35. **CTCF COBIND results across species.** The extended motif associated with CTCF was predicted by COBIND with two different spacing patterns in datasets from mouse(**A**), human (**B**), and Zebrafish (**C**).

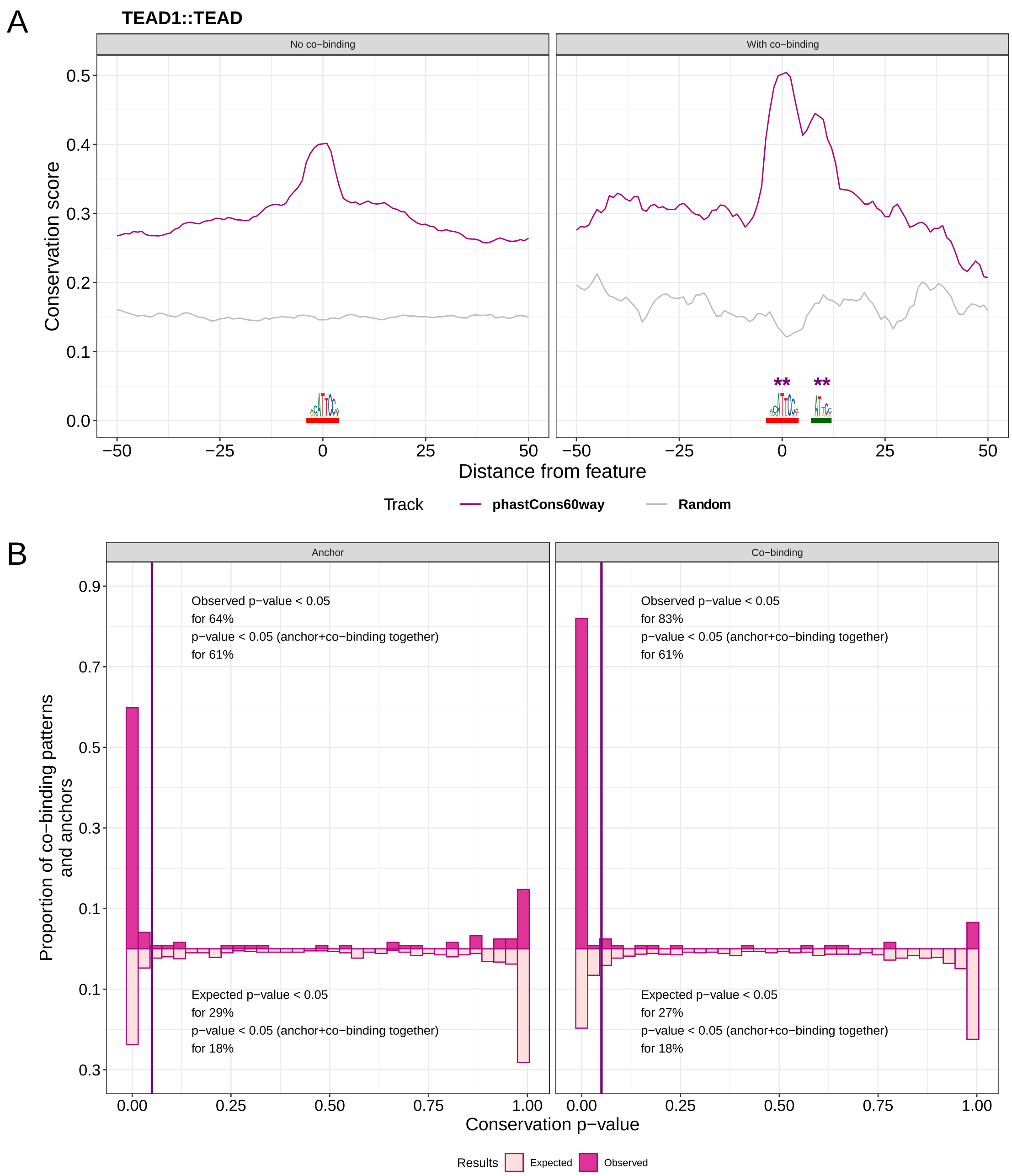

Figure S36. **Analysis of the evolutionary conservation of co-binding patterns discovered by COBIND in the mouse genome.** (**A**) Comparison between vertebrate evolutionary conservation of genomic regions where COBIND predicted a co-binding pattern (right) or not (left). The purple lines provide the mean conservation score across the regions considered. The grey lines provide the mean conservation score across the same number of random genomic regions in the human genome. ** indicates a Wilcoxon test p-value < 0.001. The anchor TEAD1 anchor motif is underlined in red, and the co-binding motif is underlined in green. (**B**) We compared the evolutionary conservation scores at the anchors' motif locations in genomic regions harboring a co-binding pattern or not. The left panel represents the histogram of the proportion of datasets with the anchor sites harboring higher evolutionary conservation in co-bound regions than in other regions. The right panel represents the corresponding histogram when comparing genomic locations of the co-binding motif. Dark pink represents observed values, and light pink the expected values computed from random assignment of genomic regions predicted as co-bound or not.

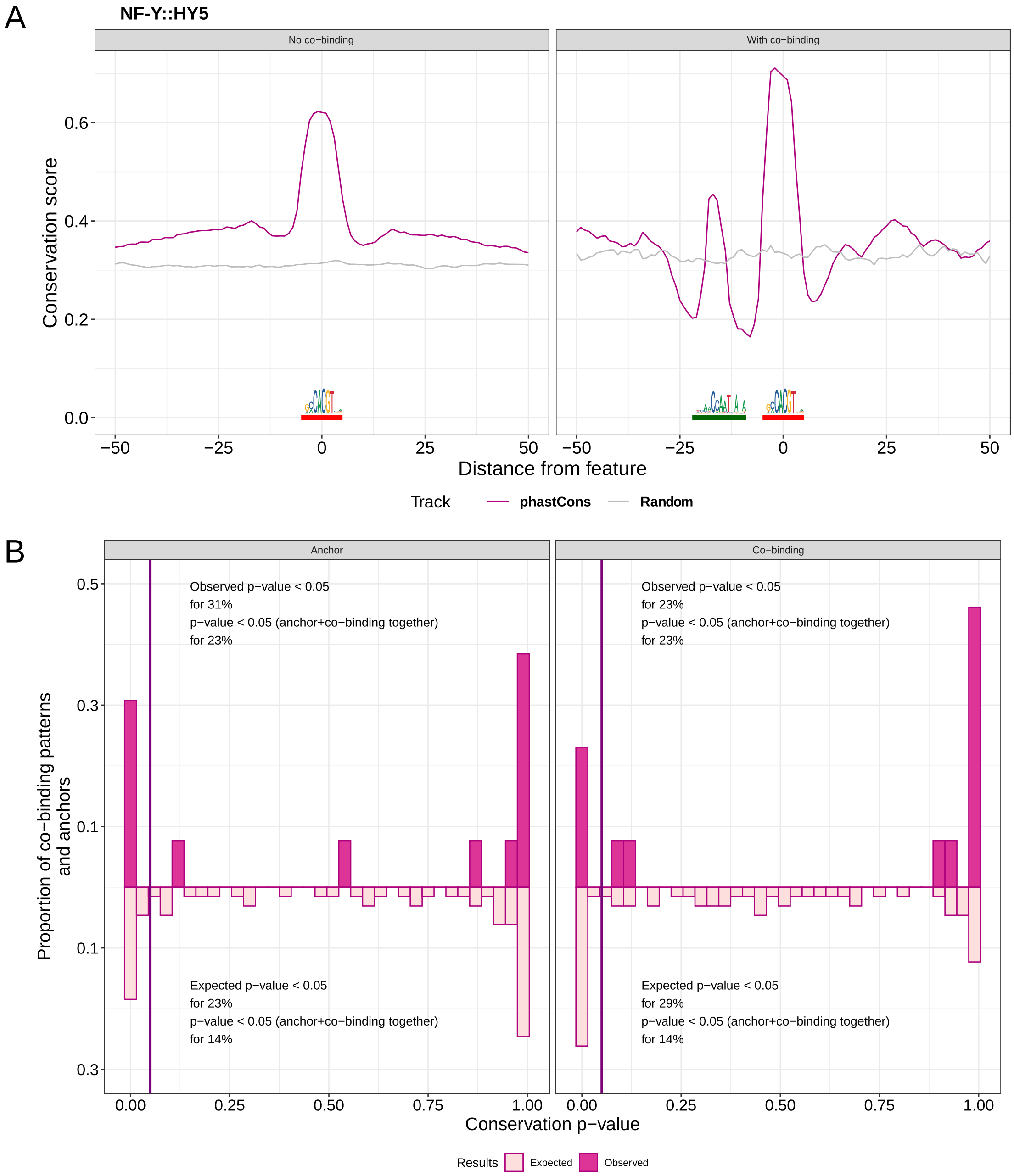

Figure S37. **Analysis of the evolutionary conservation of co-binding patterns discovered by COBIND in the *A. thaliana* genome.** (**A**) Comparison between plant evolutionary conservation of genomic regions where COBIND predicted a co-binding pattern (right) or not (left). The purple lines provide the mean conservation score across the regions considered. The grey lines provide the mean conservation score across the same number of random genomic regions in the human genome. ** indicates a Wilcoxon test p-value < 0.001. The anchor HY5 anchor motif is underlined in red, and the co-binding motif is underlined in green. (**B**) We compared the evolutionary conservation scores at the anchors' motif locations in genomic regions harboring a co-binding pattern or not. The left panel represents the histogram of the proportion of datasets with the anchor sites harboring higher evolutionary conservation in co-bound regions than in other regions. The right panel represents the corresponding histogram when comparing genomic locations of the co-binding motif. Dark pink represents observed values, and light pink the expected values computed from random assignment of genomic regions predicted as co-bound or not.

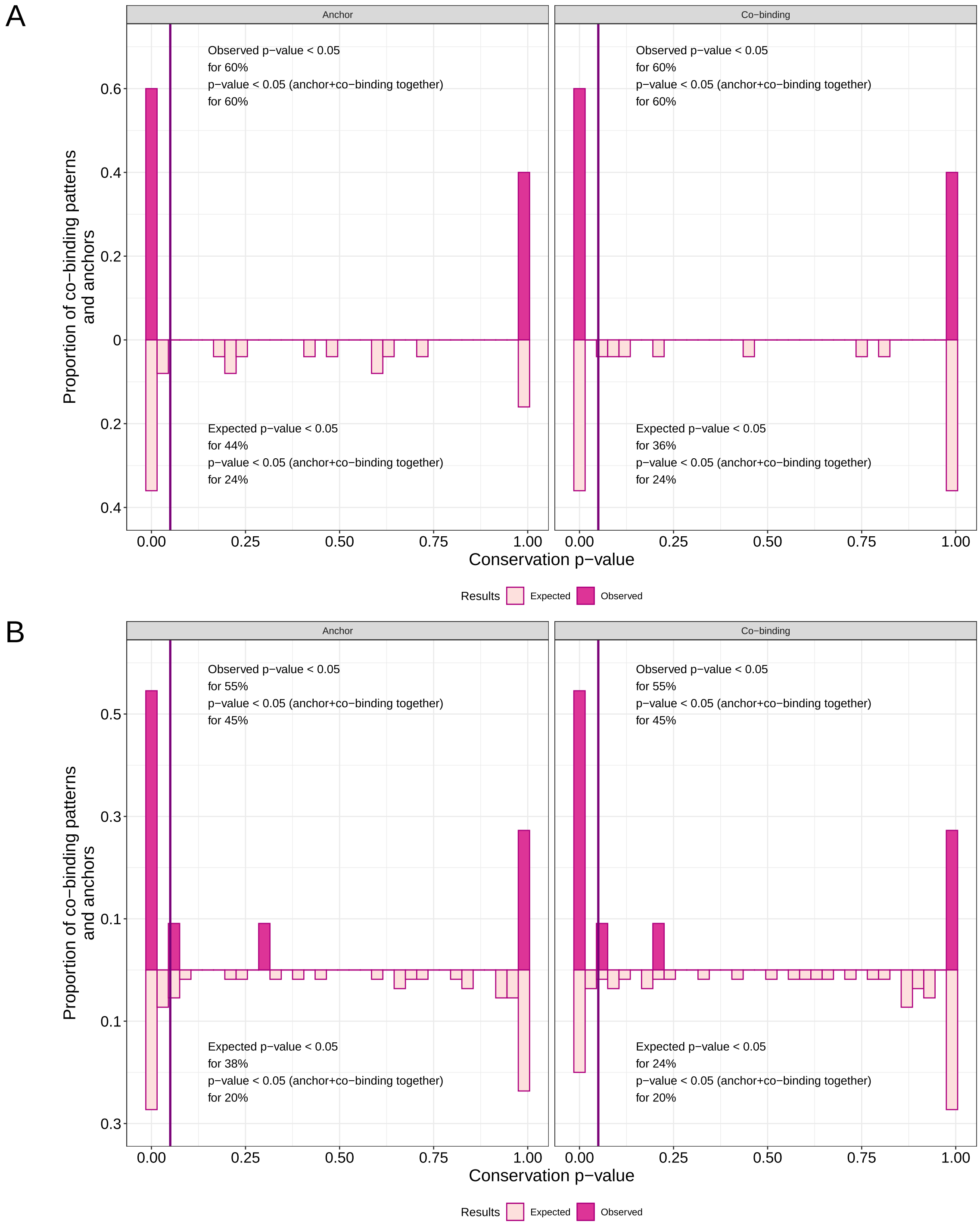

Figure S38. **Analysis of the evolutionary conservation of co-binding patterns in the *C. elegans* (A) and *D. melanogaster* (B) genomes discovered by COBIND.** We compared the evolutionary conservation scores at the anchors' motif locations in genomic regions harboring a co-binding pattern or not. The left panels represent the histogram of the proportion of datasets with the anchor sites harboring higher evolutionary conservation in co-bound regions than in other regions. The right panels represent the corresponding histogram when comparing genomic locations of the co-binding motif. Dark pink represents observed values, and light pink the expected values computed from random assignment of genomic regions predicted as co-bound or not.

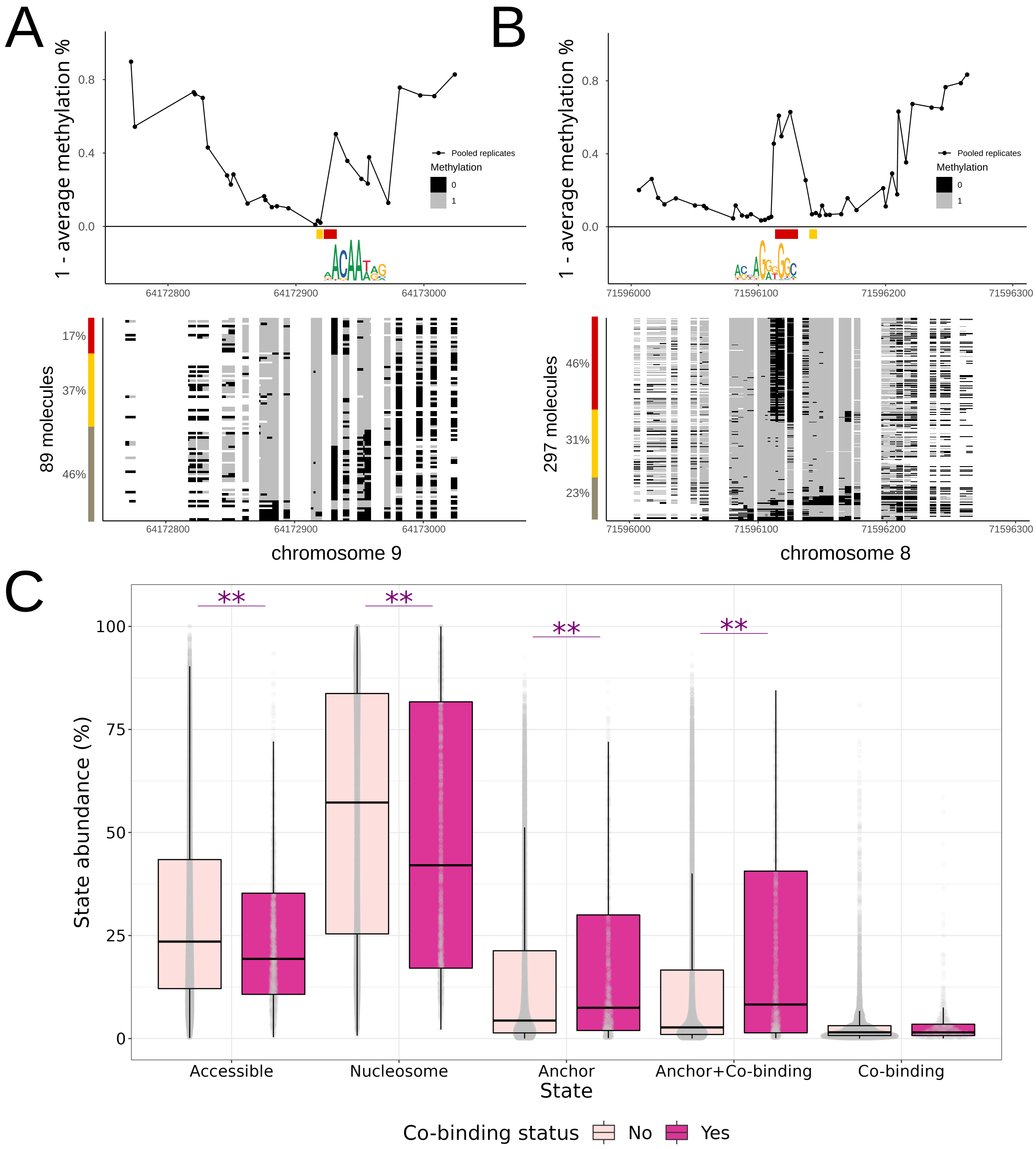

Figure S39. **Single-molecule footprinting analysis.** (**A-B**) Single-locus examples of regions where no co-binding pattern was predicted for SOX2 (**A**) and CTCF (**B**) anchors. Line plots in the top panels represent the average methylation levels. We provide logos and locations of the anchor motifs (red) and the relative locations of co-binding patterns that were predicted in the rest of the regions (yellow). Stacks of sorted single molecules are visualised in the lower panels (light grey indicates methylated Cs, accessible positions; black - unmethylated Cs, protected). The y-axis corresponds to the total number of molecules. Coloured bars and percentages show the proportion of molecules in each state (colours corresponding to Figure 8A). (**C**) Boxplots of the distribution of co-occupied molecules ("Anchor + Co-binding") overlapping regions with (dark pink) and without (light pink) a predicted co-binding pattern by COBIND when considering different anchor TFBSs. ** represents a Wilcoxon test p-value < 0.05.

SUPPLEMENTARY TABLES

Table S1. **Parameters used when running the external tools within the COBIND pipeline.**

Tool/parameter name	Purpose	Value
	Default COBIND parameters	
Flank length n (base pairs)	Generating regions flanking the input anchors	`30`
Number of components k	A vector of k number of components to run NMF with	`[3, 6]`
Gini coefficient	A value from 0 to 1 used to filter discovered motifs	`0.5`
RSAT matrix-clustering	Clustering of discovered co-binder patterns	`-v 2 -calc sum -metric_build_tree cor -lth w 3 -lth cor 0.7 -lth Ncor 0.4`
	Clustering of associated anchor motifs	`-v 2 -calc sum -metric_build_tree cor -lth w 3 -lth cor 0.75 -lth Ncor 0.55`
	Motif comparison	
Tomtom v5.5.4	Comparing *de novo* discovered motifs with inserted true motif (synthetic data analysis) or with known motif databases (TF inference through PPI analysis)	`-dist kullback -motif-pseudo 0.1 -min-overlap 1 -thresh 1 -eps -no-ssc`

Table S2. **UniBind 2021 robust collection data used in this study.**

Species name	Genome assembly	Number of files with TFBSs	Number of unique TFs	Number of unique TF motifs	Number of unique cell lines/tissues	Number of unique biological conditions
Arabidopsis thaliana	TAIR10	59	27	27	17	33
Caenorhabditis elegans	WBcel235	59	19	19	11	41
Danio rerio	GRCz11	5	4	4	3	3
Drosophila melanogaster	dm6	73	16	16	24	36
Homo sapiens	hg38	2,657	247	268	363	801
Mus musculus	mm10	2,809	260	275	483	1,049
Rattus norvegicus	Rnor 6.0	37	12	12	12	20

***Table S3.* Overview of tools for motif discovery and/or the investigation of TF cooperation.**

	**COBIND**	**STREME**	**RSAT peak-motifs** *(oligos, positions, dyads)*	**SpaMo**	**TACO**	**iTFs**	**MCOT**
Year	**2024**	*2021*	*2011*	*2011*	*2014*	*2013*	*2019*
PubMed ID	***This paper***	33760053	22156162, 22836136	21602262	24640962	23847101	31750523
Input data	Anchor regions (e. g., TFBSs)	Genomic sequences in fasta	Genomic sequences in fasta	Genomic sequences in fasta, known anchor motif, motif library	ChIP-seq or DNase-seq peaks, motif library	ChIP-seq peaks, motif library, TF pairs with known PPI	ChIP-seq peaks in fasta, known anchor motif, motif library
Anchor/ Primary motif* & *Discovery of co-binding motifs	Anchor regions is input, but motif is not needed. Co-binding patterns are discovered *de novo* upstream and downstream of the input (each dataset individually)	No co-binding inferred. Motifs are searched and discovered *de novo*	No co-binding inferred. Motifs are searched and discovered *de novo* using global over-representation (oligos) or positional bias (positions) or searching for two over-represented oligos (dyads)	Anchor motif must be known and provided. Matches for co-binding motifs are searched in the motif library. Match consensus and sites for each match are returned	Searches for matches in the motif library. Anchor-co-binder pairs are statistically tested	Binding sites for TF-co-binder pairs are predicted. Orientation and spacing bias is statistically tested	Co-binding partners are searched in the provided motif library
Number of datasets	Individual datasets analyzed, summarized over groups of datasets	Motifs are discovered in individual datasets	Individual datasets analyzed	Individual datasets analyzed	More than one dataset is required	Individual datasets analyzed	Individual datasets analyzed

(Continued in the next page)

	**COBIND**	**STREME**	**RSAT peak-motifs** *(oligos, positions, dyads)*	**SpaMo**	**TACO**	**iTFs**	**MCOT**
Output	Summary of spacings and locations of co-binders relative to the anchor motif together with sets of genomic regions with/without co-binders	Discovered motif logos and matrices together with discovery summary and combined discovered matrix	Discovered motif logos and matrices. Discovery summary with indicated motif positions in the set of sequences	Sequences where each motif from the library was found, results summary in html and tab-separated table formats	Dimer motifs, summary tables and genomic locations of cooperation events	Summary table	Anchor-co-binder pairs, spacing, regions
Summary of spacers and/or discovery of overlapping motifs	Spacers are summarized and if overlap resulted in partial motif, such motif discovery is possible	-	Positional distribution is indicated, but post-processing would be needed to interpret it as a spacer. For dyads additional interpretation would be needed to distinguish anchor and co-binder	Spacer	Spacer and overlap	Spacer (categorized into ranges)	Spacer and overlap. A range of possible spacers should be defined by the user, default minimal 0 bp, maximum 29 bp
Scalability	Parallelization over datasets and within the same dataset for motif discovery is possible through Snakemake	One command call for one dataset	One command call for one dataset	One command call for one dataset	Unknown	Unknown	One command call for one dataset

(Continued in the next page)

	**COBIND**	**STREME**	**RSAT peak-motifs** *(oligos, positions, dyads)*	**SpaMo**	**TACO**	**iTFs**	**MCOT**
Applications	*De novo* discovery of TF co-binding motifs; discovery of extended TF motifs; summary of discovery, including spacings between reference and discovered motifs; other sequence pattern discoveries	*De novo* TF motif discovery	*De novo* TF motif discovery; differential motif discovery between two conditions	Identification of co-binding TFs and TF cooperativity analysis	Identification of co-binding TF pairs in DNase-seq and ChIP-seq datasets	Identification of co-binding TFs and TF cooperativity analysis	Identification of the co-binder motifs, analysis and summary of TF cooperativity
Availability	Command line	Web server, command line	Web server, command line	Web server, command line	Command line	Web server unavailable as of 2024	Web server, command line
Comments	Minimum of 1000 regions input regions is recommended	The tool does not perform any specific co-binding related analysis	The tool does not perform any specific co-binding related analysis	Highly dependent on the used motif library	Better predictions are achieved when there is cell type specific information	The tool was not included in the analysis due to code unavailability	The tool was not included in the analysis due to segmentation fault errors (tool not tested by developers with datasets with more than 10,000 sequences)

TF - transcription factor

TFBSs - transcription factor binding sites

Table S4. **Cell types and sources where CTCF extended motif was found.**

Species	Source	Cell type
Danio rerio	embryonic cell	10 hpf embryo (bud stage)
Homo sapiens	adrenal gland	adrenal gland
	blood	lymphoblastoid cells
	cell line	226LDM (normal breast luminal cells)
		A549 (lung carcinoma)
		BCBL-1 (B-cell lymphoma)
		BL41(Burkitt lymphoma)
		GM12801 (male B-cells)
		GM12873 (female B-cells)
		GM18505 (female B-cells Lymphoblastoid Cell Lines)
		GM18526 (B − cells Lymphoblastoid Cell Lines)
		HSMM (skeletal muscle myoblasts)
		HUES64 (embryonic stem cells)
		HUVEC-C (HUVEC, umbilical vein endothelial cells)
		HeLa Kyoto (cervical adenocarcinoma)
		K562 (myelogenous leukemia)
		MCF7 (Invasive ductal breast carcinoma)
		PC9 (lung adenocarcinoma)
		RCC 7860 (Renal cell carcinoma)
		SK-N-SH (neuroblastoma)
		T47D (invasive ductal carcinoma)
		THP-1 (acute monocytic leukemia)
	colon	sigmoid colon
	esophagus	esophagus squamous epithelium
	heart	human cardiac myocytes
	immune system	MDM(monocyte derived macrophages)
	kidney	kidney
	nervous system	bipolar neuron
	other	neutrophils
	prostate	prostate gland
	skin	epidermal keratinocytes
		keratinocytes
	spinal cord	human astrocytes-spinal cord
	spleen	spleen
	stem cell	IPSC-derived bipolar neuron
	stomach	stomach
Mus musculus	bone marrow	myeloid malignancies
	brain	cerebral cortex
		olfactory bulb
	breast	mammary gland
	cell line	3T3-L1-derived preadipocytes
		C2C12 (myoblasts)
		CH12.LX (Mouse lymphoma)
		ECOMG (myeloid progenitor cell line)

(Continued in the next page)

Species	Source	Cell type
Mus musculus	cell line	ECOMG-derived differentiated neutrophils
		EL4 (lymphoma)
		G1E (embryonic stem cells)
		G4 (embryonic stem cells)
		WEHI-231 (lymphoma)
		clonal MEF
	cerebellum	cerebellum
	embryo	E13.5 embryonic fibroblasts
		E13.5 forelimb
		E13.5 hindlimb
		E14.5 brain-derived neural progenitor cells
		E14.5 heart
		E14.5 limb
		E14.5 retina
		E17.5 retina
		P0 retina
		P10 retina
		P14 retina
		P21 retina
		P3 retina
		P7 retina
		embryonic fibroblasts
	eye	retina
	heart	cardiac myocytes
	immune system	B-cells
		Th2-cells
		dendritic cells
		flow sorted pre-B cells
		pro-B cells
	kidney	nephron progenitors
	liver	liver
	nervous system	medial ganglionic eminence
		olfactory neurons
	other	NRL-GFP sorted Rod cell postnatal day 21
		NRL-GFP sorted Rod cell postnatal day 6
		Treg (regulatory T cells from lymph nodes and spleen)
		fibroblasts
		in-vitro differentiated cortical neurons (CN)
		in-vitro differentiated neural progenitor cells (NPC)
		limb
		primary cells (short term)
	placenta	placenta
	skin	dermal fibroblasts
	stem cell	differentiated embryonic stem cells
		embryonic stem cells
		induced pluripotent stem cells
	testis	spermatocytes
	thymus	thymus
