# Supplementary material for: Identification of transcription factor co-binding patterns with non-negative matrix factorization
Source: Nucleic Acids Res. 2024 Sep 1;52(18):e85. doi: 10.1093/nar/gkae743 (PMC11472169; doi:10.1093/nar/gkae743)
Supplement: gkae743_Supplemental_Files [file gkae743_supplemental_files.zip › additional_file_2.pdf]

IDENTIFICATION OF TRANSCRIPTION FACTOR CO-BINDING PATTERNS WITH NON-NEGATIVE MATRIX FACTORIZATION

Ieva Rauluseviciute[1] Timothée Launay[1] Guido Barzaghi[2,3]
Sarvesh Nikumbh[4,5] Boris Lenhard[4,5] Arnaud Regis Krebs[2]

Jaime A. Castro-Mondragon[1] Anthony Mathelier[1,6,7,*]

[1] Centre for Molecular Medicine Norway (NCMM), Nordic EMBL Partnership, University of Oslo, 0318 Oslo, Norway
[2] European Molecular Biology Laboratory (EMBL), Genome Biology Unit, Meyerhofstraße 1, 69117 Heidelberg, Germany
[3] Collaboration for Joint Ph.D. degree between EMBL and Heidelberg University, Heidelberg, Germany
[4] MRC London Institute of Medical Sciences, Du Cane Road, London, W12 0NN, UK
[5] Institute of Clinical Sciences, Faculty of Medicine, Imperial College London, Hammersmith Hospital Campus, Du Cane Road, London, W12 0NN, UK
[6] Department of Medical Genetics, Institute of Clinical Medicine, University of Oslo and Oslo University Hospital, Oslo, Norway
[7] Center for Bioinformatics, Department of Informatics, Faculty of Mathematics and Natural Sciences, University of Oslo, Oslo, Norway

[*] Correspondence: Anthony Mathelier <anthony.mathelier@ncmm.uio.no>

ADDITIONAL FILE 2

MATERIALS & METHODS

Intron-exon border analysis

We downloaded intron coordinates for the human genome (*hg38* assembly) using the UCSC Table Browser (1). We then extracted the first two nucleotides of the exons flanking the introns. Depending on the direction of transcription, we defined these regions as donors or acceptors. We created two BED-formatted files - one for the donor and one for the acceptor sites, which we used as independent input files for COBIND. The pipeline was applied using default parameters.

COBIND analysis on randomly selected regions

We considered the 338 datasets for 44 TFs from the DHS analysis to perform control analyses with randomly selected regions. Specifically, we used the genomic mononucleotide distribution matched module of the Biasaway tool (2) for each dataset to randomly choose genomic regions of the same size and with the same %GC content. We ran COBIND on these control datasets to discover and summarize predicted co-binding patterns. This process was repeated five times per original dataset to ensure the representativity of the results (1,690 COBIND runs in total).

The co-binding patterns predicted by COBIND in the control datasets were compared to the ones predicted in the corresponding real dataset using Tomtom (3).

RESULTS

COBIND discovers expected splicing motifs

We ran COBIND on human donor and acceptor sites to see if COBIND discovers the expected splicing motifs (4). The acceptor motif consensus in different species is GT, and the consensus motif for donor sites is CAG (4, 5).

We anchored the COBIND analyses at the two exonic nucleotides flanking the introns. Thus, we analyzed the flanking regions upstream and downstream from the exon-intron borders. One flank is in the intron and another is in the exon (Materials and Methods above). COBIND revealed two patterns in the donor sites, where both have the consensus donor GT motif in around 7% of the input donor regions (Figure S40A). This consensus motif downstream of the anchor indicates the intronic flank. Moreover, the recovered anchor motif for the GTA motif (Co-binding pattern 0 in Figure S40A) contained high IC for AG, meaning that 4.4% of all input donor sites include AG as the last two exon nucleotides. When analyzing the acceptor sites, we found one DNA pattern corresponding to the consensus acceptor motif CAG in 1.4 % of input sequences (Figure S40B).

These analyses indicate that COBIND recovers the expected splicing signal motifs.

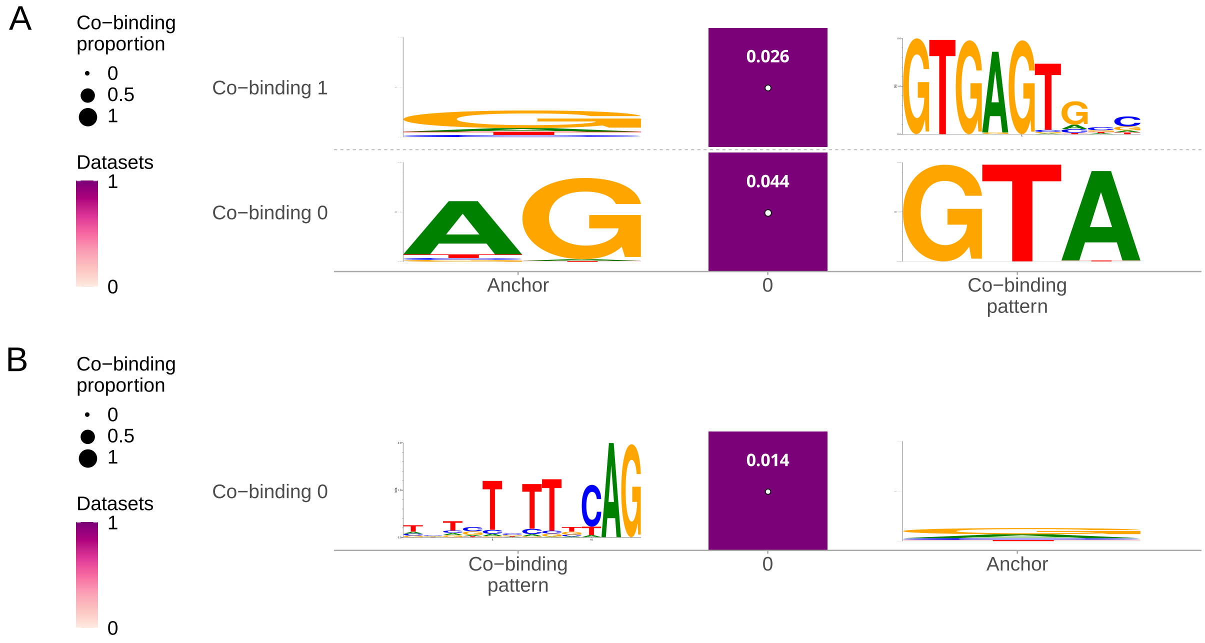

Figure S40. **COBIND recovers splicing signal motifs at the intron-exon boundaries for donor (A) and acceptor (B) sites.**

Co-binding motifs in control regions

Across all 1,690 (338 datasets times 5 runs) COBIND runs with control regions, 45 co-binding motifs were reported, all consisting of repeats of As or Ts, except one motif rich in Gs (see examples in Figure S41). Out of these 45 patterns, only 7 matched the ones found in the corresponding real datasets (Tomtom p-value $<$ 0.05; Figure S42), representing less than 0.5% of the total number of runs.

As highlighted in previous studies (6, 7), discovering A/T-rich patterns with low complexity is an established

issue for *de novo* motif discovery tools.

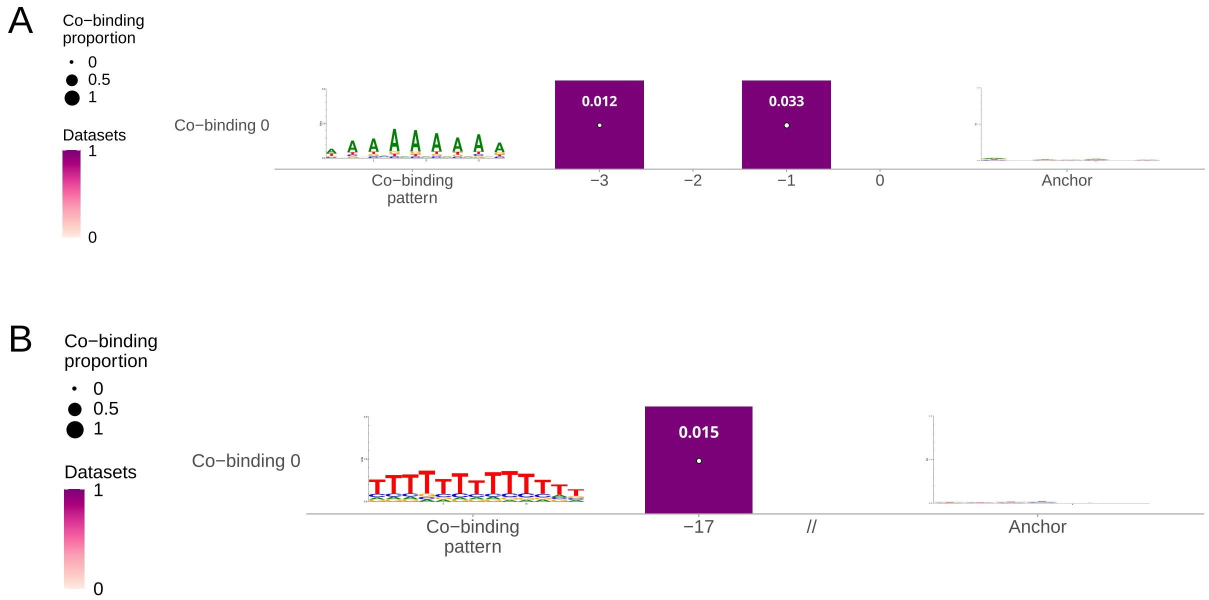

***Figure S41.* Examples of co-binding patterns found in random sequences matching the %GC composition of datasets for GATA2 (A) and HNF4G (B).** All patterns discovered in random regions are repeats of As or Ts and do not match the patterns found in the original datasets.

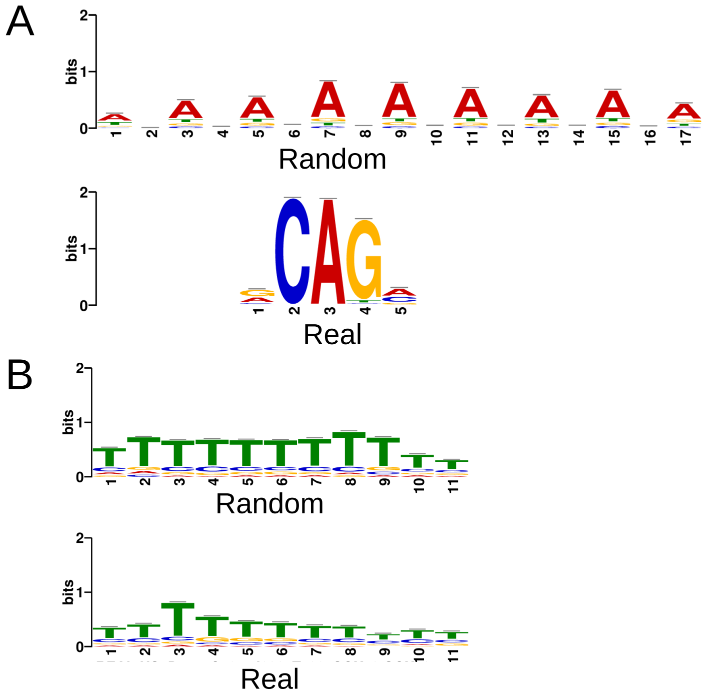

***Figure S42.* Motifs found in random region datasets compared to the ones found in real datasets.** Most patterns discovered in random regions did not match the ones found in the real datasets (Tomtom p-value > 0.05) (**A**). In only a few datasets, a similar pattern was found (Tomtom p-value < 0.05) (**B**), highlighting the limitation of A/T stretches discoveries.

REFERENCES

1. Karolchik,D., Hinrichs,A.S., Furey,T.S., Roskin,K.M., Sugnet,C.W., Haussler,D. and Kent,W.J. (2004) The UCSC table browser data retrieval tool. *Nucleic acids research*, **32**, D493–D496.

2. Khan,A., Riudavets Puig,R., Boddie,P. and Mathelier,A. (2021) BiasAway: Command-line and web server to generate nucleotide composition-matched DNA background sequences. *Bioinformatics*, **37**, 1607–1609.

3. Gupta,S., Stamatoyannopoulos,J.A., Bailey,T.L. and Noble,W.S. (2007) Quantifying similarity between motifs. *Genome biology*, **8**, 1–9.

4. Li,Y., Xu,Y., Ma,Z. and others (2017) Comparative analysis of the exon-intron structure in eukaryotic genomes. *Yangtze Medicine*, **1**, 50.

5. Mishra,A., Siwach,P., Misra,P., Dhiman,S., Pandey,A.K., Srivastava,P. and Jayaram,B. (2021) Intron exon boundary junctions in human genome have in-built unique structural and energetic signals. *Nucleic acids research*, **49**, 2674–2683.

6. Dozmorov,M.G., Adrianto,I., Giles,C.B., Glass,E., Glenn,S.B., Montgomery,C., Sivils,K.L., Olson,L.E., Iwayama,T., Freeman,W.M., *et al.* (2015) Detrimental effects of duplicate reads and low complexity regions on RNA-and ChIP-seq data. In *BMC bioinformatics*. Springer, Vol. 16, pp. 1–11.

7. Chung,D., Kuan,P.F., Li,B., Sanalkumar,R., Liang,K., Bresnick,E.H., Dewey,C. and Keleş,S. (2011) Discovering transcription factor binding sites in highly repetitive regions of genomes with multi-read analysis of ChIP-seq data. *PLoS computational biology*, **7**, e1002111.
